# Synthesis, Biological Studies, and *In Silico*-Driven Design of 8-Aminoquinoline-Based Sulfonamide Derivatives as Potential Antioxidant and Antimicrobial Agents

**DOI:** 10.34133/csbj.0032

**Published:** 2026-04-17

**Authors:** Ratchanok Pingaew, Apilak Worachartcheewan, Veda Prachayasittikul, Rungrot Cherdtrakulkiat, Supaluk Prachayasittikul, Somsak Ruchirawat, Virapong Prachayasittikul

**Affiliations:** ^1^ Department of Chemistry, Faculty of Science, Srinakharinwirot University, Bangkok 10110, Thailand.; ^2^ Department of Community Medical Technology, Faculty of Medical Technology, Mahidol University, Bangkok 10700, Thailand.; ^3^ Center for Research Innovation and Biomedical Informatics, Faculty of Medical Technology, Mahidol University, Bangkok 10700, Thailand.; ^4^ Department of Clinical Microbiology and Applied Technology, Faculty of Medical Technology, Mahidol University, Bangkok 10700, Thailand.; ^5^ Laboratory of Medicinal Chemistry, Chulabhorn Research Institute, Bangkok 10210, Thailand.; ^6^ Program in Chemical Sciences, Chulabhorn Graduate Institute, Bangkok 10210, Thailand.; ^7^ Center of Excellence on Environmental Health and Toxicology (EHT), Commission on Higher Education, Ministry of Education, Bangkok 10400, Thailand.

## Abstract

Eleven 8-aminoquinoline-based sulfonamide derivatives (**3**-**13**) were synthesized and experimentally investigated for their antioxidant effects (using superoxide dismutase [SOD] and 2,2-diphenyl-1-picrylhydrazyl [DPPH] assays) and antimicrobial activities (using agar dilution method). Most of the tested compounds were active antioxidants as indicated by SOD assay, affording SOD half-maximal inhibitory concentration in the range of 83.34 to 600.81 μM. However, their DPPH activities were considerably weak (%DPPH = 7.74% to 36.49%). Additionally, 7 compounds (**3**, **4**, **5**, **6**, **8**, **10**, and **12**) displayed growth-inhibiting effects against various gram-positive and gram-negative microbes (minimum inhibitory concentration values = ≤4 to 256 μg/ml). Quantitative structure–activity/property relationship (QSAR/QSPR) modeling was performed to obtain predictive models, including antioxidant QSAR (SOD and DPPH) and antimicrobial QSPR models. The constructed models displayed acceptable predictive performance and robustness (QSAR models: *R^2^_Tr_* = 0.9980 to 0.9996, *Q^2^*_*LOO-CV*_ = 0.9930 to 0.9978, *RMSE_Tr_* = 0.0121 to 0.1677, *RMSE*_*LOO-CV*_ = 0.0233 to 0.3830 and QSPR model: accuracy = 90.91% for training and 63.64% for leave-one-out cross-validation [LOO-CV] sets). The constructed models were subsequently utilized to guide rational design and predict activities of an additional set of 84 structurally modified compounds. Finally, newly designed compounds with promising predicted antioxidant activity (SOD: **13c**, **13f**, **8c**, **12e**, and **11c**; DPPH: **9b**, **6f**, **9c**, **11h**, and **3d**) as well as a set of 34 compounds predicted as active antimicrobial agents are summarized for potential investigations. Furthermore, key essential properties influencing bioactivities (electronegativity, polarizability, I-state, van der Waals volume, connectivity, and ionization potential) were also revealed for future beneficial design of the related compounds for medical applications.

## Highlights

• 8-Aminoquinoline-based sulfonamide derivatives (**3**-**13**) displayed antioxidant and antimicrobial activities.
• Significant descriptors influencing bioactivities were explored.
• Computational methods were used to construct predictive (QSAR and QSPR) models.
• *In silico* rational design of new 84 structurally modified compounds were generated.
• Potential newly designed compounds with the most promising predicted were highlighted.

## Graphical Abstract

**Figure:**
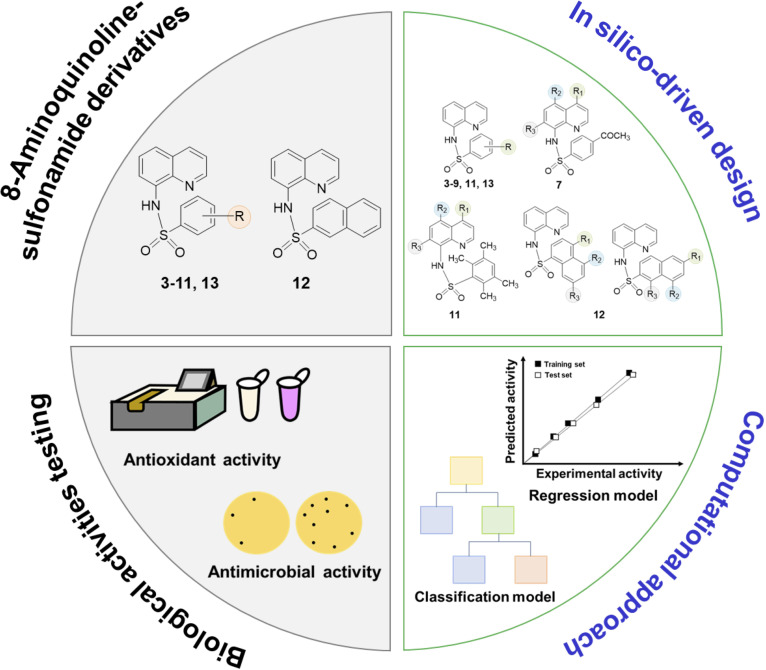

## Introduction

Free radicals are unstable and highly reactive molecules endogenously produced or received externally [1–3], These radicals are properly destroyed in our body by antioxidant defense to maintain oxidant–antioxidant balance [3,4]. However, in situations with overproduction of free radicals or impairment of antioxidant defenses, free radicals are excessively accumulated, leading to oxidative damage of the cellular components [1,2]. Oxidative stress is well recognized as a causative factor of multiple oxidative-related diseases and aging conditions (i.e., diabetes mellitus, cardiovascular diseases, cancer, and neurodegenerative diseases) [1–3]. Accordingly, the applications of antioxidant agents in therapeutics, prevention, and health promotion have gained considerable attention [4].

Infectious diseases are another concerning health problem worldwide [5], particularly the emergence of resistant bacterial species including gram-positive bacteria (i.e., *Staphylococcus aureus* and *Streptococcus pneumoniae*) and gram-negative bacteria (i.e., *Escherichia coli*, *Salmonella*, *Shigella*, *Klebsiella*, *Enterobacter*, and *Pseudomonas* spp.). Antimicrobial drug resistance in both community and hospital settings has also become an alarming issue [5–8]. This indicates an urgent need for discovery and development of novel potent antimicrobial agents for combating antimicrobial drug resistance crisis [9].

Heterocyclic compounds (i.e., quinolines) are attractive pharmacophores in medicinal chemistry for the design and development of diverse pharmacologically active compounds as well as the generation of newer derivatives and scaffolds [10,11]. 8-Aminoquinoline (8AQ), containing an amino group (–NH_2_) at 8-position on the quinoline ring, is a core structure found in antimalarial drugs (i.e., primaquine, pamaquine, and tafenoquine) widely known for their effectiveness against *Plasmodium* species. (i.e., *P. faciparum* and *P. vivax*) [12]. Its metabolic process in the mosquito was reported to indicate the promising role of the 8AQ scaffold in antimalarial drug discovery [13]. Additionally, 8AQ was reported as a ligand capable of coordinating with metal ions to give diverse bioactive metal complexes with medicinal values (i.e., antimicrobial, antioxidant, antimalarial, and anticancer properties) [12,14]. Apart from 8AQ, 4-aminoquinoline was also noted as a scaffold of many reported bioactive compounds displaying antimalarial, antimicrobial, and anticancer activities [10,15]. Sulfonamide is a scaffold widely found in many synthetic antimicrobial agents. It is well known as a versatile pharmacophore in drug design due to its metabolic stability [16]. Sulfonamide derivatives were also reported for their antioxidant and anticancer activities [17–19]. Molecular hybridization is one of the strategies in drug design for developing multifunctional bioactive compounds [20]. In recent drug development, great attention has been given to the design of diverse hybrid compounds, including quinoline–sulfonamide hybrids for combating antimicrobial drug resistance [21].

Cheminformatic approaches have been widely used to effectively facilitate successful drug design and discovery [22]. Particularly, these tools are employed to reveal structure–activity relationships essential for potent biological activities and preferable drug-like properties [23,24]. Quantitative structure–activity/property relationship (QSAR/QSPR) modeling is one of the computational methods commonly used in drug development to facilitate efficacious virtual design of new candidates [25–27]. The constructed models explored the relationship between the response end points such as biological and chemical activities (represented in numerical values or activities such as active and inactive). The successful stories of QSAR-driven rational design of new derivatives for therapeutics (i.e., antioxidant, neuroprotective, and anticancer agents) have recently been reported by our research group [28–30].

This study demonstrates the combined utilization of *in vitro* and *in silico* approaches for the efficacious design of new 8AQ-based sulfonamide analogs. Chemical synthesis was performed to obtain a series of 11 8AQ-based sulfonamide derivatives (compounds **3**-**13**), and their antioxidant and antimicrobial activities were experimentally investigated. The obtained bioactivity data together with chemical structures of the compounds were further used for constructions of predictive QSAR and QSPR models to reveal key essential structural features required for desirable activities. Finally, the constructed models were further applied to guide the rational design and predict bioactivities of an additional set of 84 structurally modified compounds

## Materials and Methods

### Chemistry

Analytical thin-layer chromatography was performed on silica gel 60 F_254_ aluminum sheets. ^1^H- and ^13^C-NMR (nuclear magnetic resonance) spectra were recorded on a Bruker AVANCE 300 or a Bruker AVANCE NEO 500 NMR spectrometer. High-resolution mass spectra (HRMS) were recorded on a Bruker Daltonics (micro time of flight [TOF]). Melting points were determined using a Griffin melting point apparatus and are uncorrected. *α*-Tocopherol, superoxide dismutase (SOD) (bovine erythrocytes), DPPH (2,2-diphenyl-1-picrylhydrazyl), Hepes, nitro blue tetrazolium chloride, L-methionine, riboflavin, and Triton X-100 were purchased from Sigma, USA as well as dimethyl sulfoxide (DMSO, 99.9%) from RCI Labscan, Thailand, and methanol from Merck, Germany. Ampicillin, ciprofloxacin, and tetracycline were procured from Sigma, USA; Mueller–Hinton broth (MHB) and Mueller–Hinton agar from Becton Dickinson, USA; and sodium chloride from Merck, Germany. Solvents are analytical grades.

### General procedure for the synthesis of quinoline sulfonamides (3-13)

Benzenesulfonyl chloride **2** (1 mmol) was added to a solution of 8AQ **1** (1 mmol) in pyridine (5 ml), and the mixture was stirred at room temperature for 20 to 36 h; the completion of the reaction was monitored by thin-layer chromatography (30% acetone:hexane). The reaction mixture was quenched with water (30 ml), and the precipitated product was filtered by vacuum filtration. The obtained sulfonamides were recrystallized from MeOH-CH_2_Cl_2_ to give the pure compounds (**3**-**13**).

#### 4-Fluoro-N-(quinolin-8-yl)benzenesulfonamide (3)

Brown solid. 74% Yield. Mp 130 to 131 °C. ^1^H NMR (300 MHz, DMSO-d_6_): δ (ppm) 7.31 (t, ^3^*J* = 8.8 Hz, 2H, *H*-3′ and *H*-5′), 7.53 (t, ^3^*J* = 8.8 Hz, 1H, *H*-6), 7.58 (dd, ^3^*J* = 8.3 Hz; and ^4^*J* = 4.2 Hz, 1H, *H*-3), 7.69 (d, ^3^*J* = 7.8 Hz, 2H, *H*-5 and *H*-7), 7.96 (dd, ^3^*J* = 8.8 Hz; and ^4^*J* = 5.2 Hz, 2H, *H*-2′ and *H*-6′), 8.35 (dd, ^3^*J* = 8.3 Hz; and ^4^*J* = 1.2 Hz, 1H, *H*-4), 8.84 (dd, ^3^*J* = 4.1 Hz; and ^4^*J* = 1.3 Hz, 1H, *H*-2), 10.10 (br s, 1H, N*H*). ^13^C NMR (75 MHz, DMSO-d_6_) δ 116.0 (d, ^2^*J*_CF_ = 23 Hz, *C*-3′ and *C*-5′), 117.2 (*C*-7), 122.1 (*C*-3), 123.2 (*C*-5), 126.4 (*C*-6), 128.0 (*C*-4a), 129.8 (d, ^3^*J*_CF_ = 10 Hz, *C*-2′ and *C*-6′), 133.2 (*C*-8), 135.8 (d, ^4^*J*_CF_ = 3 Hz, *C*-1′) 136.3 (*C*-4), 138.8 (*C*-8a), 149.2 (*C*-2), 164.2 (d, ^1^*J*_CF_ = 252 Hz, *C*-4′). HRMS-TOF: mass/charge ratio (*m/z*) [M + H]^+^ 303.0599 (calculated for C_15_H_12_FN_2_O_2_S: 303.0598).

The spectroscopic data are in accordance with the reported literature [31–33].

#### 4-Chloro-N-(quinolin-8-yl)benzenesulfonamide (4)

Brown solid. 72% Yield. Mp 128 to 129 °C. ^1^H NMR (300 MHz, DMSO-d_6_): δ (ppm) 7.50 to 7.59 (m, 4H, *H*-3, *H*-6, *H*-3′, and *H*-5′), 7.67 to 7.70 (m, 2H, *H*-5 and *H*-7), 7.89 (d, ^3^*J* = 8.5 Hz, 2H, *H*-2′ and *H*-6′), 8.35 (dd, ^3^*J* = 8.3 Hz; and ^4^*J* = 1.3 Hz, 1H, *H*-4), 8.83 (dd, ^3^*J* = 4.2 Hz; and ^4^*J* = 1.4 Hz, 1H, *H*-2), 10.09 (br s, 1H, N*H*). ^13^C NMR (75 MHz, DMSO-d_6_) δ 117.6 (*C*-7), 122.2 (*C*-3), 123.5 (*C*-5), 126.5 (*C*-6), 128.1 (*C*-4a), 128.8, 129.1 (*C*-2′, *C*-3′, *C*-5′, and *C*-6′), 133.3 (*C*-8), 136.4 (*C*-4), 137.9 (*C*-4′), 138.4 (*C*-1′), 139.0 (*C*-8a), 149.4 (*C*-2). HRMS-TOF: *m/z* [M + H]^+^ 319.0282 (calculated for C_15_H_12_ClN_2_O_2_S: 319.0303).

The spectroscopic data are in accordance with the reported literature [32,34].

#### 4-Bromo-N-(quinolin-8-yl)benzenesulfonamide (5)

Brown solid. 60% Yield. Mp 161 to 162 °C. ^1^H NMR (500 MHz, DMSO-d_6_): δ (ppm) 7.53 (t, ^3^*J* = 7.9 Hz, 1H, *H*-6), 7.58 (dd, ^3^*J* = 8.3 Hz; and ^4^*J* = 4.2 Hz, 1H, *H*-3), 7.66 to 7.70 (m, 4H, *H*-5, *H*-7, *H*-3′ and *H*-5′), 7.82 (d, ^3^*J* = 8.7 Hz, 2H, *H*-2′ and *H*-6′), 8.35 (dd, ^3^*J* = 8.3 Hz; and ^4^*J* = 1.6 Hz, 1H, *H*-4), 8.84 (dd, ^3^*J* = 4.2 Hz; and ^4^*J* = 1.7 Hz, 1H, *H*-2), 10.14 (br s, 1H, N*H*). ^13^C NMR (125 MHz, DMSO-d_6_) δ 117.8 (*C*-7), 122.3 (*C*-3), 123.5 (*C*-5), 126.7 (*C*-6), 127.0 (*C*-4′), 128.2 (*C*-4a), 129.0, 132.2 (*C*-2′, *C*-3′, *C*-5′, and *C*-6′), 133.5 (*C*-8), 136.6 (*C*-4), 139.0, 139.2 (*C*-8a and *C*-1′), 149.5 (*C*-2). HRMS-TOF: *m/z* [M + Na]^+^ 384.9625 (calculated for C_15_H_11_BrN_2_NaO_2_S: 384.9617).

The spectroscopic data are in accordance with the reported literature [31,33].

#### N-(quinolin-8-yl)-4-(trifluoromethyl)benzenesulfonamide (6)

Brown solid. 70% Yield. Mp 120 to 121 °C. ^1^H NMR (500 MHz, DMSO-d_6_): δ (ppm) 7.54 (t, ^3^*J* = 7.9 Hz, 1H, *H*-6), 7.56 (dd, ^3^*J* = 8.4 Hz; and ^4^*J* = 4.2 Hz, 1H, *H*-3), 7.69 (dd, ^3^*J* = 7.7 Hz; and ^4^*J* = 1.2 Hz, 1H, *H*-5), 7.71 (dd, ^3^*J* = 8.3 Hz; and ^4^*J* = 1.0 Hz, 1H, *H*-7), 7.87 (d, ^3^*J* = 8.3 Hz, 2H, *H*-3′ and *H*-5′), 8.08 (d, ^3^*J* = 8.2 Hz, 2H, *H*-2′ and *H*-6′), 8.35 (dd, ^3^*J* = 8.3 Hz; and ^4^*J* = 1.6 Hz, 1H, *H*-4), 8.80 (dd, ^3^*J* = 4.2 Hz; and ^4^*J* = 1.7 Hz, 1H, *H*-2), 10.41 (br s, 1H, N*H*). ^13^C NMR (125 MHz, DMSO-d_6_) δ 118.8 (*C*-7), 122.3 (*C*-3), 123.4 (q, ^1^*J*_CF_ = 252 Hz, *C*F_3_), 124.0 (*C*-5), 126.3 (q, ^3^*J*_CF_ = 3 Hz, *C*-3′ and *C*-5′), 126.7 (*C*-6), 127.9 (*C*-2′ and *C*-6′), 128.3 (*C*-4a), 132.6 (q, ^2^*J*_CF_ = 32 Hz, *C*-4′), 133.4 (*C*-8), 136.5 (*C*-4), 139.5 (*C*-8a), 143.7 (*C*-1′), 149.5 (*C*-2). HRMS-TOF: *m/z* [M + Na]^+^ 375.0377 (calculated for C_16_H_11_F_3_N_2_NaO_2_S: 375.0386).

The spectroscopic data are in accordance with the reported literature [31,32].

#### 4-Acetyl-N-(quinolin-8-yl)benzenesulfonamide (7)

Brown solid. 91% Yield. Mp 124 to 125 °C. ^1^H NMR (300 MHz, DMSO-d_6_): δ (ppm) 2.54 (s, 3H, C*H*_3_), 7.53 (t, ^3^*J* = 8.3 Hz, 1H, *H*-6), 7.58 (dd, ^3^*J* = 8.3 Hz; and ^4^*J* = 4.2 Hz, 1H, *H*-3), 7.68 to 7.71 (m, 2H, *H*-5 and *H*-7), 8.00 (d, ^3^*J* = 8.9 Hz, 2H, *H*-3′ and *H*-5′), 8.05 (d, ^3^*J* = 8.8 Hz, 2H, *H*-2′ and *H*-6′), 8.35 (dd, ^3^*J* = 8.3 Hz; and ^4^*J* = 1.6 Hz, 1H, *H*-4), 8.84 (dd, ^3^*J* = 4.2 Hz; and ^4^*J* = 1.6 Hz, 1H, *H*-2), 10.29 (br s, 1H, N*H*). ^13^C NMR (75 MHz, DMSO-d_6_) δ 26.9 (*C*H_3_), 117.5 (*C*-7), 122.3 (*C*-3), 123.5 (*C*-5), 126.6 (*C*-6), 127.2 (*C*-3′ and *C*-5′), 128.1 (*C*-4a), 128.8 (*C*-2′ and *C*-6′), 133.3 (*C*-8), 136.5 (*C*-4), 139.0 (*C*-8a), 139.8 (*C*-4′), 143.2 (*C*-1′), 149.4 (*C*-2), 197.7 (*C*=O). HRMS-TOF: *m/z* [M + Na]^+^ 349.0617 (calculated for C_17_H_14_N_2_NaO_3_S: 349.0617).

#### 4-Cyano-N-(quinolin-8-yl)benzenesulfonamide (8)

Brown solid. 74% Yield. Mp 121 to 122 °C. ^1^H NMR (300 MHz, DMSO-d_6_): δ (ppm) 7.54 (t, ^3^*J* = 8.0 Hz, 1H, *H*-6), 7.57 (dd, ^3^*J* = 8.4 Hz; and ^4^*J* = 4.3 Hz, 1H, *H*-3), 7.69 (d, ^3^*J* = 7.7 Hz, 1H, *H*-5), 7.73 (d, ^3^*J* = 8.3 Hz, 1H, *H*-7), 7.96 (d, ^3^*J* = 8.2 Hz, 2H, *H*-3′ and *H*-5′), 8.04 (d, ^3^*J* = 8.3 Hz, 2H, *H*-2′ and *H*-6′), 8.36 (dd, ^3^*J* = 8.3 Hz; and ^4^*J* = 1.2 Hz, 1H, *H*-4), 8.81 (dd, ^3^*J* = 4.2 Hz; and ^4^*J* = 1.2 Hz, 1H, *H*-2), 10.45 (br s, 1H, N*H*). ^13^C NMR (75 MHz, DMSO-d_6_) δ 115.3 (*C*-4′), 117.5 (*C≡*N), 118.9 (*C*-7), 122.3 (*C*-3), 124.1 (*C*-5), 126.6 (*C*-6), 127.6 (*C*-3′ and *C*-5′), 128.2 (*C*-4a), 133.1 (*C*-8, *C*-2′ and *C*-6′), 136.5 (*C*-4), 139.5 (*C*-8a), 143.9 (*C*-1′), 149.5 (*C*-2). HRMS-TOF: *m/z* [M + Na]^+^ 332.0461 (calculated for C_16_H_11_N_3_NaO_2_S: 332.0464).

The spectroscopic data are in accordance with the reported literature [31,32].

#### 2-Nitro-N-(quinolin-8-yl)benzenesulfonamide (9)

Yellow solid. 72% Yield. Mp 144 to 145 °C. ^1^H NMR (500 MHz, DMSO-d_6_): δ (ppm) 7.57 (t, ^3^*J* = 8.0 Hz, 1H, *H*-6), 7.61 (dd, ^3^*J* = 8.3 Hz; and ^4^*J* = 4.2 Hz, 1H, *H*-3), 7.74 to 7.76 (m, 2H, *H*-5 and *H*-7), 7.77 (td, ^3^*J* = 7.8 Hz; and ^4^*J* = 1.4 Hz, 1H, *H*-4′), 7.82 (td, ^3^*J* = 7.6 Hz; and ^4^*J* = 1.4 Hz, 1H, *H*-5′), 7.99 (dd, ^3^*J* = 7.9 Hz; and ^4^*J* = 1.3 Hz, 1H, *H*-6′), 8.18 (dd, ^3^*J* = 7.8 Hz; and ^4^*J* = 1.4 Hz, 1H, *H*-3′), 8.39 (dd, ^3^*J* = 8.3 Hz; and ^4^*J* = 1.6 Hz, 1H, *H*-4), 8.86 (dd, ^3^*J* = 4.2 Hz; and ^4^*J* = 1.6 Hz, 1H, *H*-2), 10.21 (br s, 1H, N*H*). ^13^C NMR (125 MHz, DMSO-d_6_) δ 117.5 (*C*-7), 122.6 (*C*-3), 124.0 (*C*-5), 125.2 (*C*-6′), 126.8 (*C*-6), 128.2 (*C*-4a), 130.6 (*C*-3′), 131.4 (*C*-1′), 132.7 (*C*-8), 132.9 (*C*-4′), 135.0 (*C*-5′), 136.7 (*C*-4), 138.9 (*C*-8a), 147.6 (*C*-2′), 149.7 (*C*-2). HRMS-TOF: *m/z* [M + Na]^+^ 352.0361 (calculated for C_15_H_11_N_3_NaO_4_S: 352.0362).

The spectroscopic data are in accordance with the reported literature [35].

#### 4-Nitro-N-(quinolin-8-yl)benzenesulfonamide (10)

Yellow solid. 87% Yield. Mp 158 to 159 °C. ^1^H NMR (300 MHz, DMSO-d_6_): δ (ppm) 7.52 to 7.58 (m, 2H, *H*-3, *H*-6), 7.71 (dd, ^3^*J* = 7.3 Hz; and ^4^*J* = 1.0 Hz, 1H, *H*-5), 7.73 (dd, ^3^*J* = 7.9 Hz; and ^4^*J* = 1.0 Hz, 1H, *H*-7), 8.14 (d, ^3^*J* = 8.8 Hz, 2H, *H*-3′ and *H*-5′), 8.29 (d, ^3^*J* = 8.8 Hz, 2H, *H*-2′ and *H*-6′), 8.35 (dd, ^3^*J* = 8.3 Hz; and ^4^*J* = 1.5 Hz, 1H, *H*-4), 8.80 (dd, ^3^*J* = 4.2 Hz; and ^4^*J* = 1.6 Hz, 1H, *H*-2), 10.56 (br s, 1H, N*H*). ^13^C NMR (75 MHz, DMSO-d_6_) δ 118.8 (*C*-7), 122.1 (*C*-3), 123.9 (*C*-5), 124.0 (*C*-3′ and *C*-5′), 126.4 (*C*-6), 128.1 (*C*-4a), 128.3 (*C*-2′ and *C*-6′), 132.9 (*C*-8), 136.3 (*C*-4), 139.4 (*C*-8a), 145.2 (*C*-1′), 149.3 (*C*-2), 149.7 (*C*-4′). HRMS-TOF: *m/z* [M + Na]^+^ 352.0359 (calculated for C_15_H_11_N_3_NaO_4_S: 352.0362).

The spectroscopic data are in accordance with the reported literature [32,33].

#### 2,3,5,6-Tetramethyl-N-(quinolin-8-yl)benzenesulfonamide (11)

Brown solid. 90% Yield. Mp 206 to 207 °C. ^1^H NMR (300 MHz, DMSO-d_6_): δ (ppm) 2.14 (s, 6H, 2 × C*H*_3_), 2.54 (s, 6H, 2 × C*H*_3_), 7.16 (s, 1H, *H*-4′), 7.40 to 7.48 (m, 2H, *H*-3 and *H*-6), 7.59 to 7.63 (m, 2H, *H*-5 and *H*-7), 8.36 (dd, ^3^*J* = 8.3 Hz; and ^4^*J* = 1.5 Hz, 1H, *H*-4), 8.89 (dd, ^3^*J* = 4.2 Hz; and ^4^*J* = 1.5 Hz, 1H, *H*-2), 9.72 (br s, 1H, N*H*). ^13^C NMR (75 MHz, DMSO-d_6_) δ 17.5 (2 × *C*H_3_), 20.4 (2 × *C*H_3_), 115.1 (*C*-7), 122.4 (*C*-3), 122.5 (*C*-5), 126.7 (*C*-6), 128.1 (*C*-4a), 133.6 (*C*-8), 134.7, 135.70 (*C*-2′, *C*-3′, *C*-5′, and *C*-6′), 135.72 (*C*-4), 136.6 (*C*-4′), 137.6 (*C*-1′), 138.2 (*C*-8a), 149.3 (*C*-2). HRMS-TOF: *m/z* [M + Na]^+^ 363.1136 (calculated for C_19_H_20_N_2_NaO_2_S: 363.1138).

#### N-(quinolin-8-yl)naphthalene-2-sulfonamide (12)

Brown solid. 60% Yield. Mp 138 to 139 °C. ^1^H NMR (500 MHz, DMSO-d_6_): δ (ppm) 7.50 (t, ^3^*J* = 8.0 Hz, 1H, *H*-6), 7.55 (dd, ^3^*J* = 8.3 Hz; and ^4^*J* = 4.2 Hz, 1H, *H*-3), 7.60 to 7.67 (m, 3H, *H*-5, *H*-6′ and *H*-7′) 7.74 (dd, ^3^*J* = 7.6 Hz; and ^4^*J* = 1.2 Hz, 1H, *H*-7), 7.92 (dd, ^3^*J* = 8.7 Hz; and ^4^*J* = 1.9 Hz, 1H, *H*-5′), 7.94 (dd, ^3^*J* = 8.0 Hz; and ^4^*J* = 1.0 Hz, 1H, *H*-8′), 7.99 (d, ^3^*J* = 8.8 Hz, 1H, *H*-4′), 8.10 (dd, ^3^*J* = 7.9 Hz; and ^4^*J* = 0.8 Hz, 1H, *H*-3′) 8.31 (dd, ^3^*J* = 8.3 Hz; and ^4^*J* = 1.6 Hz, 1H, *H*-4), 8.61 (d, ^4^*J* = 1.6 Hz, 1H, *H*-1′), 8.84 (dd, ^3^*J* = 4.2 Hz; and ^4^*J* = 1.7 Hz, 1H, *H*-2), 10.11 (br s, 1H, N*H*). ^13^C NMR (125 MHz, DMSO-d_6_) δ 116.8 (*C*-7), 122.3 (*C*-3), 122.4 (*C*-5′), 123.1 (*C*-5), 126.7 (*C*-6), 127.7 (*C*-7′), 127.8 (*C*-8′), 128.1 (*C*-4a), 128.3 (*C*-1′), 129.1 (*C*-6′), 129.2 (*C*-3′), 129.3 (*C*-4′), 131.5 (*C*-9′), 133.6 (*C*-8), 134.3 (*C*-10′), 136.5 (*C*-2′), 136.6 (*C*-4), 138.8 (*C*-8a), 149.4 (*C*-2). HRMS-TOF: *m/z* [M + Na]^+^ 357.0667 (calculated for C_19_H_14_N_2_NaO_2_S: 357.0668).

The spectroscopic data are in accordance with the reported literature [31,32,36].

#### 4-(Methylsulfonyl)-N-(quinolin-8-yl)benzenesulfonamide (13)

Brown solid. 92% Yield. Mp 178 to 179 °C. ^1^H NMR (300 MHz, DMSO-d_6_): δ (ppm) 3.23 (s, 3H, C*H*_3_), 7.55 (t, ^3^*J* = 8.1 Hz, 1H, *H*-6), 7.57 (dd, ^3^*J* = 8.3 Hz; and ^4^*J* = 4.5 Hz, 1H, *H*-3), 7.69 to 7.74 (m, 2H, *H*-5 and *H*-7), 8.03 (d, ^3^*J* = 8.4 Hz, 2H, *H*-2′ and *H*-6′), 8.15 (d, ^3^*J* = 8.5 Hz, 2H, *H*-3′ and *H*-5′), 8.36 (dd, ^3^*J* = 8.3 Hz; and ^4^*J* = 1.5 Hz, 1H, *H*-4), 8.81 (dd, ^3^*J* = 4.2 Hz; and ^4^*J* = 1.5 Hz, 1H, *H*-2), 10.45 (br s, 1H, N*H*). ^13^C NMR (75 MHz, DMSO-d_6_) δ 42.9 (*C*H_3_), 118.3 (*C*-7), 122.1 (*C*-3), 123.7 (*C*-5), 126.4 (*C*-6), 127.6, 127.7 (*C*-2′, *C*-3′, *C*-5′, and *C*-6′), 128.0 (*C*-4a), 133.0 (*C*-8), 136.3 (*C*-4), 139.2 (*C*-8a), 144.2 (*C*-1′), 144.4 (*C*-4′), 149.2 (*C*-2). HRMS-TOF: *m/z* [M + Na]^+^ 385.0291 (calculated for C_16_H_14_N_2_NaO_4_S_2_: 385.0287).

The spectroscopic data are in accordance with the reported literature [32].

### Antioxidant activity assay

The compounds were investigated for their antioxidant activities using DPPH and SOD assays as described below.

DPPH is a stable free radical that produces a violet solution in methanol. The DPPH radical is reduced by an antioxidant molecule, which donates an electron or a hydrogen atom, to give light-yellow product of DPPH [37]. The assay was initiated by adding 1 ml of 0.1 mM DPPH (dissolved in methanol) to the tested compounds dissolved in DMSO with the final concentration of 300 μg/ml, and the reaction mixture was incubated at room temperature in the dark for 30 min. The absorbance of the reaction was measured at 517 nm using an ultraviolet (UV)–visible spectrophotometer (UV-1610, Shimadzu). The standard antioxidant *α*-tocopherol was employed as a control compound, and DMSO solvent was used as the blank reaction. Experiments were performed in triplicate. The percentage of radical scavenging activity or DPPH was calculated using Eq. (1):

$$\text{DPPH}\left(\%\right)=\left[1-\frac{\mathrm{Abs}._{\text{sample}}}{\mathrm{Abs}._{\text{control}}}\right]\times 100$$ (1)

where *Abs*._control_ is the absorbance of the control reaction, and *Abs.*_sample_ is the absorbance of the tested compound.

Superoxide anion is a free radical that can be neutralized by SOD and SOD-like compounds. The SOD activity was evaluated using SOD assay [38]. The stock solution containing 27 ml of Hepes buffer (50 mM, pH 7.8), 1.5 ml of L-methionine (30 mg/ml), 1 ml of nitro blue tetrazolium chloride (1.41 mg/ml), and 750 μl of Triton X-100 (1 wt%) was prepared, and then 1 ml of the solution was added to the tested compounds dissolved in DMSO with the final concentration of 300 μg/ml. The reaction was initiated by adding 10 μl of riboflavin (44 mg/ml) and followed by illumination under a Philips Classic Tone lamp (60 W) in a light box for 7 min. The absorbance of the reaction was measured at 550 nm using a UV–visible spectrophotometer (UV-1610, Shimadzu). SOD from bovine erythrocytes served as a control substance. Experiments were performed in triplicate. The percentage of SOD activity was computed using Eq. (2):

$$\mathrm{SOD}\left(\%\right)=\left[1-\frac{\mathrm{Abs}._{\text{sample}}}{\mathrm{Abs}._{\text{control}}}\right]\times 100$$ (2)

where *Abs*._control_ is the absorbance of the control reaction and *Abs.*_sample_ is the absorbance of the tested compound.

The compounds exhibiting antioxidant activities (DPPH and SOD) greater than 50% at 300 μg/ml were further determined for their IC_50_ values by the 2-fold dilution method. The IC_50_ is half-maximal inhibitory concentration value for measurement of the compound concentration to inhibit a free radical by 50%. Plotting between antioxidant activities (%DPPH or %SOD) against compound concentrations was performed to obtain the corresponding IC_50_ values.

### Antimicrobial activity assay

The compounds were determined for antimicrobial activity using the conventional agar dilution method as described by Clinical & Laboratory Standards Institute (CLSI) guidelines [39] against 29 strains of microorganisms including reference strains and clinical isolates: gram-positive bacteria: *Staphylococcus aureus* ATCC 29213, *Staphylococcus aureus* ATCC 25923, *Staphylococcus epidermidis ATCC* 12228, *Enterococcus faecalis* ATCC 29212, *Enterococcus faecalis* ATCC 33186, *Micrococcus luteus* ATCC 10240, *Bacillus subtilis* ATCC 6633, *Corynebacterium diphtheria* NCTC 10356, *methicillin-resistant Staphylococcus aureus* JCSC 3063, *methicillin-resistant Staphylococcus aureus* N315, *methicillin-resistant Staphylococcus aureus* JCSC 4788, *Bacillus cereus*, and *Listeria monocytogenes*; gram-negative bacteria: *Escherichia coli* ATCC 25922, *Klebsiella pneumonia* ATCC 700603, *Serratia marcescens* ATCC 8100, *Salmonella typhimurium* ATCC 13311, *Salmonella choleraesuis* ATCC 10708, *Shewanella putrefaciens* ATCC 8671, *Achromobacter xylosoxidans* ATCC 27061, *Pseudomonas aeruginosa* ATCC 27853, *Pseudomonas stutzeri* ATCC 17587, *Salmonella enteritidis*, *Morganella morganii*, *Aeromonas hydrophilia*, *Citrobacter freundii*, and *Plesiomonas shigellloides*; and diploid fungus (yeast): *Candida albicans* ATCC 90028 and *Saccharomyces cerevisiae* ATCC 2601. The tested compounds and the reference antibacterial agents (i.e., ampicillin, ciprofloxacin, and tetracycline) were dissolved in DMSO, and the 2-fold dilution was further performed using MHB. Each dilution (1 ml) was added to the sterile 19-ml Mueller Hinton agar to give the final concentrations in the range of 256 to 4 μg/ml. The microorganisms were cultured in MHB at 37 °C for 24 h and suspended in 0.9% normal saline solution to adjust an optical density at 600 nm of 0.1 for a cell density of 1 × 10^8^ CFU/ml compared with the 0.5 McFarland turbidity standard. After that, 1 μl of microorganism suspensions (1 × 10^4^ CFU/spot) was inoculated onto the plates having a variety of compound concentrations using a multipoint inoculator and further incubated at 37 °C for 24 to 48 h. The plates containing DMSO and MHB without any antibacterial agents were simultaneously used as controls. The inhibition of microbial cell growth of tested compounds was investigated to obtain a minimum inhibitory concentration (MIC), which is the lowest concentration to inhibit the growth of microorganisms. In addition, the MIC quality control ranges, displayed in micrograms per milliliter of reference antibacterial agents according to CLSI, were investigated for the control system [40].

### QSAR analysis

#### Dataset and data preprocessing

Experimentally determined antioxidant activities (%DPPH and SOD IC₅₀) and antimicrobial activities (MIC, μM) were used as response end points for developing the QSAR and QSPR models, respectively. Three datasets were separately prepared to construct 3 models corresponding to each investigated assay. Each dataset contains (a) molecular descriptors (assigned as independent variables, *X_n_*) as structural representatives obtained by calculations and (b) bioactivity values (assigned as dependent variables, *Y*) obtained for experimental results. Experimental results obtained from DPPH and SOD assays were in numerical form (%DPPH or SOD IC₅₀), while those from the antimicrobial assays were in category form (active or inactive). Accordingly, 2 QSAR (DPPH and SOD) models and 1 QSPR (antimicrobial) model were constructed. For QSAR model construction, only active compounds displaying numerical %DPPH or SOD IC_50_ values were included in the datasets, whereas those inactive ones were discarded. Experimental antioxidant activity of SOD, expressed in IC_50_ (μM) values, was converted to pIC_50_ (M) by taking negative logarithmic transformation to the base of 10 [−log_10_(IC_50_)], whereas the antioxidant activity of DPPH exhibited in percentage (%) was directly utilized. Therefore, the compound displaying a high pIC_50_ (low IC_50_) value is interpreted to have high antioxidant activity. Regarding antimicrobial activity, the compounds were classified as active or inactive based on their MIC values against the tested microorganisms.

#### Calculation of quantum chemical and molecular descriptors

Molecular structures of the compounds were constructed using Chemdraw Pro13 software (PerkinElmer, USA) and subjected to GaussView software [41]. The structures were initially saved as *.smi files and converted to *.mol files using OpenBabel version 2.3.2. The prepared *.mol files were subsequently used as input files for computing 2-dimensional (2D) descriptors using PaDEL [42] and Mold^2^ [43] software to obtain a set of 1,444 PaDEL 0D-2D descriptors and 777 Mold2 2D descriptors, respectively.

Geometrical optimization was performed using Gaussian 09, Revision A.02 [44] software at the semiempirical level using Austin Model 1, followed by density functional theory calculation using Becke’s 3-parameter hybrid method and the Lee–Yang–Parr correlation functional (B3LYP) together with the 6-31 g(d) basis set. The low-energy conformers of the compounds were received and were consequently used to compute a set of 13 quantum chemical descriptors including the total energy (*E*_total_) of the molecule, the highest occupied molecular orbital energy (*E*_HOMO_), the lowest unoccupied molecular orbital energy (*E*_LUMO_), the total dipole moment (*μ*) of the molecule, the electron affinity, the ionization potential, the energy difference of HOMO and LUMO (HOMO–LUMO_Gap_), Mulliken electronegativity (*χ*), hardness (*η*), softness (*S*), electrophilicity (*ω*), electrophilic index (*ω_i_*), and the mean absolute atomic charge (*Q_m_*) [17]. The optimized structures were further used as input files for calculating a set of 3,224 molecular descriptors using Dragon software, version 5.5 [45]. Descriptor variables with constant and redundant values were initially removed by Dragon software to give a remaining set of 1,308 molecular descriptors. Finally, a total set of 3,542 descriptors (including 1,308 molecular descriptors, 13 quantum chemical descriptors, 1,444 PaDEL 0D-2D descriptors, and 777 Mold2 2D descriptors) was obtained and subsequently subjected to a feature selection process.

#### Feature selection

A final set of 3,542 calculated descriptors underwent a feature selection process to select only a set of significant descriptors that are correlated with bioactivities using stepwise multiple linear regression (MLR) (SPSS Statistics 18.0, SPSS Inc., USA) or by automatic variable selection procedure (CfsSubsetEval combined with the BestFirst) as implemented in WEKA software, version 3.4.5 [46] or by the UFS algorithm (UFS software package, version 1.8) [47]. The selected descriptors were further investigated for their intercorrelations. Pearson’s correlation coefficient (*r*) values between each pair of selected descriptors within the same model were calculated using SPSS Statistics 18.0 (SPSS Inc., USA), and a cutoff value of |*r*| ≤ 0.9 was used to determine their independence. Finally, the selected descriptors were further used to prepare final datasets for model construction.

#### Model construction

##### MLR

Two QSAR models (i.e., DPPH and SOD) were constructed using the MLR method. Molecular descriptors were assigned as independent variables (*X*), and biological activity values were assigned as dependent variables (*Y*). The QSAR models were generated according to Eq. (3):

$$Y=m_{1}x_{1}+m_{2}x_{2}+\ldots +m_{n}x_{n}+b$$ (3)

where *Y* is the biological activity values of the compounds, *m* is the regression coefficient values of the descriptors *x*, and *b* is the intercept. The MLR method was performed using Weka software, version 3.4.5 [46].

##### Decision tree analysis

An antimicrobial QSPR model was constructed using decision tree analysis. The decision tree is a supervised method that generates a set of if-then rules. It finds and explores the most essential independent variables (*X*) to classify compounds with interest activity (*Y*). The tree exhibited the top–down manner starting from the root node through the internal nodes using the independent variables and finally to the terminal leaf nodes for the class prediction [48–50]. Herein, the J48 decision tree algorithm, a derivative of the C4.5 algorithm implemented in Weka software version 3.4.5 [46], was used to construct the QSPR model for classifying the end points of the compounds of interest as active or inactive.

#### Generation of dataset

The dataset was divided into training and testing sets. The training set was used to generate the predictive models, whereas the testing set was employed to validate the models. The testing set was generated by leave-one-out cross-validation (LOO-CV), in which 1 sample was left out from the whole dataset (*N*) to be used as a testing set while the remaining samples (*N* − 1) were used as the training set. This principle was iteratively continued until every sample was used as the testing set [17]. Additionally, 5-fold cross-validation (5-fold-CV) was performed to evaluate and compare the predictive performance of the QSAR/QSPR models with the results obtained from LOO-CV [30].

#### Evaluation of model predictive performance

Statistical parameters were calculated to validate the predictive performance of the constructed models. For the QSAR models, correlation coefficient parameters (i.e., squared correlation coefficient $\left[R_{\mathrm{Tr}}^{2}\right]$ and cross-validated *R^2^* [$Q_{LOO-CV}^{2}$ and *Q^2^*_*5-fold-CV*_] for training, LOO-CV, and 5-fold-CV sets, respectively) and root mean square error (RMSE) were calculated to determine the predictive error of the models (i.e., training [*RMSETr*], LOO-CV [*RMSE*_*LOO-CV*_], and 5-fold-CV [*RMSE*_*5-fold-CV*_] sets) [17,30]. Furthermore, adjusted *R^2^* (*R^2^_adj_*), mean absolute error (MAE), and concordance correlation coefficient (CCC) were computed to evaluate the robustness of the constructed QSAR models [51–53]. For the QSPR decision tree model, the classification model provided descriptor-based cutoff values to classify compounds as active or inactive. Statistical indices including accuracy, precision, recall, and F-measure were used to assess the predictive performance of the model (i.e., training, LOO-CV, and 5-fold-CV sets) [54].

#### Application of the constructed QSAR models for guiding design of new derivatives

After validating their reliability, the constructed models were used for the rational design of new derivatives, in which descriptor variables presented in the models were used for guiding structural modification on the core structures of the selected prototypes (compounds **3**, **4**, **5**, **6**, **7**, **8**, **9**, **11**, **12**, and **13**) to finally give an additional set of 84 newly designed derivatives. All newly designed compounds were drawn, geometrically optimized, and calculated for their descriptor values in the same manner as those of prototypes (as described in the “Calculation of quantum chemical and molecular descriptors” section). The obtained key descriptor values were subsequently used for predicting their SOD and DPPH activities using the constructed models. A similar process was performed by applying the constructed antimicrobial QSPR model for predicting antimicrobial classes (active or inactive) of the newly designed compounds.

## Results and Discussion

### Chemistry

**Fig. 1.**
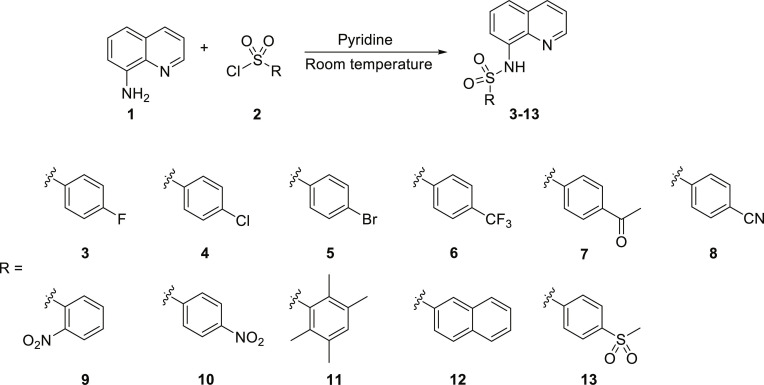
Synthesis of 8-aminoquinoline (8AQ)-based sulfonamide derivatives (3-13) [33].

A set of quinoline–sulfonamides (**3-13**) was synthesized by *N*-sulfonylation of 8AQ **1** with the corresponding benzenesulfonyl chloride **2** in pyridine at room temperature in moderate to good yields (60% to 92%) as depicted in Fig. 1 [33]. The structures of the sulfonamides (**3-13**) were confirmed by means of spectroscopic methods, namely, HRMS, ^1^H NMR, and ^13^C NMR. All sulfonamides had molecular ion peaks consistent with their molecular formulas. ^1^H NMR spectra of sulfonamides (**3-13**) displayed signals of aromatic protons of both the quinoline ring and the sulfonyl part (R), indicating that the *N*-sulfonation products were formed. The spectroscopic data of the known compounds **3**-**6**, **8**-**10**, and **12**-**13** are in accordance with those reported in the literature [31–36]. In ^1^H NMR, these derivatives showed the NH signal of sulfonamide group at a chemical shift of about 10 ppm. The quinoline protons at the C-2 and C-4 positions typically appeared as doublet of doublets (dd) at the low-field chemical shift around 8.8 and 8.3 ppm, respectively. The title sulfonamides (**3**-**13**) have a common R group (benzene) substituted with various substituents (X = F, Cl, Br, CF_3_, CN, COCH_3_, NO_2_, SO_2_CH_3_, and 2,3,5,6-tetramethyl) at *ortho* (*o*), *meta* (*m*), and *para-* (*p*) positions, except for compound **13**, R = naphthalene ring. ^1^H and ^13^C NMR spectral data of compounds **3**-**13** are provided in Supplementary Materials (Figs. S1 to S22).

### Antioxidant activities

The synthesized 8AQ-based sulfonamides (**3**-**13**) were evaluated for their antioxidant activities using DPPH and SOD assays to measure their scavenging capabilities toward DPPH free radicals and superoxide anions (O_2_^•-^), respectively.

The DPPH assay is a simple method to investigate radical scavenging activities, known as DPPH activity [37]. All tested compounds (**3**-**13**; Table 1) exhibit weak DPPH radical scavenging activity, with inhibition values ranging from 7.74% to 36.49% at 300 μg/ml. Since none of the compounds achieved 50% inhibition, IC_50_ values were not determined. Among all, *o*-NO_2_-substituted compound **9** and tetramethyl-substituted compound **11** (%DPPH = 36.49% and 22.43%, respectively) exhibited the most potent radical scavenging effect, whereas a bromo-containing compound **5** displayed the weakest activity (%DPPH = 7.74%). Considering the halogen-containing compounds (Table 1), compounds **3**, **4**, and **5** with mono-halogen substitution (X = F [10.09%], Cl [9.26%], and Br [7.74%], respectively) showed lower activity than the trifluoro compound **6** (X = CF_3_, 12.06%). However, all tested compounds exhibited considerably lower DPPH radical scavenging activity than that of the standard antioxidant *α*-tocopherol (IC₅₀ = 12.82 μM), suggesting their limited potential for effective therapeutics.

**Table 1.** Antioxidant activities of 8AQ-based sulfonamides (3-13). The standard antioxidant *α*-tocopherol was used as a control in DPPH assay (IC_50_ = 12.82 μM), and SOD from bovine erythrocytes was used as a control in SOD assay (IC_50_ = 0.002 μM).

Compound	DPPH activity (%) ^a^	SOD activity (IC_50_, μM)
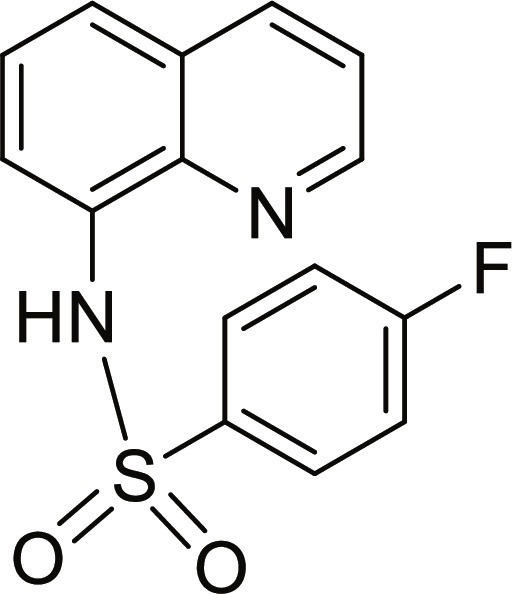	10.09	153.56
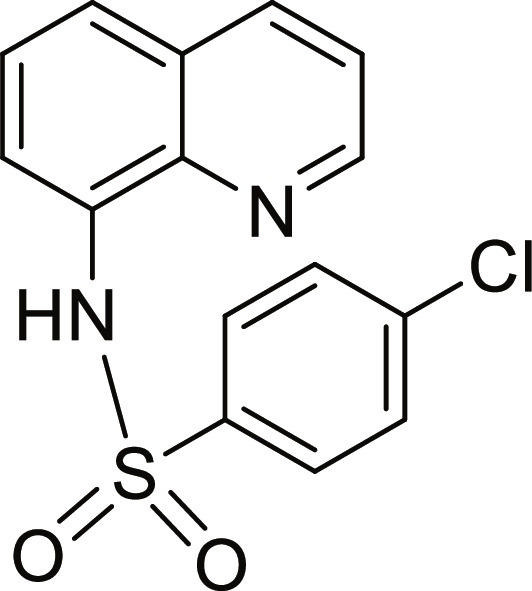	9.26	ND ^b^
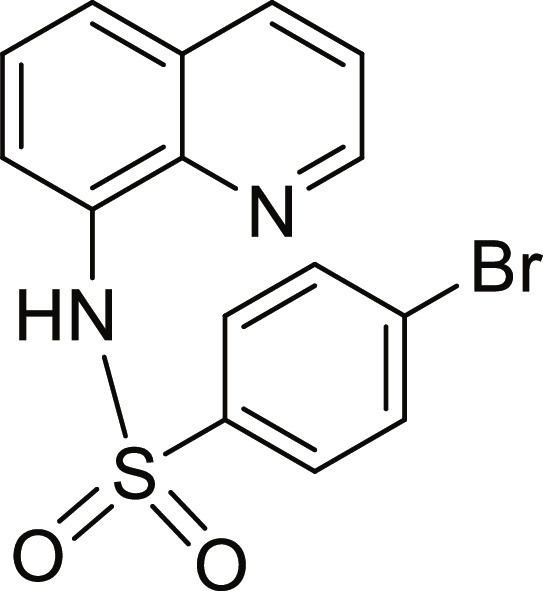	7.74	212.58
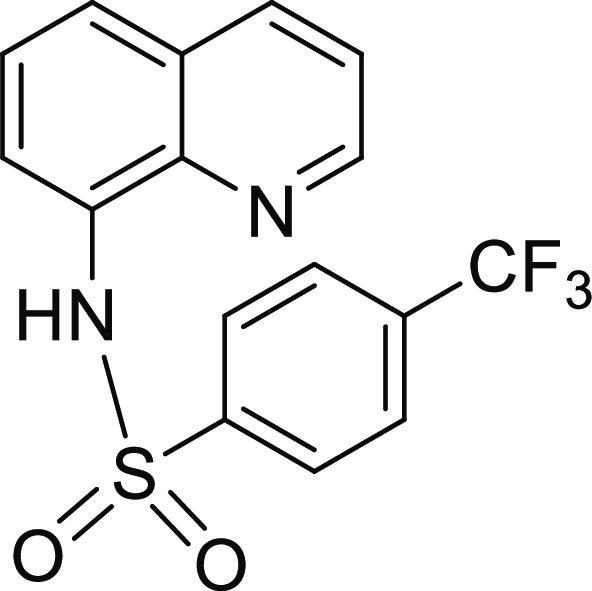	12.06	247.39
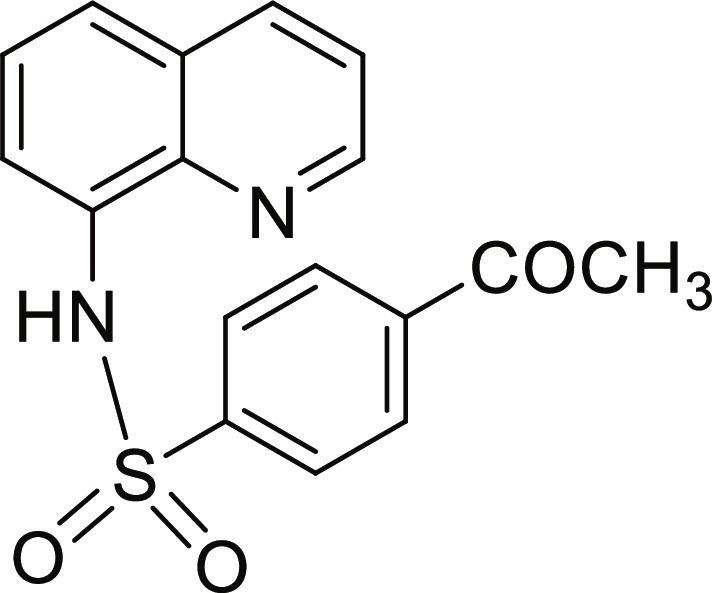	9.79	250.37
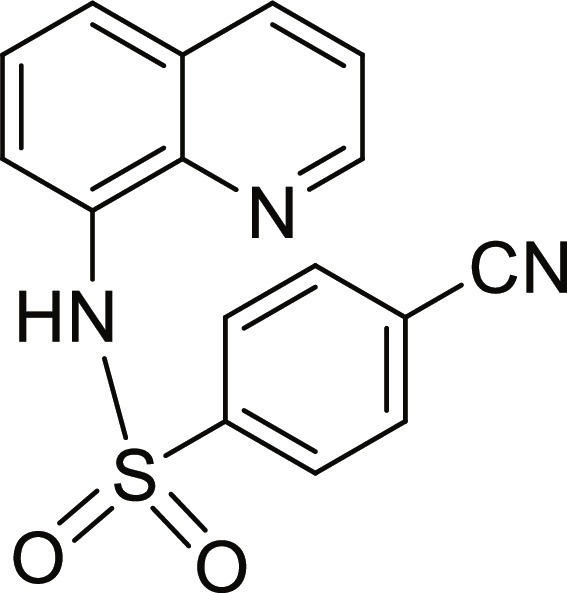	12.53	96.07
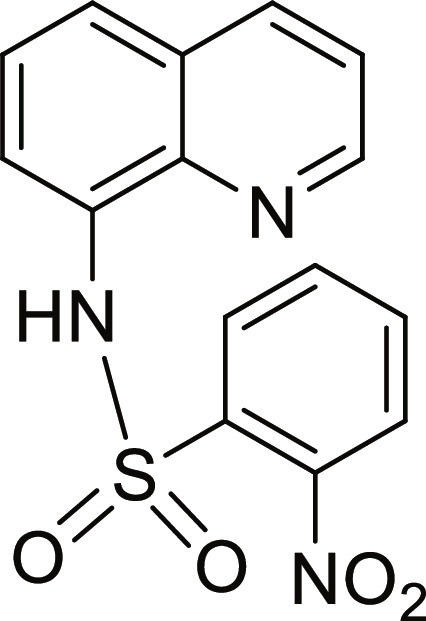	36.49	254.58
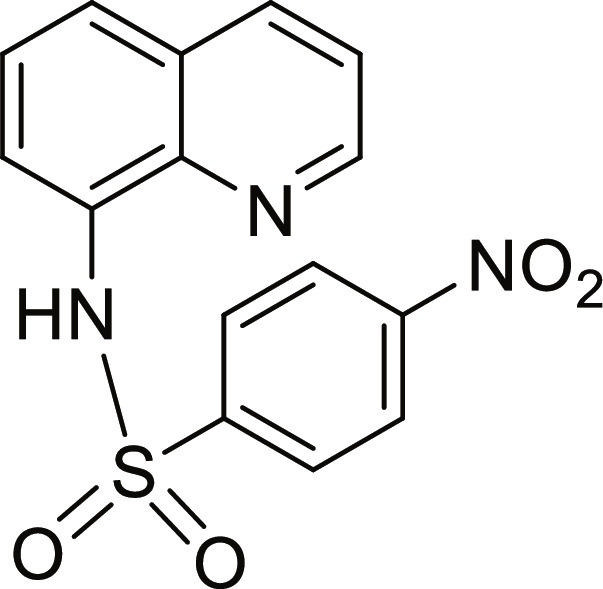	19.06	448.75
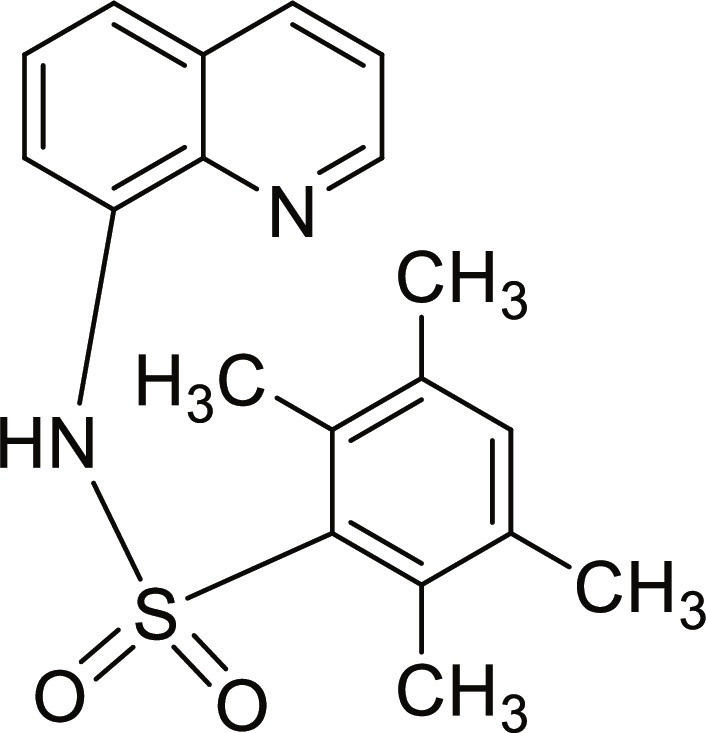	22.43	530.93
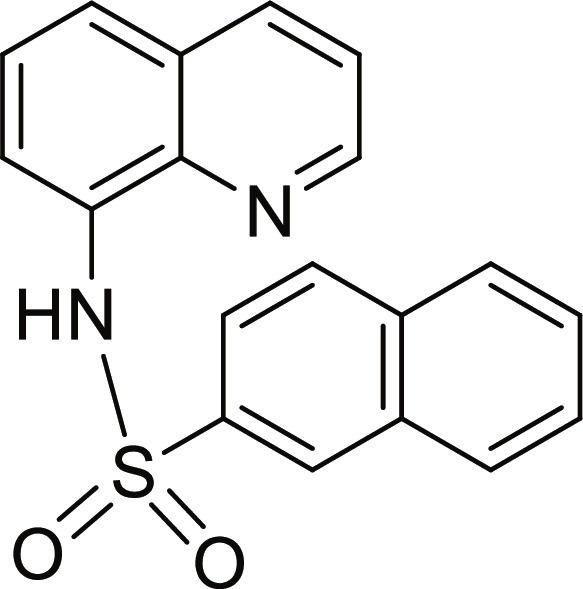	13.88	83.34
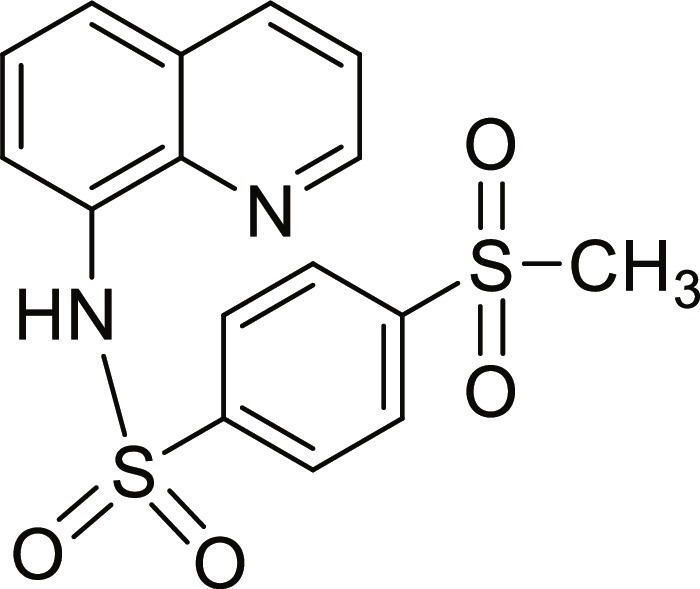	17.33	600.81

8AQ, 8-aminoquinoline; SOD, superoxide dismutase; ND, not determined

^a^ Compounds were tested at 300 μg/ml, and half-maximal inhibitory concentration (IC_50_) values were not determined because compounds exhibited 2,2-diphenyl-1-picrylhydrazyl (DPPH) activity <50%.

^b^ Compound **4** (17.88% SOD) displayed SOD activity <50%; therefore, IC_50_ was not determined.

The synthesized compounds (**3**-**13**) were investigated for their abilities to scavenge superoxide anion (SOD-mimic) activity [38]. All tested compounds, except for the chloro-containing compound **4** (%SOD = 17.88% at 300 μg/ml), showed SOD-mimic activity greater than 50%. Therefore, their IC₅₀ values were subsequently determined, providing the SOD IC_50_ values in the range of 83.34 to 600.81 μM (Table 1). Naphthalene-substituted compound **12** displayed the highest SOD activity (IC_50_ = 83.34 μM), followed by CN-substituted compound **8** (IC_50_ = 96.07 μM). However, these compounds displayed considerably lower activity when compared to that of the control SOD enzyme (IC₅₀ = 0.002 μM), suggesting their modest SOD activity.

Comparing the 2 antioxidant assays, the tested compounds (**3**-**13**) exhibited more potent SOD activity than DPPH activity. The isomeric effect of NO_2_ substition on the benzene ring was noted for 2 NO_2_ derivatives **9** and **10**. It was observed that the substitution of NO_2_ on the *o-*position of the benzene ring provided better antioxidant effects (both DPPH and SOD) than those with the *p-*position. Compound **9** (X = *o-*NO_2_, %DPPH = 36.49%, SOD IC_50_ = 254.58 μM) displayed more potent SOD and DPPH activities than compound **10** (X = *p-*NO_2_, %DPPH = 19.06%, SOD IC_50_ = 448.75 μM), as shown in Table 1.

### Antimicrobial activity

All synthesized compounds were investigated for their antimicrobial activities against gram-positive and gram-negative bacteria as well as diploid fungi using the agar dilution method [39]. It is an assay used to evaluate the ability of compounds to inhibit the growth of microorganisms. The mechanism of action of the tested compounds was not investigated because the main objective was to screen for growth-inhibiting activity.

The MIC quality control ranges for reference antibacterial agents (including ampicillin, ciprofloxacin, and tetracycline) were performed to evaluate the control system according to CLSI [40]. It was found that the ampicillin displayed an MIC value of 8 μg/ml to inhibit the growth of *E. coli* ATCC 25922 and an MIC value of 2 μg/ml for *E. faecalis* ATCC 29212, in which the acceptable MIC quality control ranges of *E. coli* ATCC 25922 and *E. faecalis* ATCC 29212 were 2 to 8 μg/ml and 0.5 to 2 μg/ml, respectively. The ciprofloxacin exhibited MIC values of 0.25 μg/ml against *S. aureus* ATCC 29213 and MIC values of 0.5 μg/ml against *E. faecalis* ATCC 29212 and *P. aeruginosa* ATCC 27583. The acceptable MIC quality control ranges of ciprofloxacin against the microorganism were 0.12 to 0.5 μg/ml, 0.25 to 2 μg/ml, and 0.25 to 1 μg/ml for *S. aureus* ATCC 29213, *E. faecalis* ATCC 29212, and *P. aeruginosa* ATCC 27583, respectively. The tetracycline inhibited the growth of *S. aureus* ATCC 29213, *E. faecalis* ATCC 29212, *E. coli* ATCC 25922, and *P. aeruginosa* ATCC 27583 at MIC values of 1, 32, 2, and 32 μg/ml with the acceptable MIC quality control ranges of 0.12 to 1 μg/ml, 8 to 32 μg/ml, 0.5 to 2 μg/ml, and 8 to 32 μg/ml, respectively. Furthermore, both DMSO solvent and MHB have no effect on microbial growth, and no contaminations were observed. Collectively, the results from MIC quality control testing confirmed the reliability of the prepared system for compounds’ investigations and indicated the interpretability of the results.

The MIC values expressed in micrograms per milliliter (μg/ml) are provided in Table 2. Most of the 8AQ-sulfonamides exhibited preferable antimicrobial activity, except for 4 inactive compounds (**7**, **9**, **11**, and **13**). The MIC values in units of μg/ml were converted (using calculated molecular weight from Dragon software, version 5.5) to micromolar units (μM) for comparing the effective activity of the tested compounds. It was found (Table 2) that compounds **3**, **4**, **5**, and **6** with halogen groups displayed excellent antimicrobial activity (MIC = ≤4 to 128 μg/ml or ≤11.01 to 423.35 μM), followed by compounds **8** with nitrile (CN) group (MIC = ≤4 to 128 μg/ml or ≤12.93 to 413.74 μM), compound **10** with nitro group (NO_2_) (MIC = 128 μg/ml or 388.63 μM), and naphthalene-substituted compound **12** (MIC = 256 μg/ml or 765.50 μM). Most of the active compounds (**3**, **4**, **5**, **6**, **8**, and **10**) showed significant growth-inhibiting effects against gram-positive bacteria (i.e., *S. aureus* ATCC 29213, *S. aureus* ATCC 25923, *S. epidermidis* ATCC 12228, *M. luteus* ATCC 10240, *B. subtilis* ATCC 6633, MRSA JSCS 4788, MRSA N315, MRSA JCSC 3063, *B. cereus*, and *L. monocytogenes*, MIC ≤4 to 128 μg/ml or ≤11.01 to 423.35 μM), whereas compound **12** (R = naphthalene) mainly displayed antimicrobial activity against gram-negative bacteria (i.e., *E. coli* ATCC 25922, *S. typhimurium* ATCC 13311, *P. stutzeri* ATCC 17587, *S. enteritidis*, *M. morganii*, *A. hydrophila*, and *C. freundii*, MIC = 256 μg/ml or 765.50 μM). Interestingly, most of the tested compounds inhibited growth of the methicillin-resistant *Staphylococcus aureus* (MRSA) providing the MIC range of ≤4 to 64 μg/ml or ≤11.01 to 211.68 μM. In contrast, compounds **7**, **9**, **11**, and **13** (X = *p*-COCH_3_, *o*-NO_2_, 2,3,5,6-tetra-CH_3_, and *p*-SO_2_CH_3_) exhibited inactive antimicrobial activities.

**Table 2.** Antimicrobial activity (MIC) of 8AQ-based sulfonamides (3-13). Compounds 7, 9, 11, and 13 displayed no antimicrobial activity at a concentration of 256 μg/ml. Molecular weight (MW) calculated from Dragon software, version 5.5 of compounds 3, 4, 5, 6, 7, 8, 9, 10, 11, 12, and 13 are 302.35, 318.80, 363.25, 352.36, 326.40, 309.37, 329.36, 329.36, 340.48, 334.42, and 362.46 g/mol, respectively. The MW of the compounds was used to convert MIC values in μg/ml to μM.

Compound	MIC		Microorganism
	μg/ml	μM	
**3**	128	423.35	*B. cereus*
	64	211.68	*S. aureus* ATCC 29213, *S. aureus* ATCC 25923, *M. luteus* ATCC 10240, MRSA JCSC 4788
	8	26.46	MRSA N315
	≤4	≤13.23	*B. subtilis* ATCC 6633, MRSA JCSC 3063
**4**	64	200.75	*S. aureus* ATCC 29213, *S. epidermidis* ATCC 12228
	16	50.19	*P. shigelloides*, *L. monocytogenes*
	≤4	≤12.55	*S. aureus* ATCC 25923, *M. luteus* ATCC 10240 *B. subtilis* ATCC 6633, *B. cereus*, MRSA JCSC 3063, MRSA N315, MRSA JCSC 4788
**5**	8	22.02	MRSA N315
	≤4	≤11.01	*B. subtilis* ATCC 6633, MRSA JCSC 3063
**6**	≤4	≤11.35	*S. aureus* ATCC 25923, *B. subtilis* ATCC 6633, *B. cereus*, *P. shigelloides*, *L. monocytogenes*, MRSA JCSC 3063, MRSA N315, MRSA JCSC 4788
**8**	128	413.74	*A. hydrophila*
	≤4	≤12.93	*S. aureus* ATCC 25923, *B. subtilis* ATCC 6633, *B. cereus*, MRSA JCSC 3063, MRSA N315
**10**	128	388.63	*B. cereus*
**12**	256	765.50	*E. coli* ATCC 25922, *S. typhimurium* ATCC 13311, *P. stutzeri* ATCC 17587, *S. enteritidis type C*, *M. morganii*, *A. hydrophila*, *C. freundii*

MIC: Minimum inhibitory concentration is the lowest concentration to inhibit the growth of microorganisms; 8AQ, 8-aminoquinoline

In overview, the compounds containing halogen atom (F, Cl, Br, and CF_3_), nitrile (CN), and naphthalenyl (C_10_H_7_) are active antimicrobial agents. Among others, compound **3** (X = F) and compound **4** (X = Cl) displayed the most effective antimicrobial activity against various micoorganisms (MIC range: compound **3** = ≤4 to 128 μg/ml or ≤13.23 to 423.35 μM and compound **4** = ≤4 to 64 μg/ml or ≤12.55 to 200.75 μM). The halogen atoms, especially F and Cl atoms, are well recognized as key functional groups found in several classes of antimicrobial scaffolds (i.e., *β*-lactam antibiotics, sulfa drugs, and aminoglycosides). Halogenated antimicrobial agents displayed diverse inhibitory mechanisms against microorganisms via acting as inhibitors of cell wall synthesis, cell proliferation, and protein synthesis [55].

Although 8AQ itself has been reported as an inactive antimicrobial agent [12], its notable antimicrobial effect as well as antimalarial and antioxidant properties were observed when it was combined with other compounds (i.e., 5-iodouracil and 5-nitrouracil) to form mixed-ligand metal complexes (i.e., copper [Cu], manganese [Mn], and nickel [Ni] complexes) [12,14,56]. Furthermore, 8AQ derivatives displayed antimalarial, anticancer, and antioxidant activities [15,57–59]. These previously reported works supported our in vitro results, which suggested that some of the synthesized 8AQ-sulfonamide hybrids could be potentially further developed as antioxidant and antimicrobial agents. The biological activities, including antioxidant and antimicrobial activities, of the 8AQ derivatives have been noted to associate with potential mechanisms such as redox cycling, oxidative stress induction, and metal chelation. Notably, its metal-chelating ability causes the disruption of the key biochemical components within the microorganisms, leading to the generation of reactive oxygen species or the sequestration of essential metal cofactors, ultimately inhibiting microbial growth [12].

### QSAR and QSPR models

QSAR and QSPR models are computational methods commonly used to reveal the relationships between structural information and bioactivity values of the studied compounds [50]. Among others, the MLR algorithm is practically used in the drug design area due to its interpretable nature [60]. Many QSAR models have been reported for elucidating structure–activity relationships of diverse bioactive compounds toward several bioactivities such as anticancer, antioxidant, and antimicrobial activities [61–63].

Feature selection was performed to select only a set of informative descriptors to be included in the final datasets for model construction. Definitions of the selected descriptors selected for construction of DPPH (4 descriptors: ATS5s, GATS1e, Mor04p, and Mor24u), SOD (4 descriptors: R1v, AATS8p, B08[C–O], and D21), and antimicrobial (2 descriptors: X4sol and VR2_Dzi) models are provided in Table 3, and their values are provided in Supplementary Materials (Tables S1 to S3). Additionally, the intercorrelation matrix calculated by Pearson’s correlation coefficient (cutoff values of |*r*| > 0.9) indicated that these descriptors are independent predictors (Supplementary Materials, Tables S4 to S6).

**Table 3.** Definition of significant descriptors for QSAR modeling

Activity	Symbol	Description	Class	Software
DPPH (%)	ATS5s	Broto–Moreau autocorrelation of lag 5 (log function) weighted by I-state	2D autocorrelations	PaDEL
	GATS1e	Geary autocorrelation of lag 1 weighted by Sanderson electronegativity	2D autocorrelations	Dragon
	Mor04p	Signal 04 / weighted by polarizability	3D-MoRSE descriptors	Dragon
	Mor24u	3D-MoRSE - signal 24 / unweighted	3D-MoRSE descriptors	Dragon
SOD (pIC_50_)	R1v	R autocorrelation of lag 1 / weighted by van der Waals volume	GETAWAY descriptors	Dragon
	AATS8p	Average Broto–Moreau autocorrelation - lag 8 / weighted by polarizabilities	Autocorrelation descriptors	PaDEL
	B08[C–O]	Presence/absence of C–O at topological distance 8	2D atom pairs	Dragon
	D211	Average vertex connectivity order-5 index	2D descriptor	Mold^2^
Antimicrobial (MIC)	X4sol	Solvation connectivity index of order 4	Connectivity indices	Dragon
	VR2_Dzi	Normalized Randic-like eigenvector-based index from Barysz matrix / weighted by first ionization potential	Barysz matrix descriptor	PaDEL

SOD, superoxide dismutase; DPPH, 2,2-diphenyl-1-picrylhydrazyl; MIC, minimum inhibitory concentration; QSAR, quantitative structure–activity relationship; 2D, 2-dimensional; 3D-MoRSE, 3D-molecule representation of structures based on electron diffraction; GETAWAY, geometry, topology, and atom-weights assembly; pIC_50_, the negative base 10 logarithm of the SOD IC_50_ (μM)

**Fig. 2.**
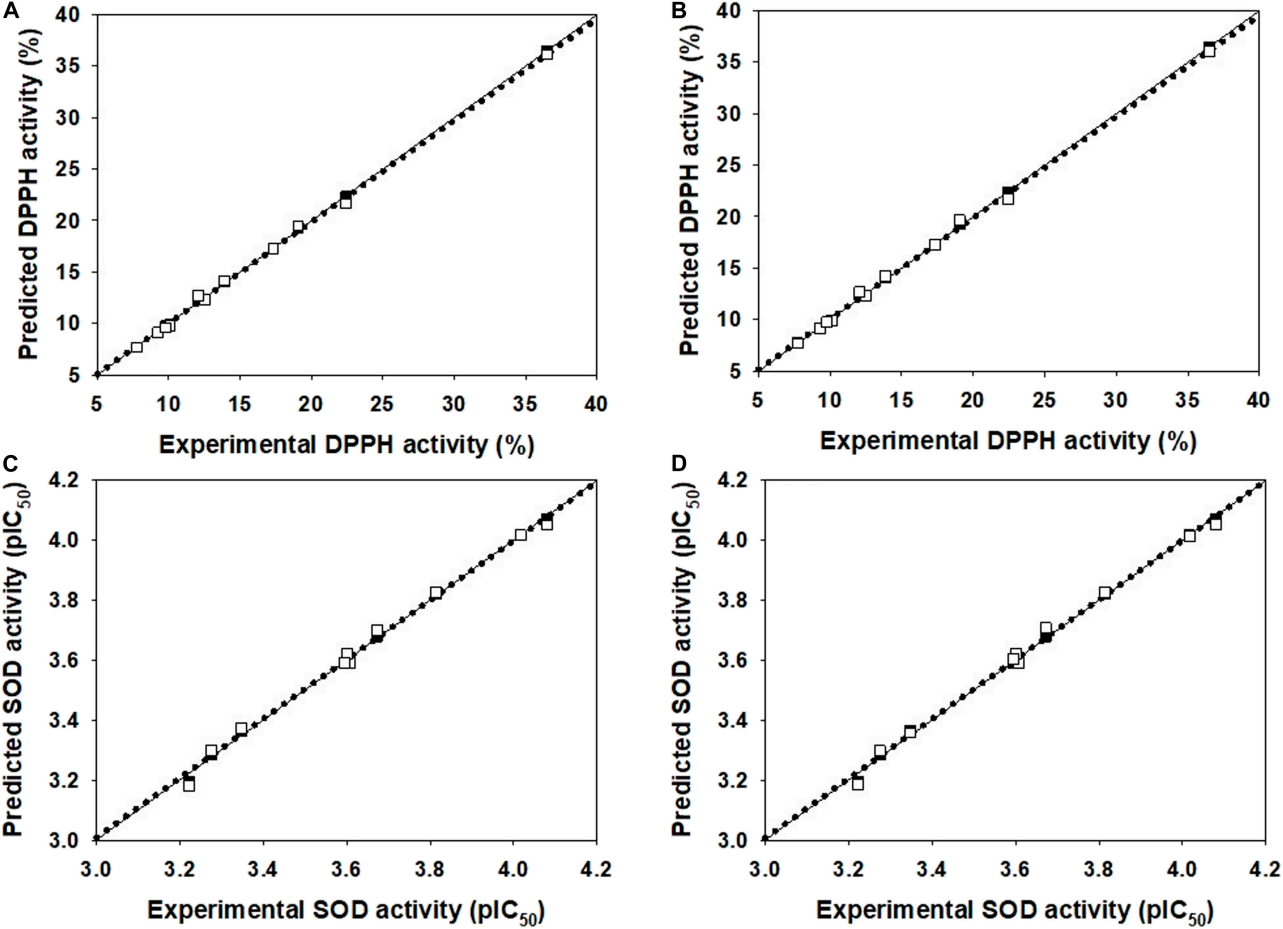
Plots of experimental and predicted activities of the 2,2-diphenyl-1-picrylhydrazyl (DPPH) model for (A) LOO-CV and (B) 5-fold-CV sets and the superoxide dismutase (SOD) model for (C) LOO-CV and (D) 5-fold-CV sets. Training set is represented by black squares and solid lines, while the LOO-CV and 5-fold-CV sets are represented by white squares and dotted lines.

Two QSAR models (DPPH and SOD models) were constructed by MLR algorithms using Weka software, version 3.4.5 (Table 4). The models utilized a training set to generate the QSAR models, which were subsequently validated using LOO-CV and 5-fold-CV sets. It was revealed that electronegativity (GATS1e), signal 24 / unweighted (Mor24u), polarizability (Mor04p), and I-state (ATS5s) were noted as key properties influencing DPPH activity, whereas connectivity (D211), van der Waals volume (R1v), polarizability (AATS8p), and the presence/absence of carbon–oxygen (B08[C–O]) were noted for SOD activity (Tables 3 and 4). Both constructed models provided preferable predictive performance (Table 4) as indicated by high correlation values above 0.99 for training (*R^2^*: 0.9980 to 0.9996) and LOO-CV (*Q^2^*: 0.9930 to 0.9978) as well as low RMSE values (*RMSE_Tr_*: 0.0121 to 0.1677 and *RMSE*_*LOO-CV*_: 0.0233 to 0.3830). In addition, 5-fold-CV demonstrated robust statistical performance (Table 4) as shown by high correlation coefficients and low RMSE values for both training (*R^2^ =* 0.9980 to 0.9996 and *RMSE_Tr_* = 0.0121 to 0.1677) and validation (*Q^2^*_*5-fold-CV*_ = 0.9936 to 0.9978 and *RMSE*_*5-fold-CV*_ = 0.0220 to 0.3997) sets. These statistical values indicated the acceptable reliability of the models (*R^2^* > 0.6 and *Q^2^* > 0.5) [24,50]. To further ensure the reliability and predictive accuracy of the built models, 3 additional statistical parameters were assessed, including *R^2^_adj_*, MAE, and CCC. The DPPH model showed the following results: training set with *R^2^_adj_* = 0.9993 and MAE = 0.1384; LOO-CV set with *R^2^_adj_* = 0.9963, MAE = 0.3147, and CCC = 0.9988; and 5-fold-CV set with *R^2^_adj_* = 0.9963, MAE = 0.3171, and CCC = 0.9987. Similarly, the SOD (pIC_50_) model displayed the following results: training set of *R^2^_adj_ =* 0.9964 and MAE = 0.0099; LOO-CV set of *R^2^_adj_* = 0.9874, MAE = 0.0203, and CCC = 0.9964; and 5-fold-CV set of *R^2^_adj_* = 0.9885, MAE = 0.0194, and CCC = 0.9968. The calculated parameters of both built models (i.e., DPPH and SOD) showed preferable high *R^2^_adj_* and CCC values along with low MAE values for all evaluated sets (i.e., training, LOO-CV, and 5-fold-CV sets), indicating their preferably high predictive performance and robustness (threshold of MAE < 0.6 and CCC > 0.85). The results of the predicted values and comparative plots of experimental versus predicted activities for LOO-CV and 5-fold-CV sets of both models are provided in Supplementary Materials (DPPH model: Table S1 and Fig. 2A and B, and SOD model: Table S22 and Fig. 2C and D). The predictive reliability of the models was also demonstrated by the closeness of the dots displayed in the plots of experimental versus predicted activities (Fig. 2A to D). This was supported by the low calculated residual values (Tables S1 and S2), which is a difference between experimental and predicted values, obtained from LOO-CV and 5-fold-CV sets of both models. Plots of experimental activities versus residual values of both DPPH (Fig. S23A and B) and SOD (Fig. S23C and D) models using LOO-CV and 5-fold-CV sets revealed that most of the dots are distributed near the zero axis, indicating that the models are well performed to provide close values to those of actual ones.

**Table 4.** QSAR equations and statistical parameters indicating predictive performance of the antioxidant models (DPPH and SOD)

Activity	Equation	*N*	Training set		LOO-CV set		5-Fold-CV set	
			*R^2^_Tr_*	*RMSE_Tr_*	*Q^2^*_*LOO-CV*_	*RMSE*_*LOO-CV*_	*Q^2^*_*5-fold-CV*_	*RMSE*_*5-fold-CV*_
DPPH	%DPPH = 0.0685(ATS5s) − 74.5372(GATS1e) − 6.0133(Mor04p) − 12.0745(Mor24u) + 20.4924	11	0.9996	0.1677	0.9978	0.3830	0.9978	0.3997
SOD	pIC_50_ = 7.8664(R1v) − 2.2827(AATS8p) − 0.1794(B08[C–O]) + 22.3828(D211) − 3.3978	10	0.9980	0.0121	0.9930	0.0233	0.9936	0.0220

SOD, superoxide dismutase; DPPH, 2,2-diphenyl-1-picrylhydrazyl; QSAR, quantitative structure–activity relationship; LOO-CV, leave-one-out cross-validation; 5-fold-CV, 5-fold cross-validation; *R^2^_Tr_*, squared correlation coefficient; *RMSE_Tr_*, root mean square error for training; *Q^2^*_*LOO-CV*_, cross-validated *R^2^* for LOO-CV; *RMSE_LOO-CV_*, root mean square error for LOO-CV; *Q^2^*_*5-fold-CV*_, cross-validated *R^2^* for 5-fold-CV; *RMSE*_*5-fold-CV*_, root mean square error for 5-fold-CV; pIC_50_, the negative base 10 logarithm of the SOD IC_50_ (μM)

Furthermore, Y-randomization was performed to verify that the obtained QSAR models were not generated by chance correlation. The dependent variables, DPPH (%) and SOD (pIC₅₀), were randomly shuffled, and new models were generated using the same MLR method. This procedure was repeated 10 times. The *R^2^* and *Q^2^* values of the randomized models were then compared with those of the original models to assess model reliability. All randomized models exhibited lower *R^2^* and *Q^2^* values than the original models, indicating good model robustness and confirming the absence of chance correlation (Supplementary Materials, Fig. S24).

In addition, the applicability domain (AD) of the QSAR models (DPPH and SOD) was evaluated using the leverage method and the Williams plot, which combine descriptor-space leverage values with standardized residuals to detect potential structural outliers (high-leverage compounds) and response outliers (large residual deviations). The critical leverage thresholds were calculated as h* = 1.36 for the DPPH model and h* = 1.50 for the SOD model. Compounds exhibiting leverage values *h* < h* and standardized residuals within the ±3σ interval were considered to fall within the AD. Based on these criteria, all compounds were found within the AD for both models, confirming the robustness and predictive reliability of the QSAR models (Supplementary Materials, Figs. S25 and S26).

**Fig. 3.**
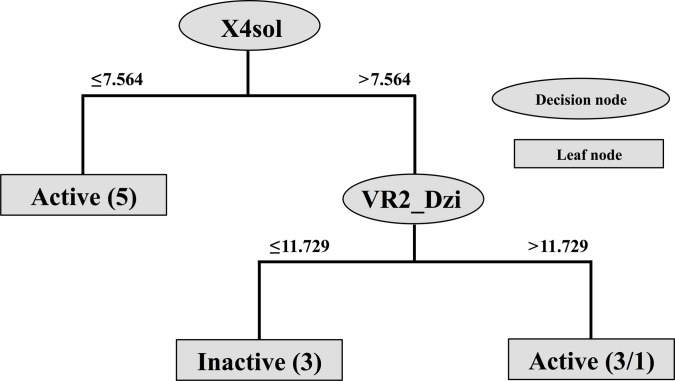
Antimicrobial decision tree classification model of 8-aminoquinoline (8AQ)-based sulfonamides (3-13). The model was constructed using the training set. Decision nodes represent key descriptors used to classify compounds as active or inactive, whereas leaf nodes indicate their antimicrobial classes. The numbers in parentheses in each terminal leaf node indicate the number of correctly classified compounds, followed by the number of incorrectly classified compounds.

Owing to experimental antimicrobial results, the individual tested compounds showed antimicrobial activity in a nonuniform pattern, in which some of them exhibited inhibitory effects against the particular microorganisms. Due to the characteristic of the experimentally obtained data, the classification QSPR model was constructed to elucidate the influencing key structural features (independent variables, *X_n_*) governing the antimicrobial classes (dependent variable, *Y*: active or inactive) of the compounds. Accordingly, an antimicrobial QSPR model was generated using the binary data as the input. Compounds displaying antimicrobial MIC values were assigned as actives, whereas those with no MIC values were assigned as inactives. The QSPR model was built using the decision tree analysis to allow the revealing of an interpretable if-then rule used to classify the compounds. The decision tree is a supervised machine learning algorithm successfully used to generate if-then rules to classify various classes of bioactive compounds (i.e., antimicrobial agents, aromatase inhibitors, and neuraminidase inhibitors) [64–66]. Herein, the decision tree algorithm, namely, J48, implemented in Weka software, version 3.4.5 was used to construct the QSPR model to generate the classification rule representing the descriptor-based cutoffs for categorizing the active and inactive compounds (Fig. 3). Similar to the antioxidant QSAR modeling, the antimicrobial QSPR model was validated using LOO-CV and 5-fold-CV sets. The model was generated using the training set (Fig. 3). It revealed an if-then rule that used 2 decision nodes (X4sol and VR2_Dzi) to identify 3 leaf nodes as antimicrobial classes of the compounds (active or inactive) in a top–down manner. The solvation connectivity index (X4sol: cutoff value of >7.564 or ≤7.564) was used as a root node for initial classification, followed by ionization potential (VR2_Dzi: cutoff value of >11.729 or ≤11.729) as an internal node. It was found that 5 compounds (**3**, **4**, **5**, **6**, and **8**) whose X4sol value are ≤7.564 were initially categorized as actives. The rest of compounds (**7** and **9**-**13**) with X4sol > 7.564 were sequentially categorized using their ionization potential (VR2_Dzi) as inactives (**9**, **11**, and **13** with VR2_Dzi ≤11.729) and actives (**10** and **12** with VR2_Dzi > 11.729). However, an inactive compound **7** (VR2_Dzi > 11.729) was misclassified as an active. Statistical parameters indicated that the constructed QSPR model displayed preferable predictive performance for training and both validation sets as indicated by high values of accuracy (training set = 90.91, LOO-CV set and 5-fold-CV set = 63.64), precision (training set = 0.920, LOO-CV set and 5-fold-CV set = 0.691), recall (training set = 0.909, LOO-CV set and 5-fold-CV set = 0.636), and F-measure (training set = 0.906, LOO-CV set and 5-fold-CV set = 0.642) (Supplementary Materials, Table S7). However, it was observed that the antimicrobial classification model shows a training accuracy of 90.91% and a cross-validated accuracy of 63.64%, indicating a notable drop in performance. The confusion matrices for classifying active and inactive compounds of training, LOO-CV, and 5-fold-CV sets are provided in Supplementary Materials (Table S8). The distribution of antimicrobial classes (as active and inactive) of the tested compounds (**3**-**13**) based on values of their 2 key descriptors (Fig. S27A) also suggested that these descriptor space can effectively classify the compounds.

Using the internal threshold of presenting the MIC values to classify the compound as active, there is a concerning issue regarding the interpretation of the antimicrobial activity of the original dataset used to construct the model. Compound **12** with a high MIC value (765.50 μM or 256 μg/ml) was classified as active, which is considerably weak potency from a drug discovery perspective. Therefore, it is suggested that the built QSPR model is applicable for initial screening, and further experimental investigations are highly required to ensure antimicrobial activities of the compounds.

Although all constructed models (i.e., 2 QSAR antioxidant models and 1 QSPR antimicrobial model) were successfully constructed with acceptable predictive performances, some concerning issues should be noted as their limitations. Furthermore, the datasets used for QSAR/QSPR modeling were considered small-sized datasets because of the limited numbers of experimentally synthesized compounds for generating predictive models (i.e., DPPH = 11 compounds, SOD = 10 compounds, and antimicrobial = 11 compounds). Although the issue of the small-sized datasets is generally concerned for several limitations, it has been demonstrated that the small-sized datasets can be practically used to generate QSAR/QSPR models affording acceptable predictive performance, accuracy, and reliability [17,63,67]. Herein, the main aim of the QSAR modeling is to reveal a set of key structural properties that govern preferable activity of the compounds that are useful for guiding the effective rational design of the new 8AQ derivatives. The activities of the virtually designed compounds can also be predicted for facilitating the selection of potential compounds for further synthesis and validation. To avoid the overfitting issue, we performed the comparative analysis of statistical parameters using the k-fold cross-validation (CV) and LOO-CV testing sets to assess the predictive performance of constructed models. However, the external test sets are not available for further validating the model’s predictive performance outside the training datasets. Therefore, the reliability and accuracy of the constructed QSAR/QSPR models are not perfectly established. In addition, the 3 constructed models are restricted for their generalizability due to the limited size of the datasets.

Another concerning issue was noted regarding the considerably weak DPPH activity of these 8AQ-sulfonamide compounds. The compounds displayed DPPH activity with less than 50%, and their DPPH IC_50_ values were not obtained. The use of percentage values, with a narrow activity window, for modeling may limit statistical reliability, which should be noted as a limitation of the constructed DPPH model. However, the constructed DPPH model aimed for its application in guiding the rational design to obtain the modified compounds with improved radical scavenging potency. The knowledge regarding the structure–activity relationships would be beneficial for the future design of the new 8AQ-based compounds. Regarding the unavoidable narrow activity window of the dataset, the constructed DPPH QSAR model should be noted as a hypothesis-generating model for preliminary prediction rather than predictive in a rigorous quantitative sense.

Furthermore, the notable drop in accuracy of the cross-validated antimicrobial QSPR model (63.64%) compared to its training (90.91%) suggested possible overfitting, which could be due to the small-sized dataset and imbalanced dataset that further limits the interpretability of classification accuracy. Accordingly, the constructed antimicrobial QSPR model is suitable for preliminary classifying the compounds as active or inactive, and further investigations are highly encouraged to confirm their definite antimicrobial properties.

### Rational design

The facilitating role of the QSAR models for guiding the design of new analogs has been demonstrated for various classes of bioactive compounds (i.e., anticancer [27,67], antioxidant [17,68], antimicrobial [69,70], and aromatase-inhibitory [71] agents). Herein, the constructed QSAR/QSPR models were employed for guiding the design of new derivatives based on the key descriptors presented in the models (Table 4 and Fig. 3). Structural modifications were performed by substitutions of electron-withdrawing and electron-donating groups on the quinoline–sulfonamide core of the prototypes to give an additional set of 84 newly designed compounds (i.e., series **3**, **4**, **5**, **6**, **7**, **8**, **9**, **11**, **12**, and **13**) (Table S9 and Figs. S28 to S37). All newly designed compounds were preprocessed to obtain values of key descriptors (Tables S10 to S12) for further predicting their antioxidant activities using the constructed QSAR models (i.e., DPPH and SOD; Table 4) or their antimicrobial classes (active or inactive) using the constructed QSPR decision tree model (Fig. 3).

Predicted antioxidant (i.e., DPPH and SOD) activities and predicted antimicrobial classes of the newly designed compounds are provided in Tables S9 and S12, respectively. The predictions of antioxidant activities indicated that the new analogs performed better predicted activities than their prototypes (i.e., 32 compounds with higher %DPPH and 31 compounds with higher SOD pIC_50_ values; Table S13). These suggested that the structural modification strategies guided by the QSAR models could facilitate efficacious rational design to obtain new compounds with improved activities.

For antimicrobial class prediction, it was found that 34 compounds were classified as actives, whereas 50 compounds were identified as inactives (Tables S12 and S13). The distribution pattern of 84 newly designed compounds in descriptor space indicated that the model can effectively classify the antimicrobial class of the compounds (into active and inactive clusters) based on these 2 key descriptors (X4sol and VR2_Dzi), as shown in Fig. S27B.

Although the reliability of the constructed QSAR/QSPR models used for guiding the design of these new analogs was acceptably ensured, the predicted activities/classes of these virtually designed compounds should be noted as only hypotheses that required further experimental investigations (i.e., synthesis and testing) for validating their potential prior to further development.

### Structure–activity relationship

Experimental antioxidant findings revealed that the tested 8AQ-sulfonamides (**3**-**13**) exhibited more potent SOD activity than the DPPH activity. The result showed that the naphthalene-bearing compound **12** displayed the most potent SOD activity (IC_50_ = 83.34 μM), followed by compounds **8** (X = CN, IC_50_ = 96.07 μM) > **3** (X = F, IC_50_ = 153.56 μM) > **5** (X = Br, IC_50_ = 212.58 μM) > **6** (X = CF_3_, IC_50_ = 247.39 μM). Among others, compound **12** is the only one bearing a naphthalene group. This suggested that the replacement of the sulfonyl benzene ring with the sulfonyl naphthalene ring could enhance the SOD activity of the compounds. When the sulfonyl benzene ring was maintained, the substitution with electron-withdrawing groups such as CN (compound **8**) and the mono-halogen atom (compounds **3** and **5** with X = F and Br, respectively) was suggested for preferable SOD activity. In contrast, substitutions on the benzene ring with the NO_2_ group (*o*-NO_2_ compound **9** with IC_50_ = 254.58 μM and *p*-NO_2_ compound **10** with IC_50_ = 448.75 μM), the tetra-CH_3_ group (compound **11**, IC_50_ = 530.93 μM), and SO_2_CH_3_ (compound **13**, IC_50_ = 600.81 μM) gave the compounds with considerably weaker SOD activities. These findings were supported by information obtained from the constructed SOD QSAR model (Table 4), in which the connectivity index descriptor (D211) was noted as the most significant predictor, followed by van der Waals (R1v), polarizabilities (AATS8p), and the presence/absence of C–O (B08[C–O]), respectively (regression coefficient values: D211 = 22.3828, R1v = 7.8664, AATS8p = −2.2827, and B08[C–O] = −0.1794; Table 4). The model indicated that high positive values of D211 and R1v descriptors are required to obtain potent activity. Considering the descriptor profiles of the tested compounds, the most potent SOD-mimic compound **12** bearing naphthalene possessed high D211 (0.074) and the highest R1v (1.188) values. A similar descriptor profile was observed for the second most potent compound **8** bearing CN group (D211 = 0.075 and R1v = 1.164) (Table S2). In contrast, the least potent compound **13** (X = SO_2_CH_3_, pIC_50_ = 3.221) displayed the lowest R1v (1.060) and low D211 (0.074) along with high B08[C–O] (1) values (Table S2). The influences of the polarizability descriptor (AATS8p) were noticed when comparing the mono-halogen-substituted compounds (**3**, **4**, and **5**). Among these compounds, compound **3** (X = F) displayed the highest SOD activity (pIC_50_: **3** [3.814] > **5** [3.672] > **4** = inactive), which could be due to its lowest AATS8p value (AATS8p: **3** = 1.419, **5** = 1.622, **4** = 1.551) (Table S2). The isomeric effect of NO_2_ substitution on the benzene ring was observed, in which the *o-*NO_2_ substitution could give the compound **9** (X = *o-*NO_2_, IC_50_ = 254.58 μM) with better SOD activity when compared to that of the compound **10** bearing the *p-*NO_2_ (X = *p-*NO_2_, IC_50_ = 448.75 μM). This could be due to the position of the substituted NO_2_ group that affected the values of 2 main descriptors (D211: **9** = 0.077 > **10 =** 0.074 and R1v: **9** = 1.102 > **10 =** 1.083; Table S2). Additionally, it was observed that the replacement of mono-F of compound **3** (pIC_50_ = 3.814) with trifluoromethyl group to give compound **6** (pIC_50_ = 3.607) could affect the values of D211 and R1v descriptors, leading to decreased SOD activity (**3**: D211 = 0.076 and R1v = 1.113, **6:** D211 = 0.074 and R1v = 1.085) (Table S2).

**Fig. 4.**
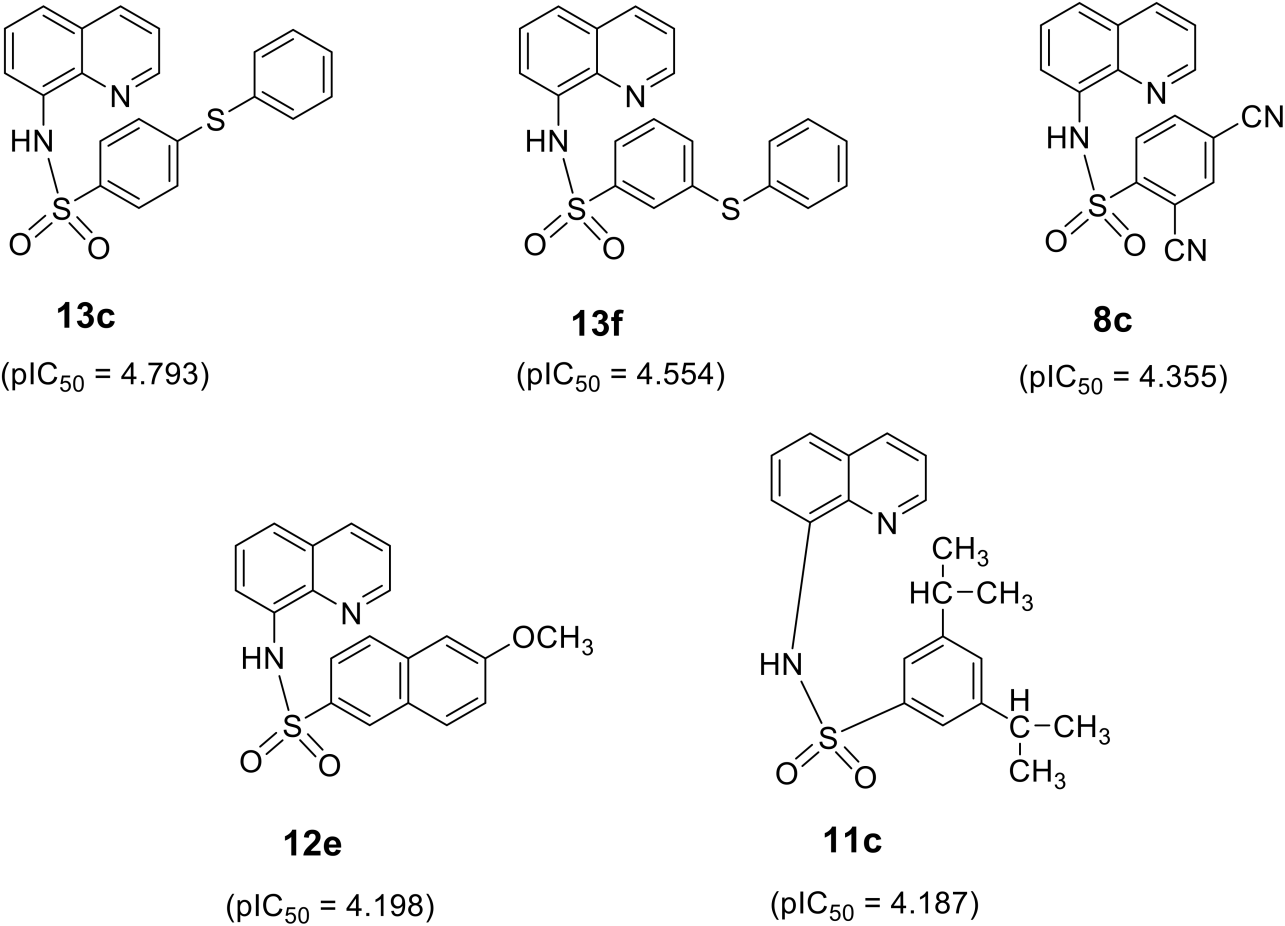
Summary of top 5 newly designed compounds with the highest predicted superoxide dismutase (SOD) activity.

Five most promising newly designed compounds (Fig. 4) with the highest predicted SOD activity were listed as **13c** (pIC_50_ = 4.793) > **13f** (pIC_50_ = 4.554) > **8c** (pIC_50_ = 4.355) > **12e** (pIC_50_ = 4.198) > **11c** (pIC_50_ = 4.187) (Table S9). It should be noted that a replacement of sulfone (SO_2_CH_3_) group on prototype **13** (experimental pIC_50_ = 3.221, R1v = 1.060, and D211 = 0.074; Table S2) with a sulfide-linked benzene ring could considerably improve the activities of the compounds as observed for **13c** (predicted pIC_50_ = 4.793, X = *p-*SC_6_H_5_) and **13f** (predicted pIC_50_ = 4.554, X = *m-*SC_6_H_5_). The enhancing SOD activity of these 2 derivatives (**13c** and **13f**) could be due to the increasing values of 2 influential descriptors including R1v (**13c** = 1.305 and **13f** = 1.262) and D211 (**13c** = 0.077 and **13f** = 0.078) (Table S11). Substitution with another *o*-CN group on the core of *p*-CN prototype **8** (experimental pIC_50_ = 4.017) to give *o*-, *p*-di-CN compound **8c** with improved SOD activity (predicted pIC_50_ = 4.355) (Table S9). Similarly, a substitution with OCH_3_ on the naphthalene ring of prototype **12** (experimental pIC_50_ = 4.079) could give compound **12e** with more potent activity (predicted pIC_50_ = 4.198).

Experimental DPPH assay indicated that none of the tested 8AQ-sulfonamides displayed DPPH activity greater than 50%. Compound **9** (X = *o-*NO_2_) displayed the highest DPPH activity (%DPPH = 36.49%), followed by compound **11** (X = tetra-CH_3_, %DPPH = 22.43%), compound **10** (X = *p-*NO_2_, %DPPH = 19.06%), and compound **13** (X = SO_2_CH_3_, %DPPH = 17.33%) (Table 1). It was noticed that the effects of substitutions on the DPPH activity of this set of 8AQ-sulfonamides are conversely observed from those affecting the SOD activity. Compounds that exhibited preferable SOD IC_50_ values (i.e., compounds **12**, **8**, **3**, and **5**) were ranked as the least potent DPPH compounds, displaying considerably low %DPPH (**12** = 13.88%, **8** = 12.53%, **3** = 10.09%, and **5** = 7.74%; Table 1). From the DPPH QSAR model (Table 4), the electronegativity descriptor GATS1e was noted as the most influential predictor, as indicated by its highest regression coefficient value (−74.5372), followed by Mor24u (−12.0745), Mor04p (−6.0133), and ATS5s (0.0685), respectively. To obtain a high %DPPH value, a compound needs low positive GATS1e but high positive ATS5s values. Considering the descriptor profile of the most potent DPPH compound **9** (X = *o-*NO_2_, %DPPH = 36.49%), the compound displayed the lowest positive GATS1e (0.345) but the highest positive ATS5s (560.981) values, which were aligned with its highest potent experimental DPPH activity. In contrast, the least potent compound **5** (%DPPH = 7.74%) exhibited high positive GATS1e (0.457) but the lowest positive ATS5s (266.058) values (Table S1), supporting its low experimentally observed DPPH activity. Moreover, the enhancing effect of the *o-*NO_2_ substitution on the benzene ring (**9**: X = *o-*NO_2_, %DPPH = 36.49%; Table 1) on DPPH activity compared to the *p-*NO_2_ substitution (**10**: X = *p-*NO_2_, %DPPH = 19.06%; Table 1) was noticed similarly to that found for the SOD activity. The descriptor profiles of these 2 compounds (**9** and **10**) showed that the position of NO_2_ substitution affected values of all key descriptors, except for the GATS1e (Table S1). The *o-*NO_2_ substitution provided the better descriptor profile (i.e., higher positive ATS5s and Mor24u values and higher negative Mor04p value) that supports the achievement of the higher %DPPH when compared to compound **10** with the *o-*NO_2_ substitution (Table S1).

**Fig. 5.**
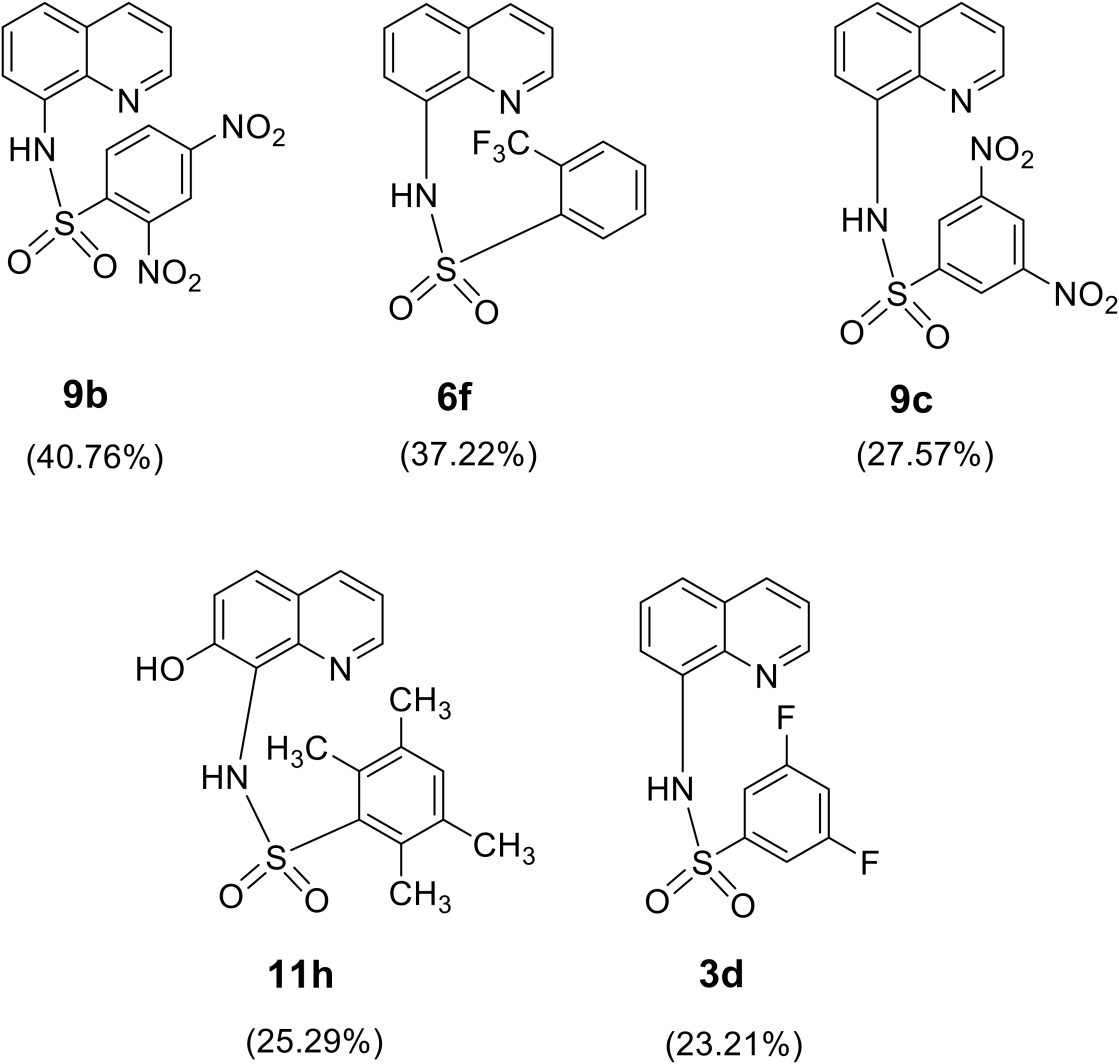
Summary of top 5 newly designed compounds with the highest predicted 2,2-diphenyl-1-picrylhydrazyl (DPPH) activity.

The top 5 structurally modified compounds (Fig. 5) with the most potent predicted %DPPH were ranked as **9b** (40.76%) > **6f** (37.22%) > **9c** (27.57%) > **11h** (25.29%) > **3d** (23.21%), as shown in Table S9. The high predicted %DPPH of compounds **9b** (X = *o-* and *p-*NO_2_ groups) and **9c** (X = di *m-*NO_2_ groups) suggested that the presence of di-NO_2_ in the molecule is essential for radical scavenging effect of the compounds, and the substituted position could affect DPPH activity of the compounds. The effect of the substitution position was also noted for compound **6f** (X = *o*-CF_3_), which displayed improved predicted activity compared to its parent compound **6** (X = *p*-CF_3_, experimental %DPPH = 9.79%; Table 1). It was also noted that an addition of the OH group on the 8AQ ring to give compound **11h** could improve %DPPH (**11**: experimental %DPPH = 22.43%; Table 1). Although the improved DPPH activities of the modified compounds were achieved, it should be noted that the predicted activities of these newly designed compounds are still inferior to desirable effects (as indicated by predicted %DPPH less than 50%).

The constructed QSPR decision tree model (Fig. 3), performed well to correctly identify antimicrobial classes of 10 out of 11 compounds in the training set and 7 out of 11 compounds in the LOO-CV and 5-fold-CV sets, suggested that the antimicrobial activity of the studied compounds is classified orderly based on 2 main properties (i.e., solvation connectivity index [X4sol] and ionization potential [VR2_Dzi]). These findings suggested that different types of functional groups substituted on the 8AQ-sulfonamide core could affect the key properties (i.e., solvation and ionization potential) that govern the antimicrobial effects of the compounds.

Thirty-four structurally modified compounds exhibiting antimicrobial activity are presented in Supplementary Materials (Fig. S38). From the newly designed compounds, it was suggested that the types and number of the halogen atoms (i.e., F, Cl, Br, and I) substituted on the core of the prototypes affected the antimicrobial activities of their modified analogs. The enhancing effects of fluorine substitutions were observed for modified compounds of series **3**, in which the compounds bearing at least 1 F atom (i.e., mono-F compounds **3a**-**3b** or di-F compounds **3c**-**3d**) were predicted to be active antimicrobial agents. In contrast, the presence of at least a Cl, I, or Br atom seemed to diminish the antimicrobial effects as shown by compounds in series **4** and **5**, except for the di-Cl-F compounds (i.e., **4g** and **4h** with *p-*Cl substitution on the sulfonyl benzene ring) (Table S12 and Figs. S28 to S30 and S38). This may imply that halogen substitutions play crucial roles in modulating antimicrobial effects of the compounds.

Previous related works have reported antioxidant and antimicrobial activities of the 8AQ-based compounds, and their redox potential was noted to be one of the influential key properties linked to their biological activities [72]. In overview, structural modifications with several types of functional moieties could give new compounds with improved or worsen predicted activities. This could be due to electron-donating and electron-withdrawing effects of the substituents that interfere redox behavior of the compounds.

## Conclusion

The discovery of new classes of antioxidant and antimicrobial agents has gained attention to address current health issues. 8AQ is a pharmacophore well recognized for its antimalarial effect, making it a promising core for the design of hybrid compounds for therapeutic applications. Herein, a set of 11 newly synthesized 8AQ-based sulfonamides (**3**-**13**) were experimentally investigated for their antioxidant and antimicrobial activities. These compounds exhibited notable SOD-mimic activity but weak DPPH radical scavenging activity. The naphthalene-bearing compound **12** was noted as the most potent antioxidant agent. Antimicrobial investigation indicated that only 7 compounds (i.e., halogen-containing compounds [**3**, **4**, **5**, and **6**], nitrile compound [**8**], *p-*NO_2_ compound **10**, and naphthalene-containing compound **12**) were active antimicrobial agents. Particularly, some of them are active anti-MRSA agents. Two antioxidant QSAR (i.e., SOD and DPPH) models and 1 antimicrobial QSPR model were successfully constructed and validated to ensure their preferable predictive performances. The constructed models were further used to guide the design and predict antioxidant activities or antimicrobial classes of an additional set of 84 newly designed compounds, in which their antioxidant activities or antimicrobial class were predicted using the constructed models. Most of the newly designed compounds display better predicted antioxidant activities when compared to their prototypes, suggesting the effectiveness of the constructed models in guiding the design. A series of 5 compounds with the most preferable predicted antioxidant activities (SOD: **13c**, **13f**, **8c**, **12e**, and **11c**; DPPH: **9b**, **6f**, **9c**, **11h**, and **3d**) and 34 compounds with active predicted antimicrobial class were suggested to be further developed. Substitutions on the 8AQ-sulfonamide core with naphthalene, halogen atoms (i.e., F and Cl), NO_2_, CN, and SC_6_H_5_ groups were revealed to improve activities of the modified compounds. In summary, the study demonstrates the role of computational modeling in guiding the efficacious design of new 8AQ-based sulfonamides. To validate the predictions, these highlighted newly designed compounds should be further synthesized and experimentally investigated for their activities. Further studies (i.e., in vitro and in vivo) regarding their pharmacokinetics (absorption, distribution, metabolism, and excretion), pharmacodynamics (molecular docking), toxicity profiles, and mechanism of action are highly encouraged for successful development.

## Data Availability

The data supporting the findings of this study are available from the corresponding authors upon request.
