# Supplementary material for: Synthesis, Biological Studies, and In Silico-Driven Design of 8-Aminoquinoline-Based Sulfonamide Derivatives as Potential Antioxidant and Antimicrobial Agents
Source: Comput Struct Biotechnol J. 2026 Apr 17;35(1):0032. doi: 10.34133/csbj.0032 (PMC13087402; doi:10.34133/csbj.0032)
Supplement: Supplementary 1 — Figs. S1 to S38 Tables S1 to S13 [file csbj.0032.f1.docx]

**Synthesis, biological studies, and *in silico*-driven design of 8-aminoquinoline-based sulfonamide derivatives as potential antioxidant and antimicrobial agents**

**Ratchanok Pingaew^1,^*, Apilak Worachartcheewan^2,^*, Veda Prachayasittikul^3^,**

**Rungrot Cherdtrakulkiat^4^, Supaluk Prachayasittikul^3^, Somsak Ruchirawat^5,6,7^,**

**Virapong Prachayasittikul^4^**

*^1^Department of Chemistry, Faculty of Science, Srinakharinwirot University, Bangkok 10110, Thailand*

*^2^Department of Community Medical Technology, Faculty of Medical Technology, Mahidol University, Bangkok 10700, Thailand*

*^3^Center for Research Innovation and Biomedical Informatics, Faculty of Medical Technology, Mahidol University, Bangkok, 10700, Thailand*

*^4^Department of Clinical Microbiology and Applied Technology, Faculty of Medical Technology, Mahidol University, Bangkok 10700, Thailand*

*^5^Laboratory of Medicinal Chemistry, Chulabhorn Research Institute, Bangkok, 10210, Thailand*

*^6^Program in Chemical Sciences, Chulabhorn Graduate Institute, Bangkok, 10210, Thailand*

*^7^Center of Excellence on Environmental Health and Toxicology (EHT), Commission on Higher Education, Ministry of Education, Bangkok, 10400, Thailand*

* Corresponding authors. E-mail addresses: ratchanok@g.swu.ac.th (R. Pingaew), [apilak.woa@mahidol.ac.th](mailto:apilak.woa@mahidol.ac.th) (A. Worachartcheewan)

**Content**

**Page**

**Fig. S1.** ^1^H NMR spectrum (300 MHz, DMSO-d_6_) of compound **3** 5

**Fig. S2**. ^13^C NMR spectrum (75 MHz, DMSO-d_6_) of compound **3** 5

**Fig. S3.** ^1^H NMR spectrum (300 MHz, DMSO-d_6_) of compound **4** 6

**Fig. S4.** ^13^C NMR spectrum (75 MHz, DMSO-d_6_) of compound **4** 6

**Fig. S5.** ^1^H NMR spectrum (500 MHz, DMSO-d_6_) of compound **5** 7

**Fig. S6.** ^13^C NMR spectrum (125 MHz, DMSO-d_6_) of compound **5** 7

**Fig. S7.** ^1^H NMR spectrum (500 MHz, DMSO-d_6_) of compound **6** 8

**Fig. S8.** ^13^C NMR spectrum (125 MHz, DMSO-d_6_) of compound **6** 8

**Fig. S9.** ^1^H NMR spectrum (300 MHz, DMSO-d_6_) of compound **7** 9

**Fig. S10.** ^13^C NMR spectrum (75 MHz, DMSO-d_6_) of compound **7** 9

**Fig. S11.** ^1^H NMR spectrum (300 MHz, DMSO-d_6_) of compound **8** 10

**Fig. S12.** ^13^C NMR spectrum (75 MHz, DMSO-d_6_) of compound **8** 10

**Fig. S13.** ^1H^ NMR spectrum (500 MHz, DMSO-d_6_) of compound **9** 11

**Fig. S14.** ^13^C NMR spectrum (125 MHz, DMSO-d_6_) of compound **9** 11

**Fig. S15.** ^1^H NMR spectrum (300 MHz, DMSO-d_6_) of compound **10** 12

**Fig. S16.** ^13^C NMR spectrum (75 MHz, DMSO-d_6_) of compound **10** 12

**Fig. S17.** ^1^H NMR spectrum (300 MHz, DMSO-d_6_) of compound **11** 13

**Fig. S18.** ^13^C NMR spectrum (75 MHz, DMSO-d_6_) of compound **11** 13

**Fig. S19.** ^1^H NMR spectrum (500 MHz, DMSO-d_6_) of compound **12** 14

**Fig. S20.** ^13^C NMR spectrum (125 MHz, DMSO-d_6_) of compound **12** 14

**Fig. S21.** ^1^H NMR spectrum (300 MHz, DMSO-d_6_) of compound **13** 15

**Fig. S22.** ^13^C NMR spectrum (75 MHz, DMSO-d_6_) of compound **13** 15

**Table S1.** Molecular descriptors, experimental and predicted (%DPPH) activity of original 8AQ-based sulfonamides (**3** - **13)** 16

**Table S2.** Molecular descriptors, experimental and predicted (SOD, pIC_50_) activity of original 8AQ-based sulfonamides (**3** - **13**) 17

**Table S3.** Molecular descriptors and antimicrobial activity of original 8AQ-based

sulfonamides (**3** - **13**) 18

**Table S4.** Intercorrelation of key descriptors of the DPPH QSAR model 19

**Table S5.** Intercorrelation of key descriptors of the SOD QSAR model 19

**Table S6.** Intercorrelation of key descriptors of the decision tree QSPR model 19

**Fig. S23.** The distributions of experimental activities and residual values (differences between experimental and predicted activities) for the DPPH model of LOO‑CV (a) and 5‑fold‑CV (b), and for the SOD model of LOO‑CV (c) and 5‑fold‑CV (d). Training data are represented by black squares, while the LOO‑CV and 5‑fold‑CV sets are represented by white squares 20

**Content (Continued)**

**Page**

**Fig. S24.** Y-randomization test results for the QSAR models of DPPH activity for

(a) LOO‑CV and (b) 5‑fold CV sets, and SOD activity for (c) LOO‑CV and

(d) 5‑fold CV sets. The R2 and Q2 values of the original model (red points)

are significantly higher than those of the 10 randomized models (grey points). 21

**Fig. S25.** Williams plot of standardized residuals versus leverage values for

the QSAR model (DPPH activity: LOO-CV (a) and 5-fold CV (b) sets).

The horizontal red dashed lines represent the limit of ± 3 units of

standardized residuals, and the vertical green dashed line represents

the warning leverage (h* = 1.36). Green cycle symbol represented

the tested compounds. Williams plot was performed using

Google Colab with Python code. 22

**Fig. S26.** Williams plot of standardized residuals versus leverage values for

the QSAR model (SOD activity: LOO-CV (a) and 5-fold CV (b) sets).

The horizontal red dashed lines represent the limit of ± 3 units of

standardized residuals, and the vertical green dashed line represents

the warning leverage (h* = 1.50). Green cycle symbol represented

the tested compounds. Williams plot was performed using Google Colab

with Python code. 22

**Table S7.** Statistical results of QSPR model for antimicrobial activity 23

**Table S8.** Confusion matrix for the classification of active and inactive antimicrobial compounds using decision tree analysis 23

**Fig. S27.** Distribution of antimicrobial classes (i.e., active as blue cycles and inactive as red cycles) of original 8AQ-based sulfonamides (**3** - **13**) (a) and 84 newly designed compounds (b) using decision tree model based on two key molecular descriptors 23

**Table S9.** Modification strategies, chemical structures and predicted antioxidant activities (DPPH and SOD) of 84 newly designed 8AQ-based sulfonamide derivatives 24

**Fig. S28.** Chemical structures of modified derivatives of prototype **3** 32

**Fig. S29.** Chemical structures of modified derivatives of prototype **4** 32

**Fig. S30.** Chemical structures of modified derivatives of prototype **5** 33

**Fig. S31.** Chemical structures of modified derivatives of prototype **6** 33

**Fig. S32.** Chemical structures of modified derivatives of prototype **7** 34

**Fig. S33.** Chemical structures of modified derivatives of prototype **8** 35

**Fig. S34.** Chemical structures of modified derivatives of prototype **9** 35

**Fig. S35.** Chemical structures of modified derivatives of prototype **11** 36

**Fig. S36.** Chemical structures of modified derivatives of prototype **12** 36

**Fig. S37.** Chemical structures of modified derivatives of prototype **13** 37

**Table S10.** Numerical values of molecular descriptors of 84 newly designed compounds for predicting DPPH (%) activity 38

**Table S11**. Numerical values of molecular descriptors of 84 newly designed compounds for predicting SOD (pIC_50_) activity 41

**Table S12.** Numerical values of molecular descriptors and predicted antimicrobial activity of 84 newly designed 8AQ-based sulfonamide derivatives 44

**Table S13.** Summarized number of newly designed compounds with improved/decreased predicted activities compared to their prototypes 47

**Fig. S38.** Summary of 34 newly designed compounds exhibiting antimicrobial activity 51


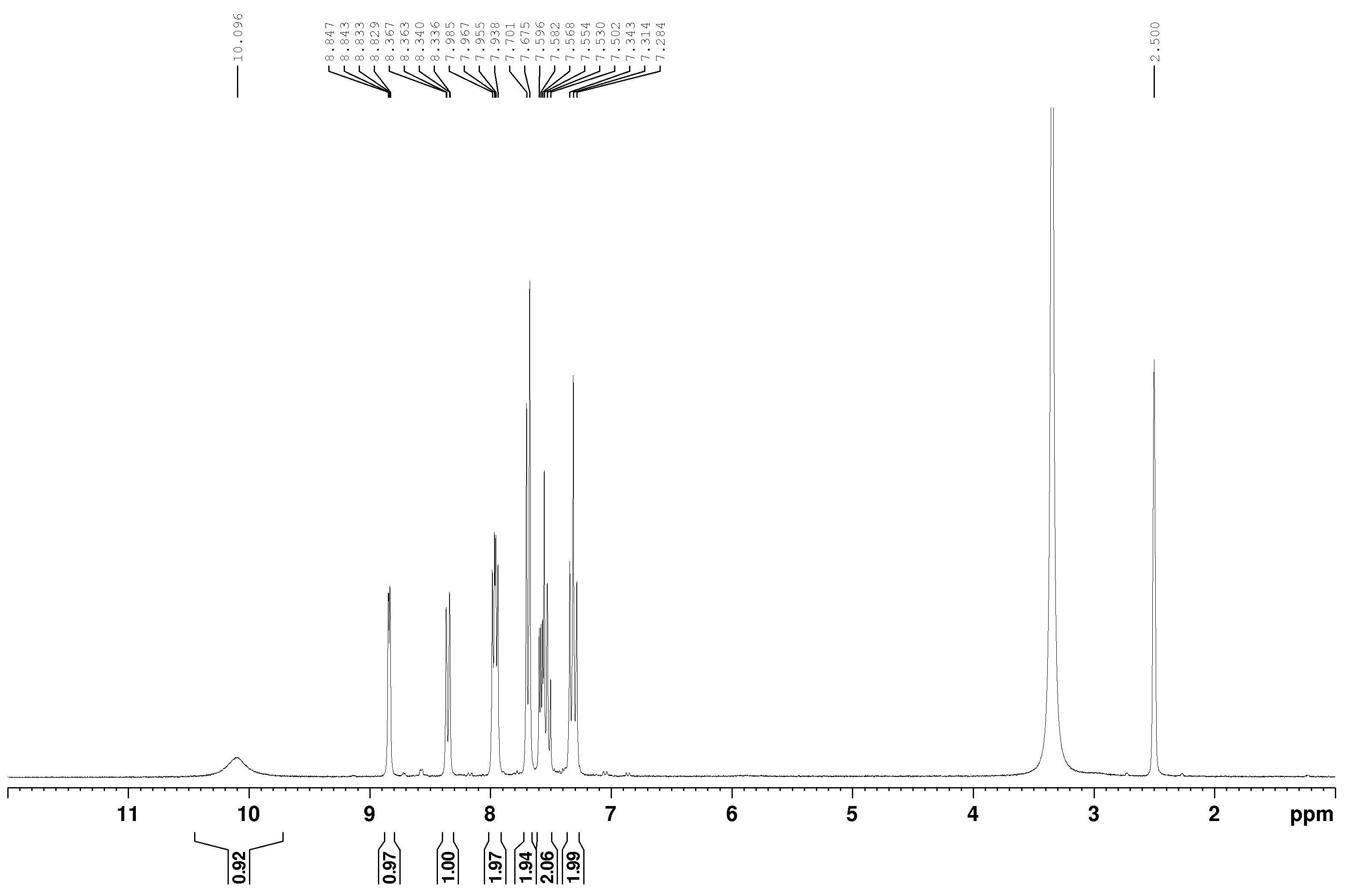


**Fig. S1**. ^1^H NMR spectrum (300 MHz, DMSO-d_6_) of compound **3**.


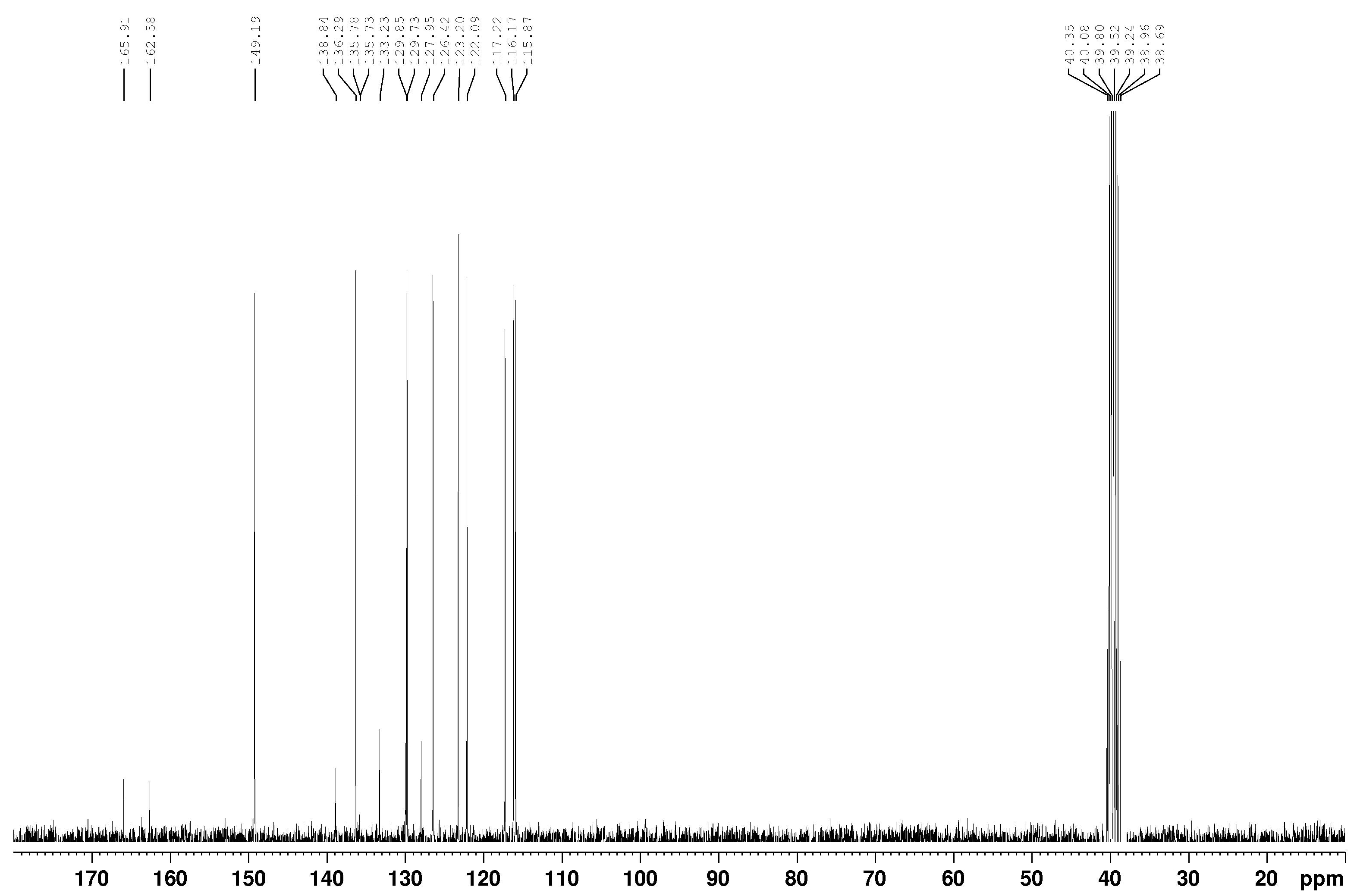


**Fig. S2**. ^13^C NMR spectrum (75 MHz, DMSO-d_6_) of compound **3**.


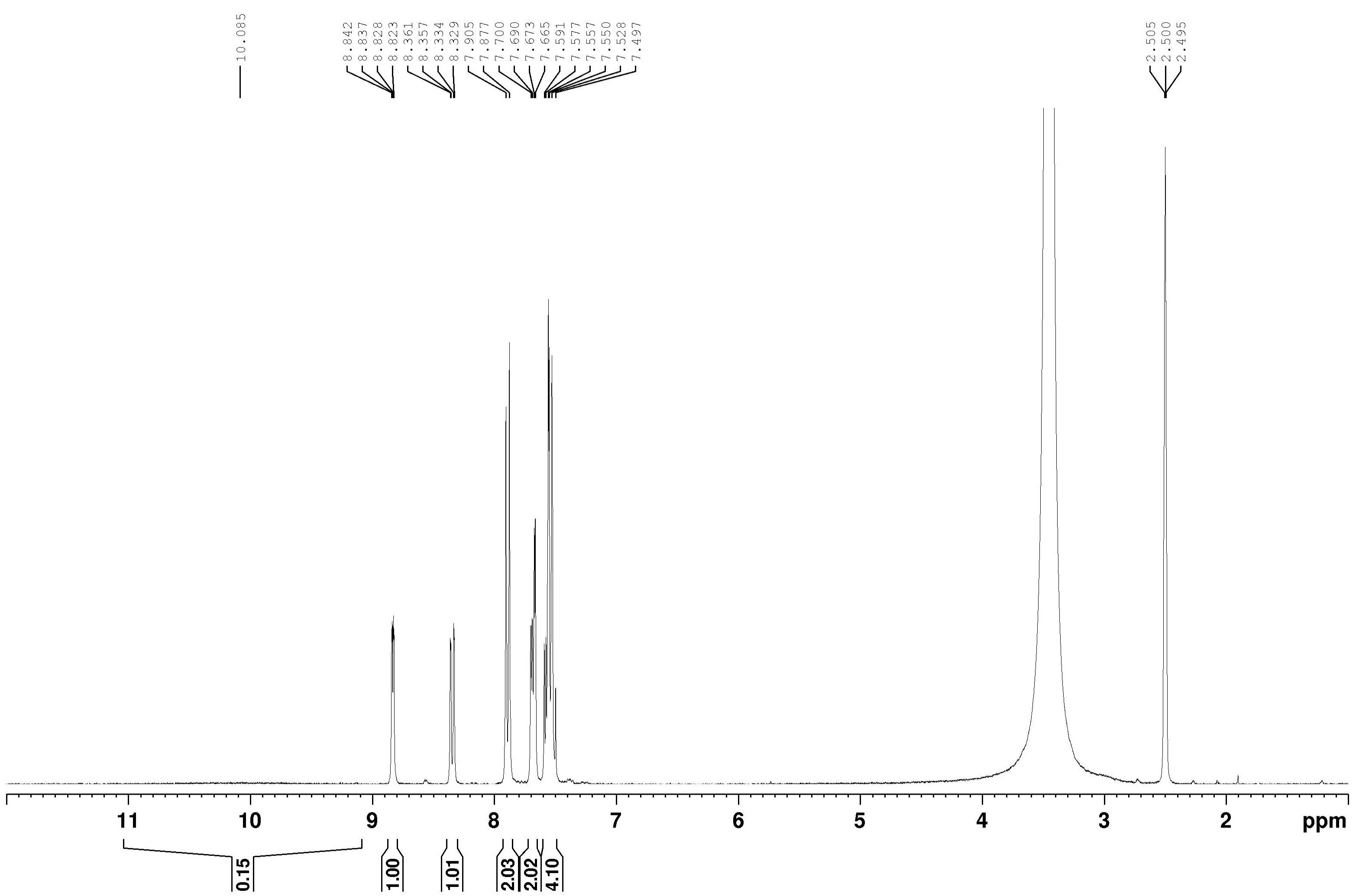


**Fig. S3**. ^1^H NMR spectrum (300 MHz, DMSO-d_6_) of compound **4**.


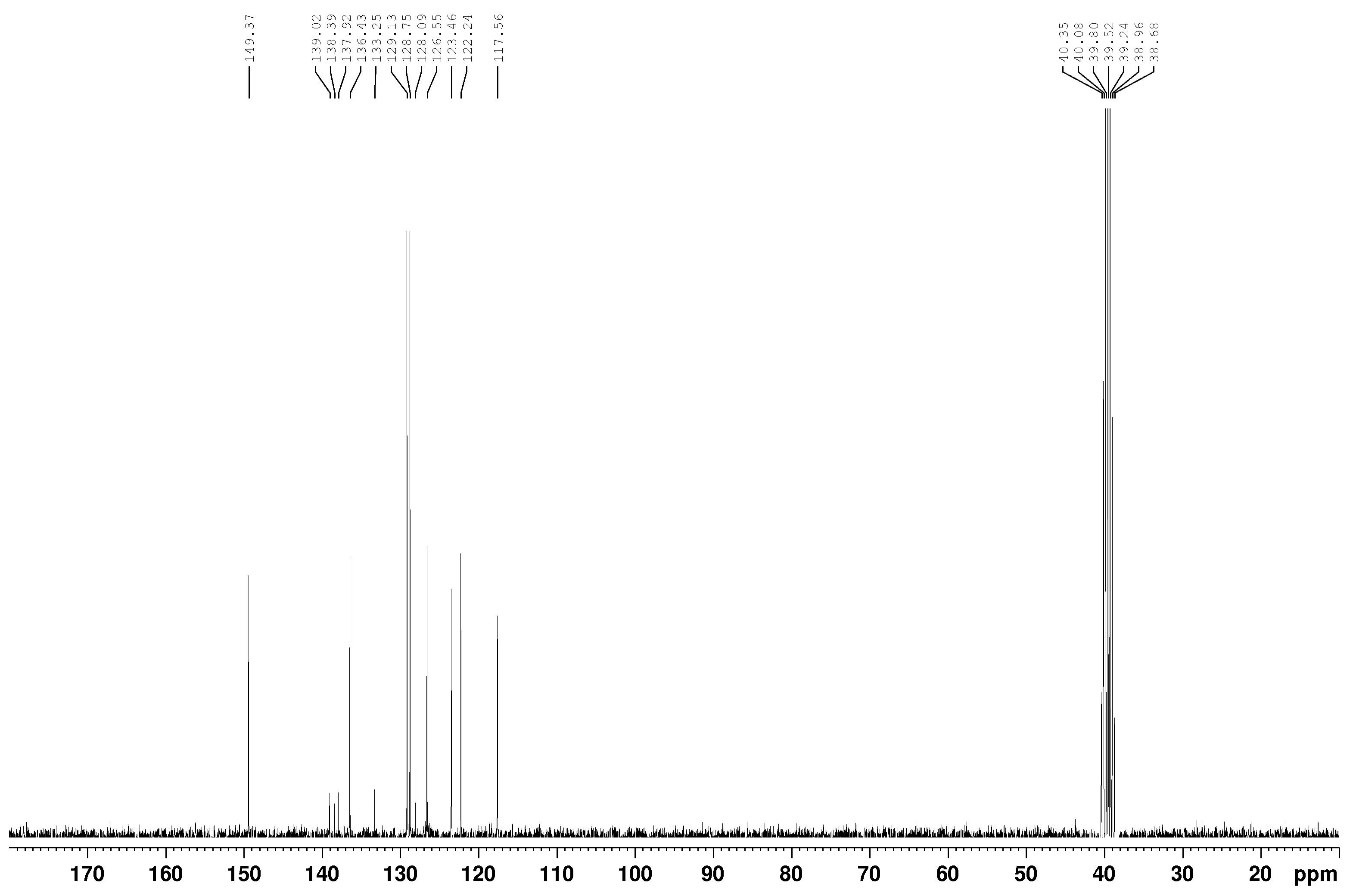


**Fig. S4**. ^13^C NMR spectrum (75 MHz, DMSO-d_6_) of compound **4**.


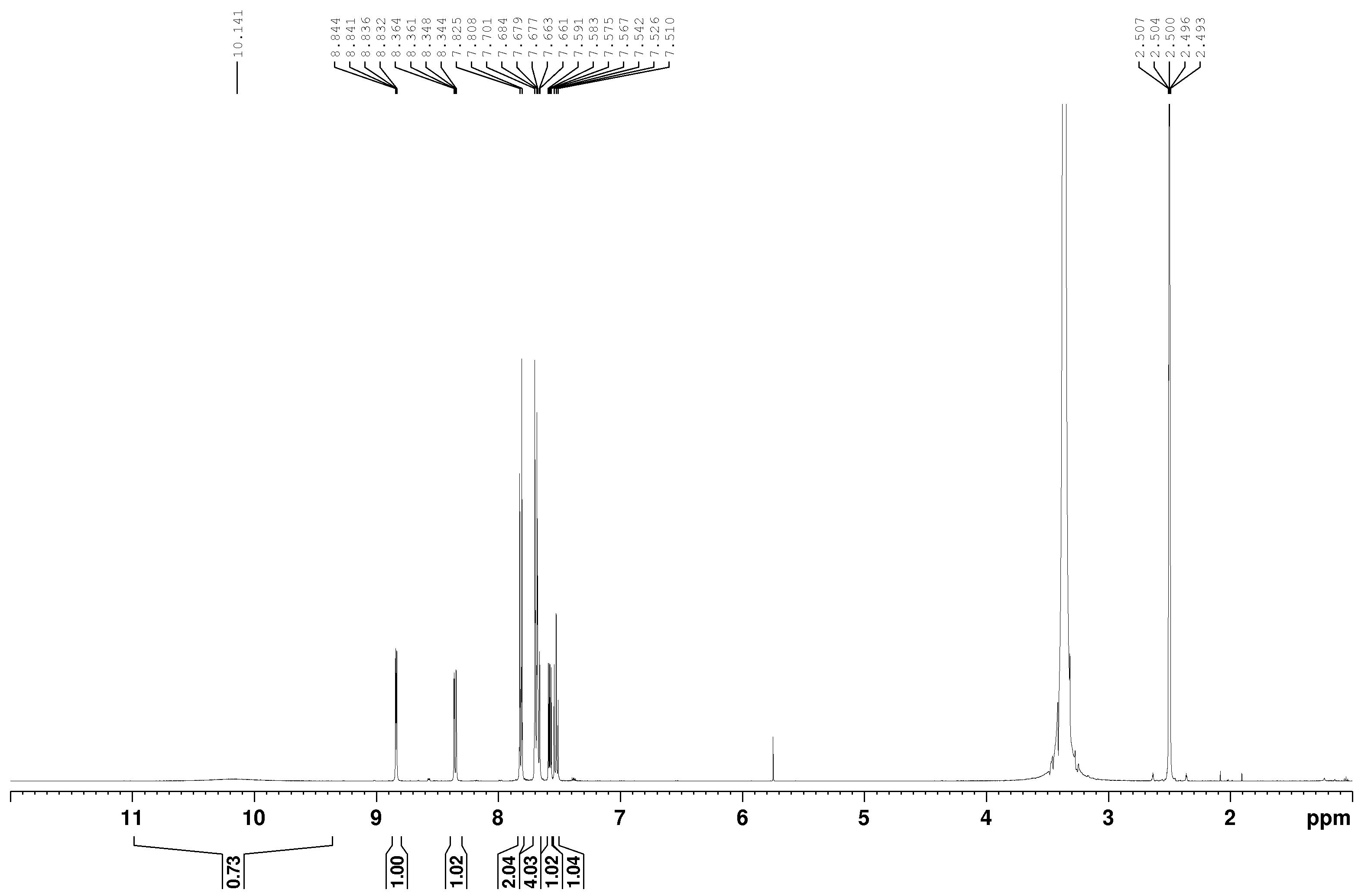


**Fig. S5**. ^1^H NMR spectrum (500 MHz, DMSO-d_6_) of compound **5**.


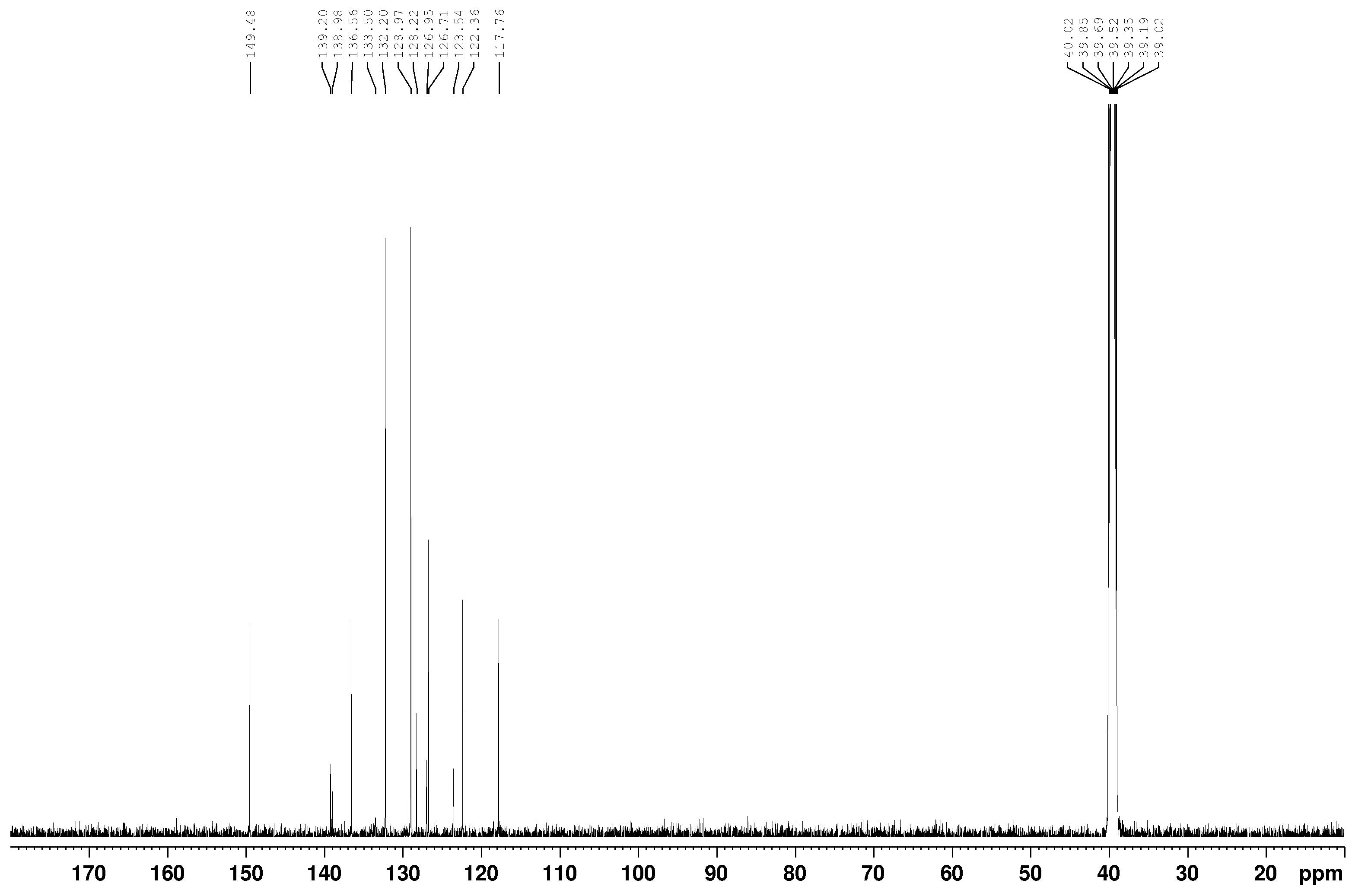


**Fig. S6**. ^13^C NMR spectrum (125 MHz, DMSO-d_6_) of compound **5**.


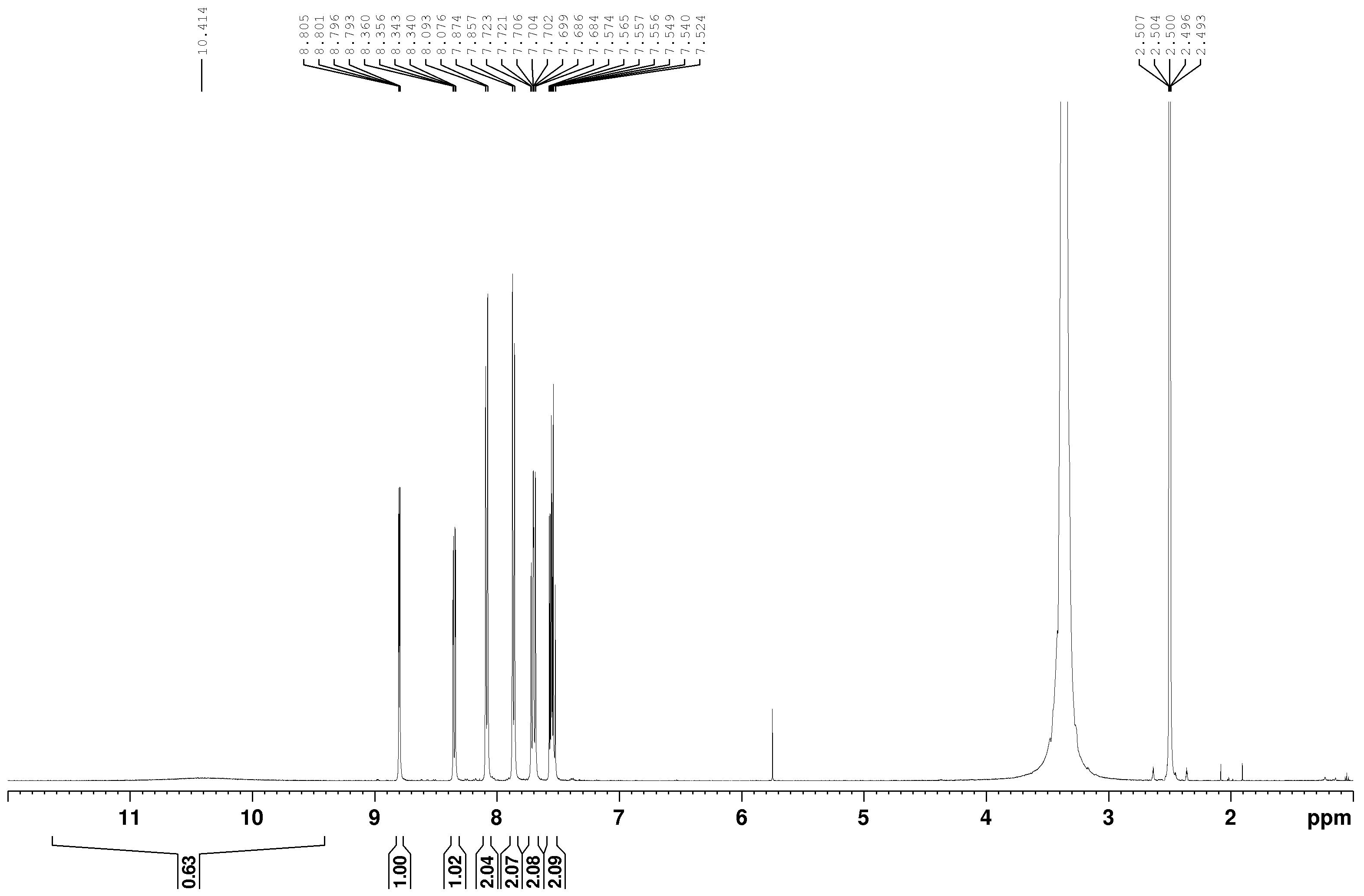


**Fig. S7**. ^1^H NMR spectrum (500 MHz, DMSO-d_6_) of compound **6**.


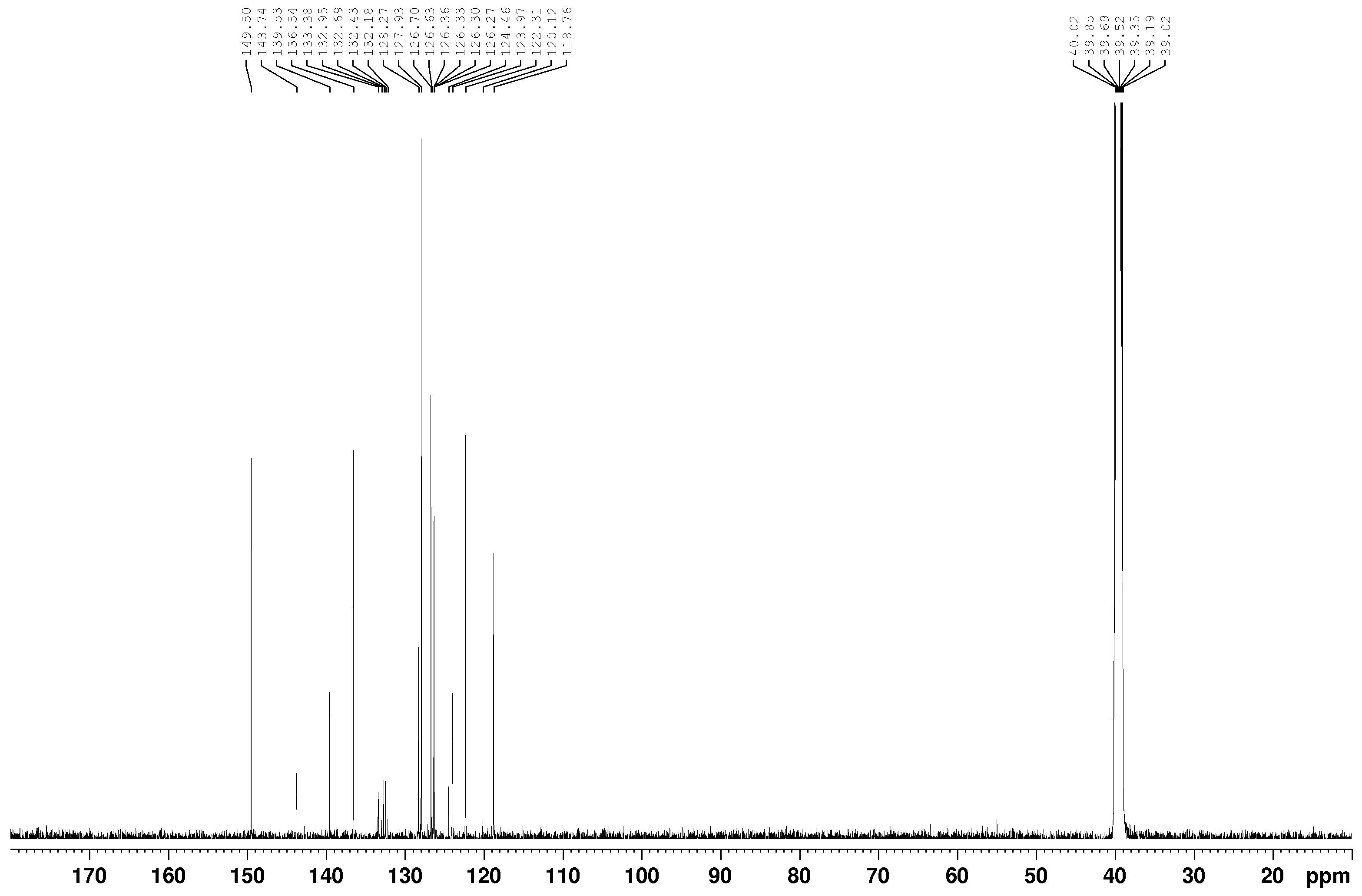


**Fig. S8**. ^13^C NMR spectrum (125 MHz, DMSO-d_6_) of compound **6**.


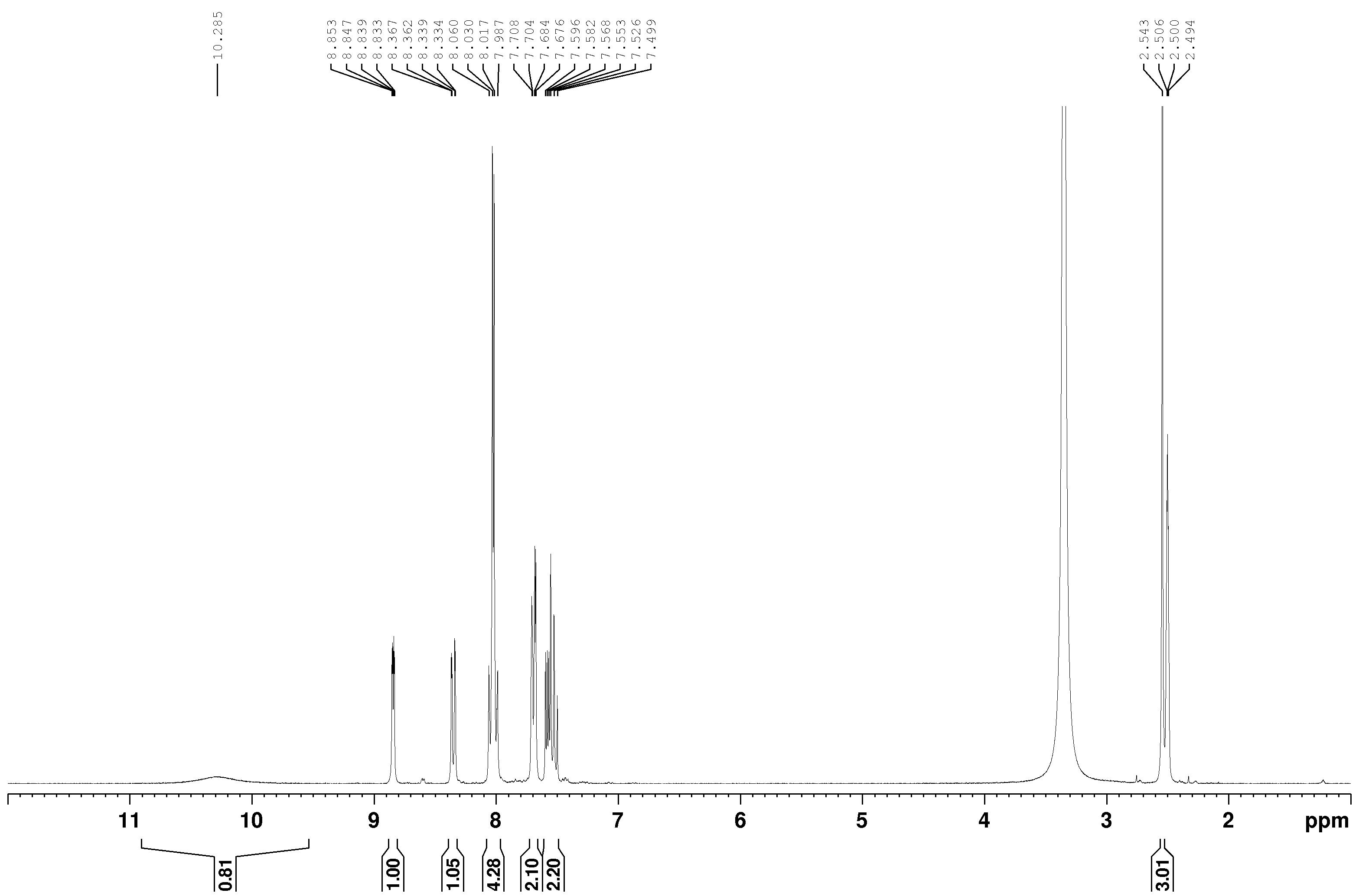


**Fig. S9**. ^1^H NMR spectrum (300 MHz, DMSO-d_6_) of compound **7**.


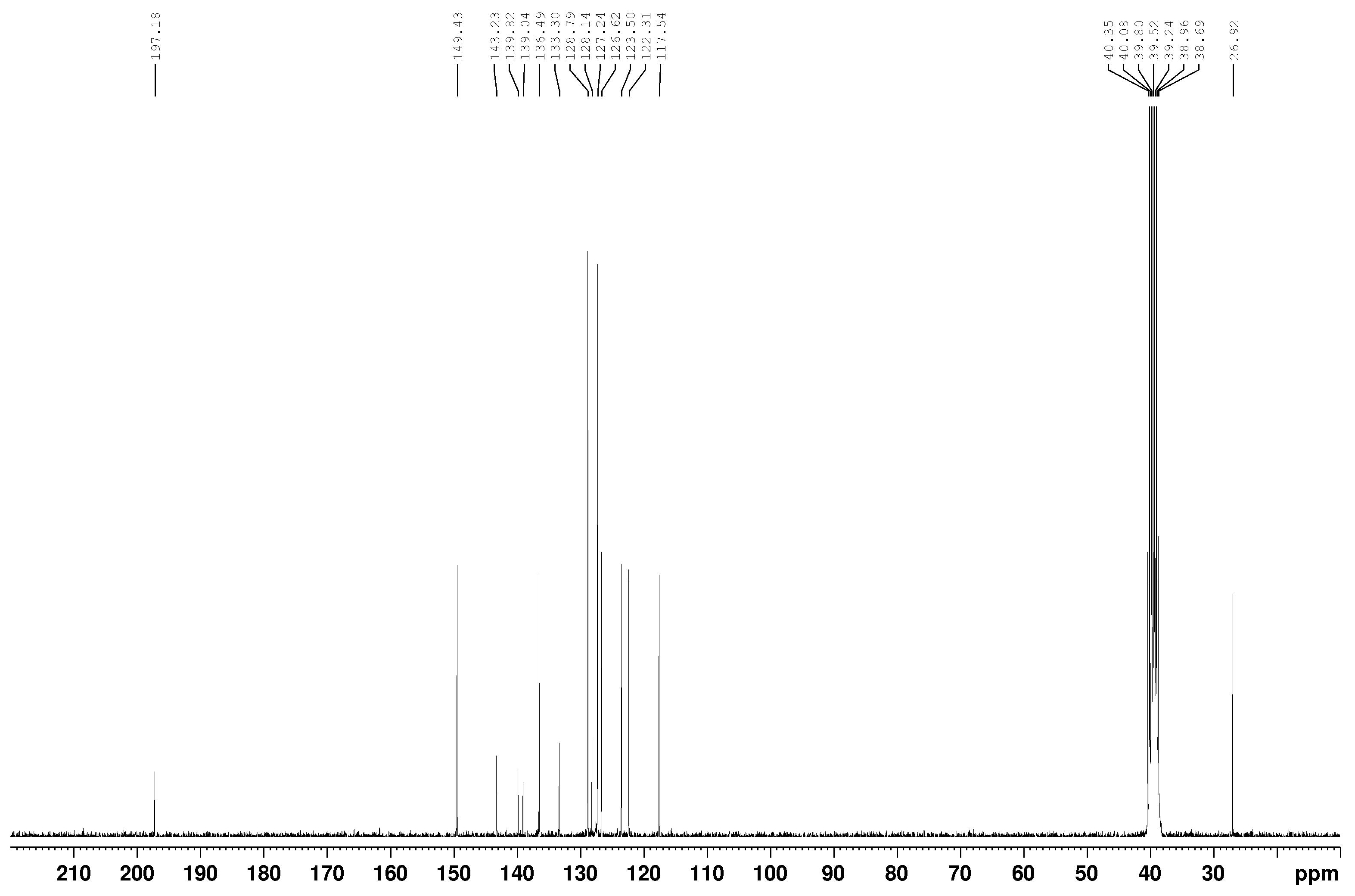


**Fig. S10**. ^13^C NMR spectrum (75 MHz, DMSO-d_6_) of compound **7**.


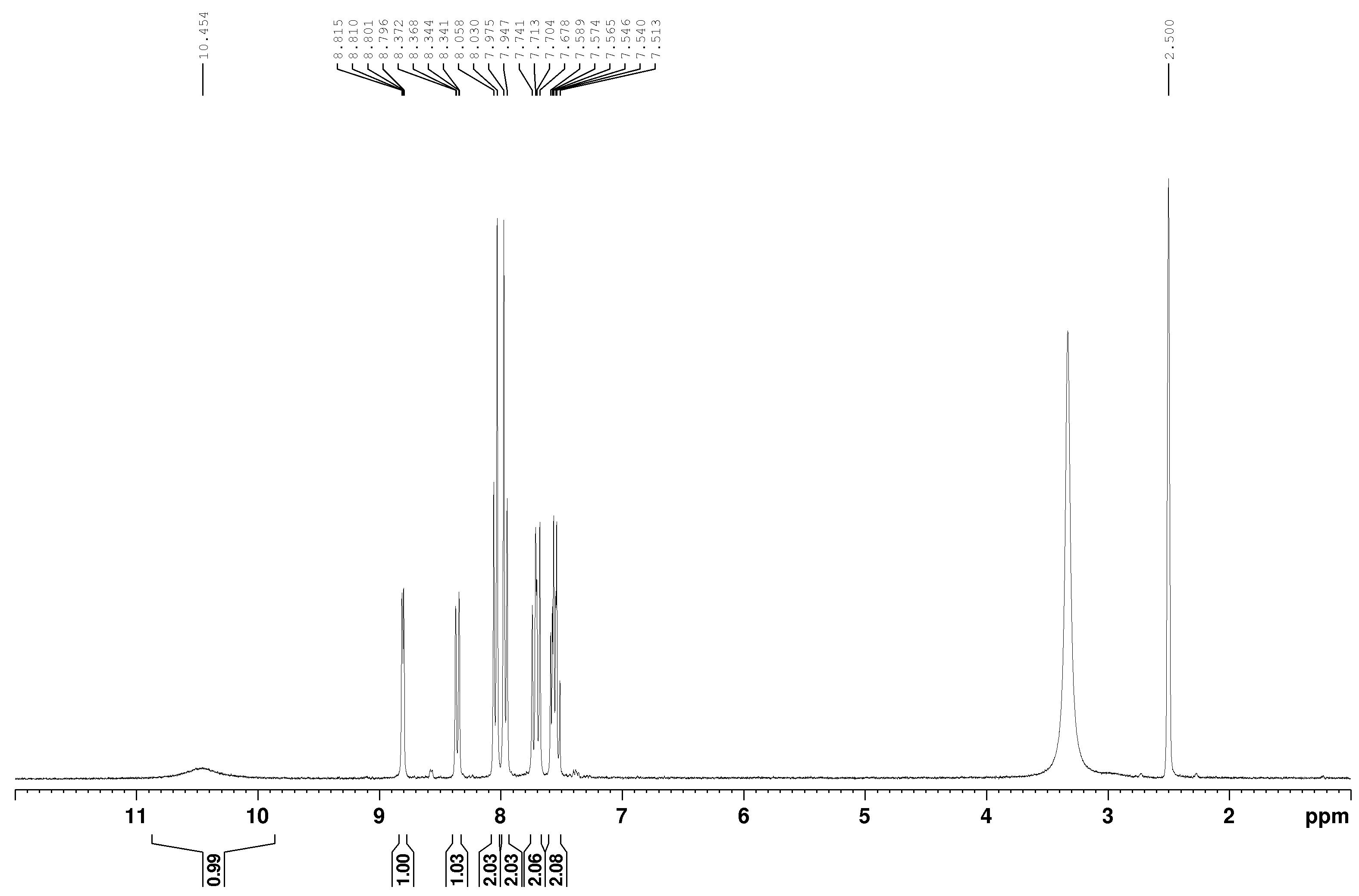


**Fig. S11**. ^1^H NMR spectrum (300 MHz, DMSO-d_6_) of compound **8**.


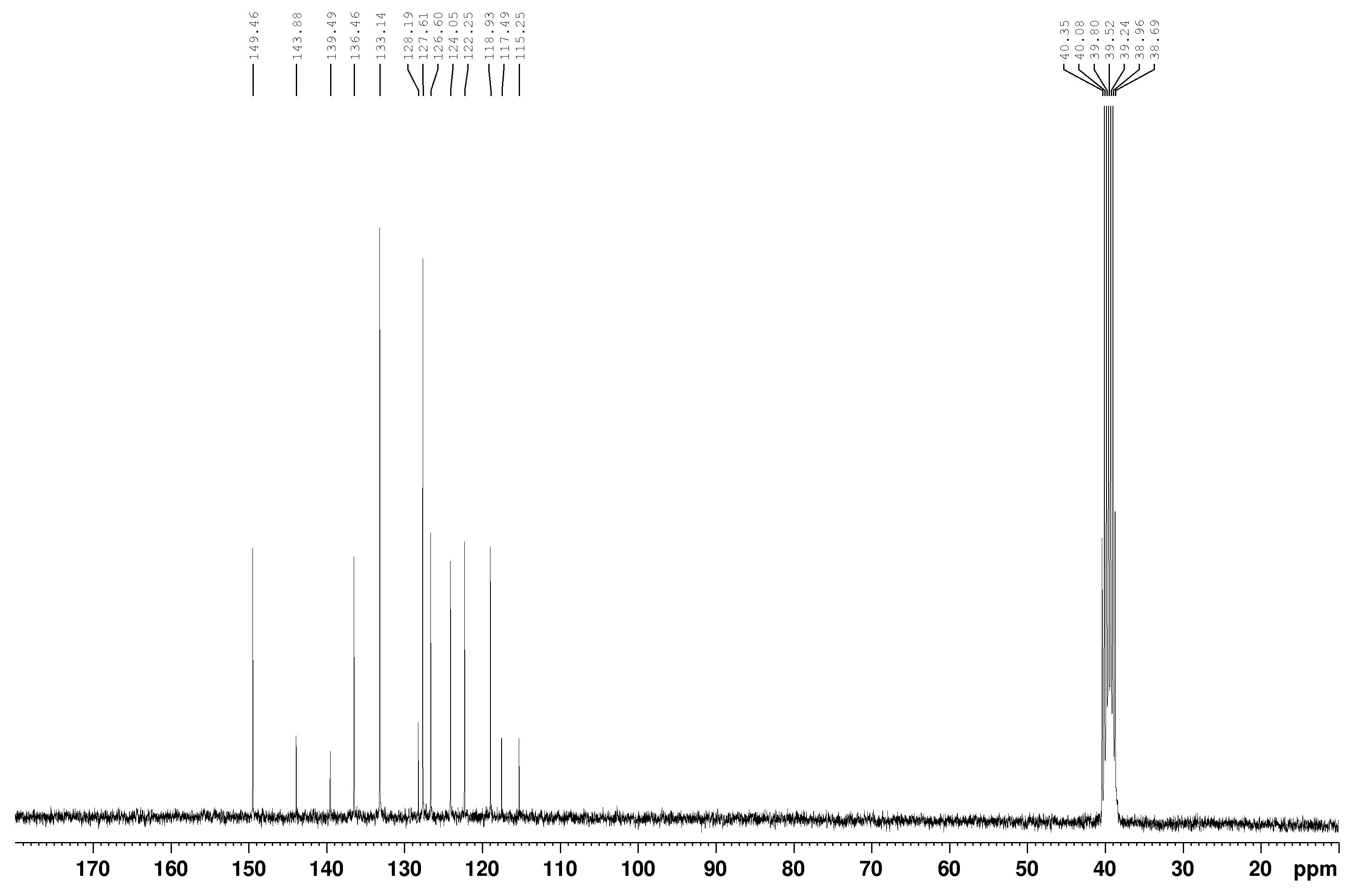


**Fig. S12**. ^13^C NMR spectrum (75 MHz, DMSO-d_6_) of compound **8**.


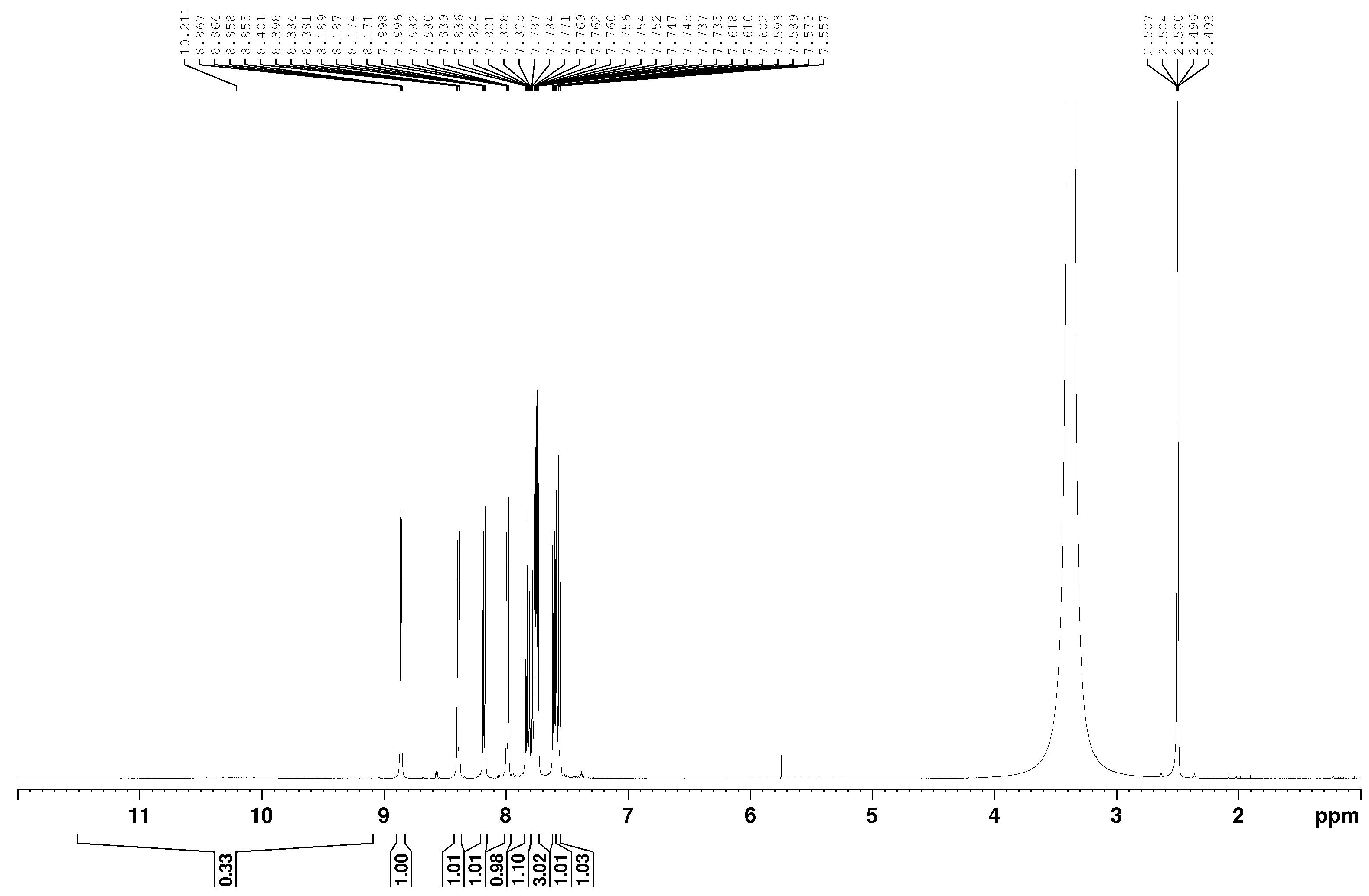


**Fig. S13**. ^1^H NMR spectrum (500 MHz, DMSO-d_6_) of compound **9**.


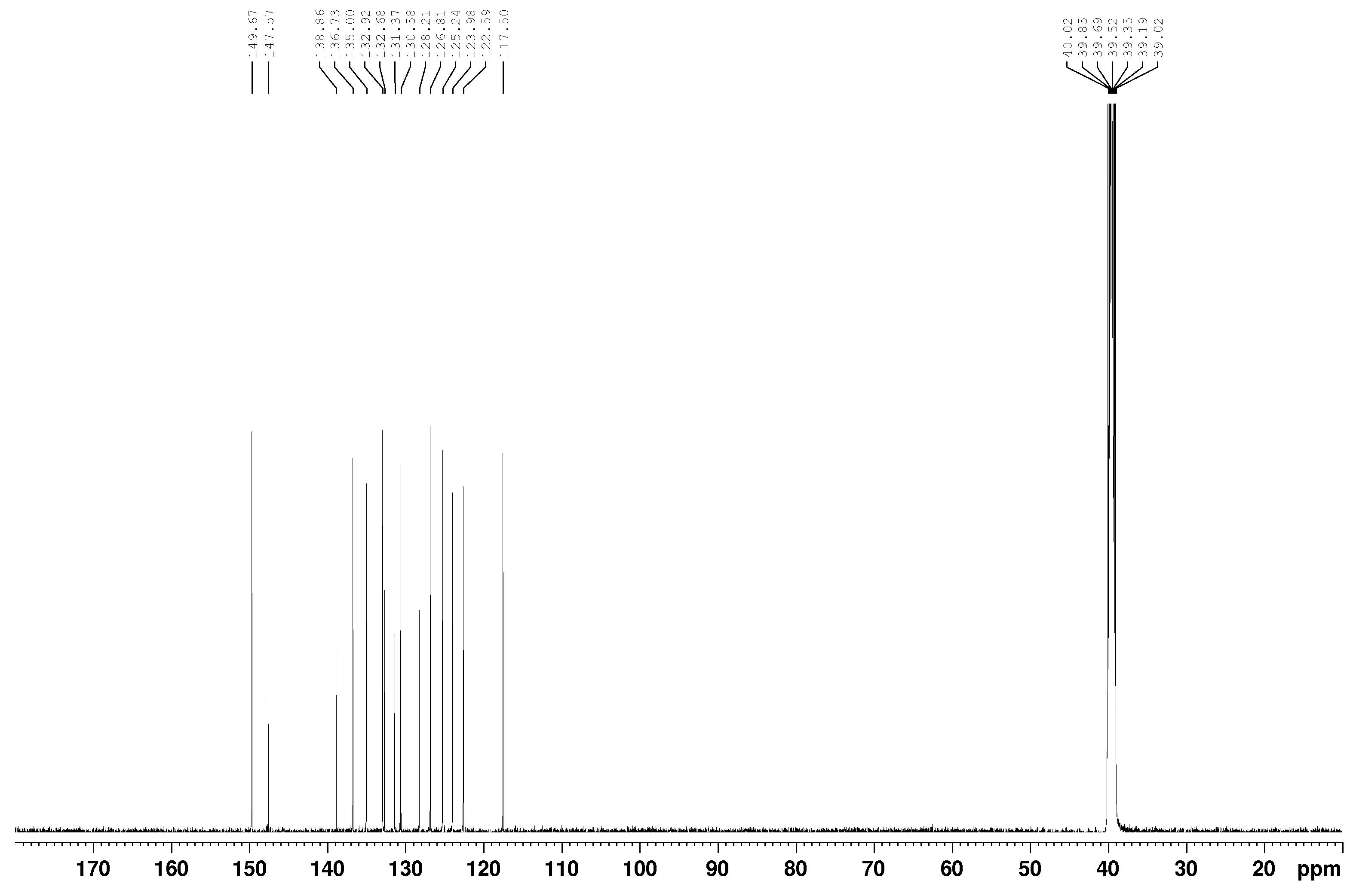


**Fig. S14**. ^13^C NMR spectrum (125 MHz, DMSO-d_6_) of compound **9**.


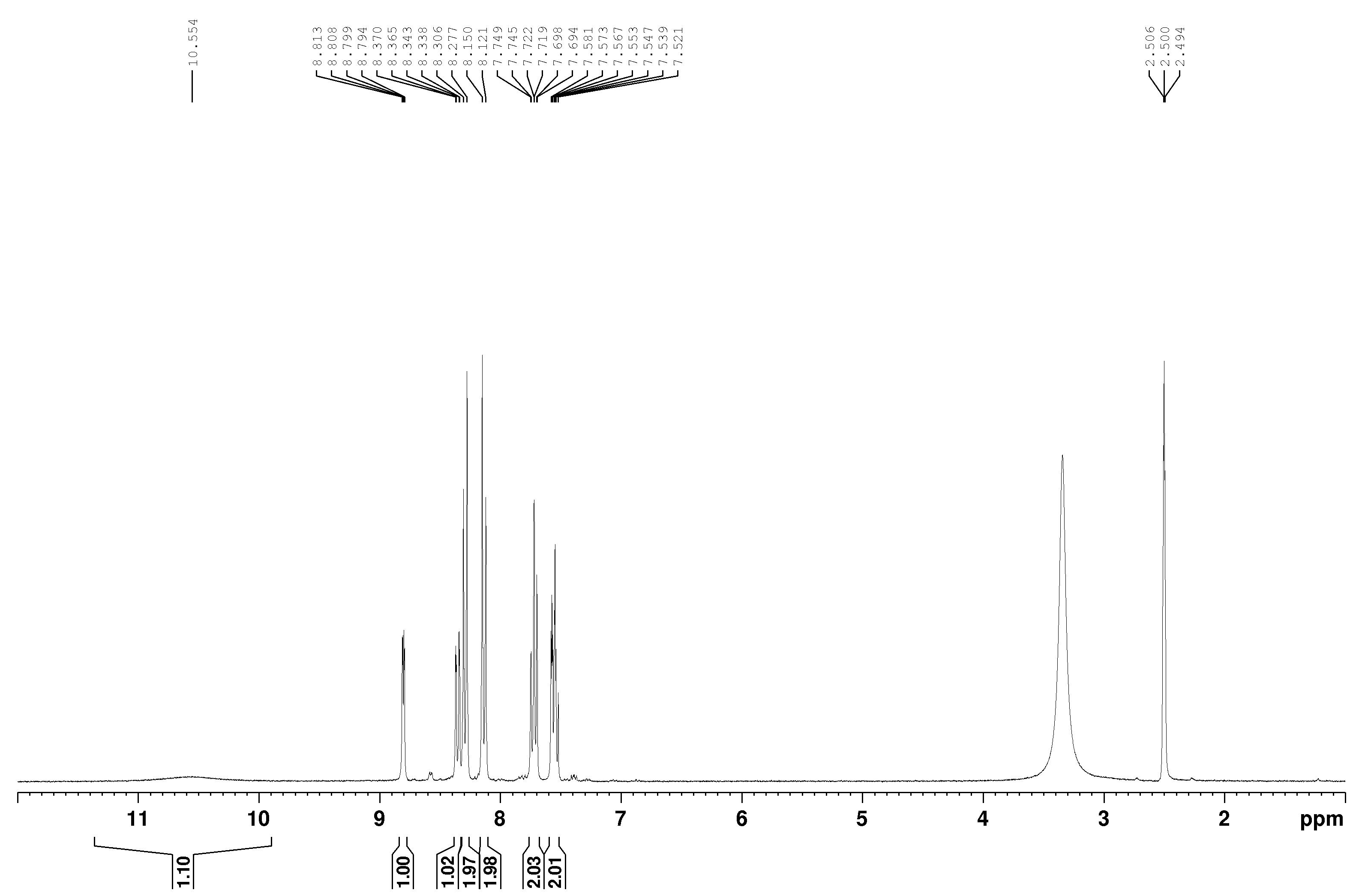


**Fig. S15**. ^1^H NMR spectrum (300 MHz, DMSO-d_6_) of compound **10**.


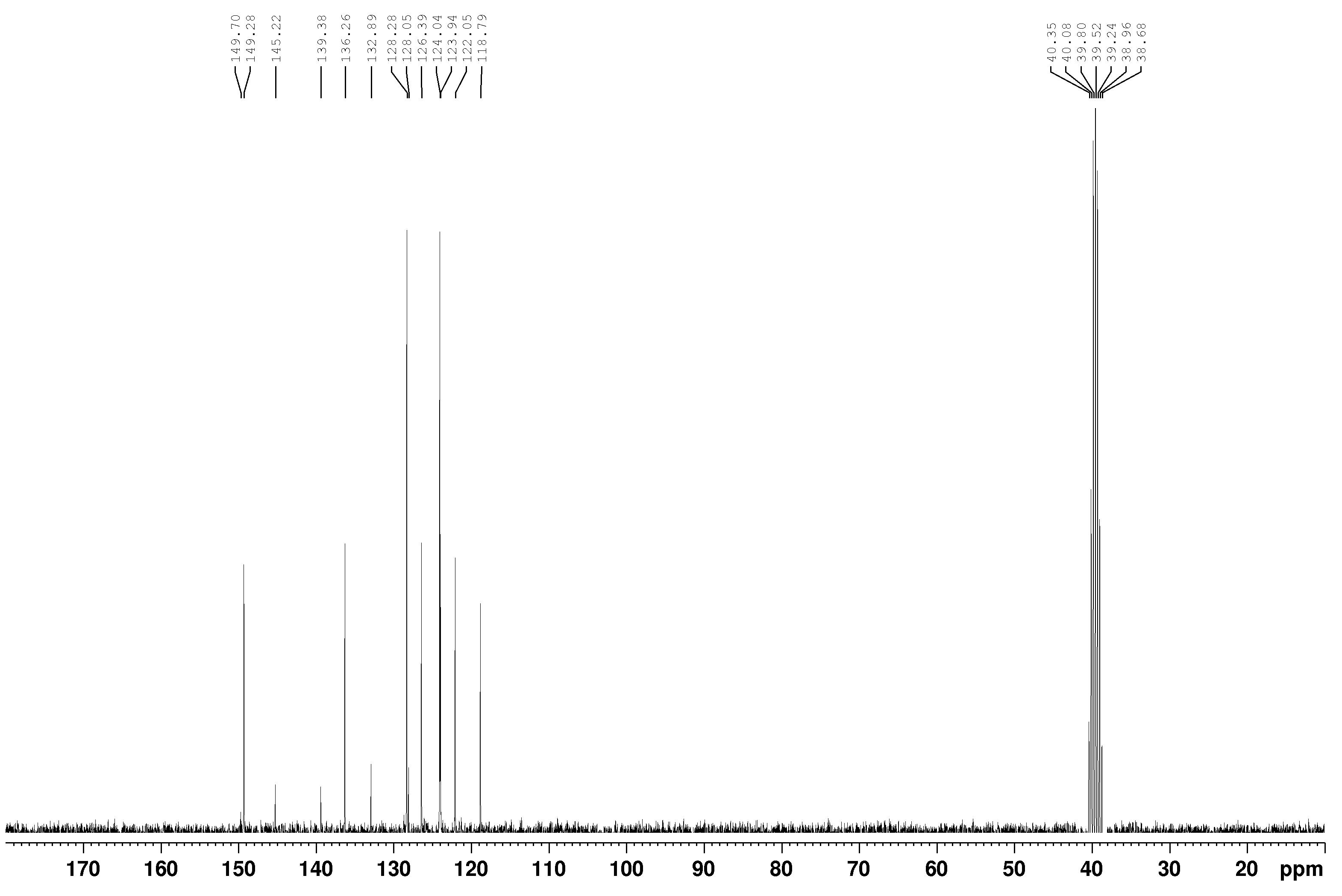


**Fig. S16**. ^13^C NMR spectrum (75 MHz, DMSO-d_6_) of compound **10**.


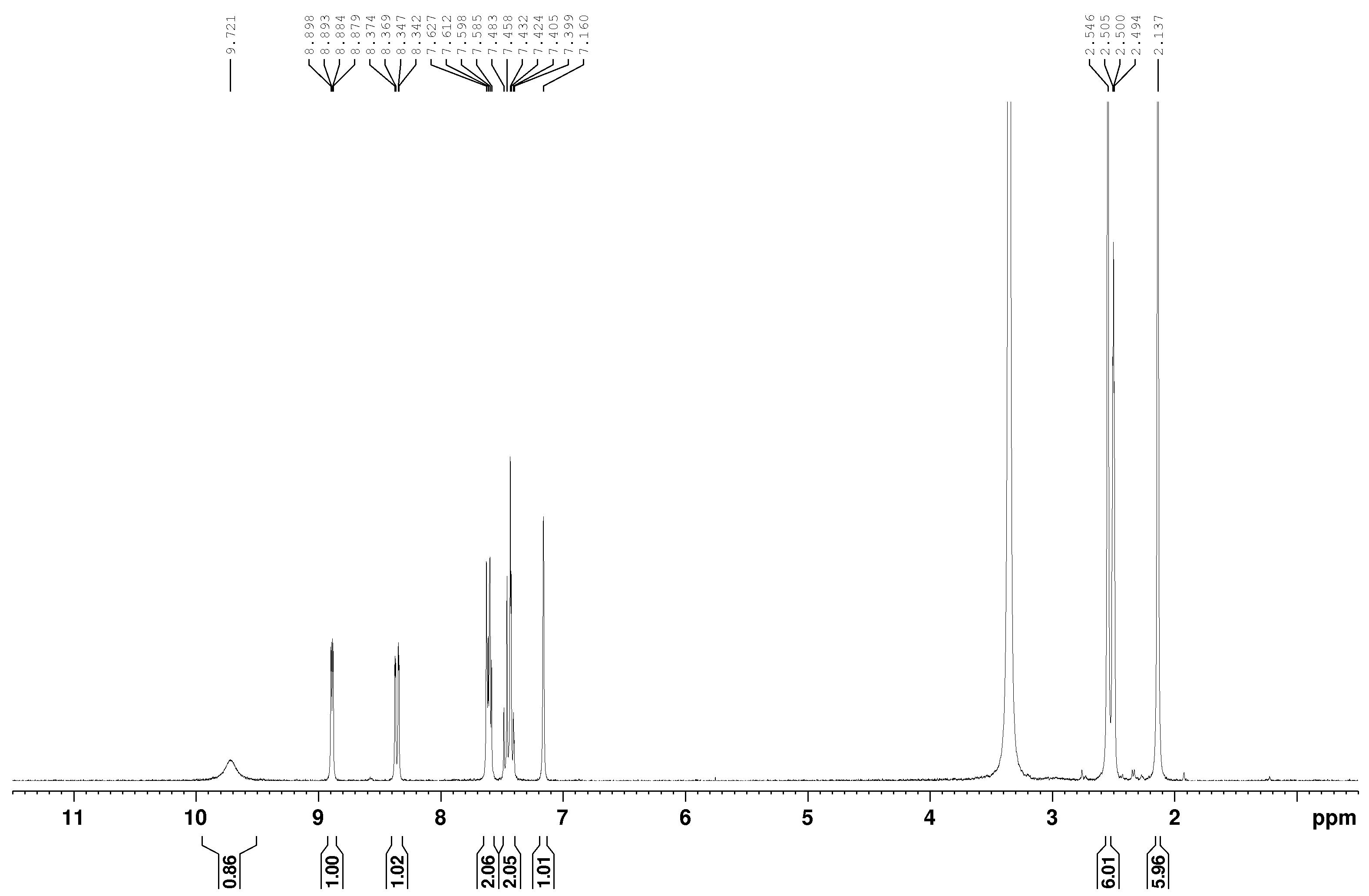


**Fig. S17**. ^1^H NMR spectrum (300 MHz, DMSO-d_6_) of compound **11**.


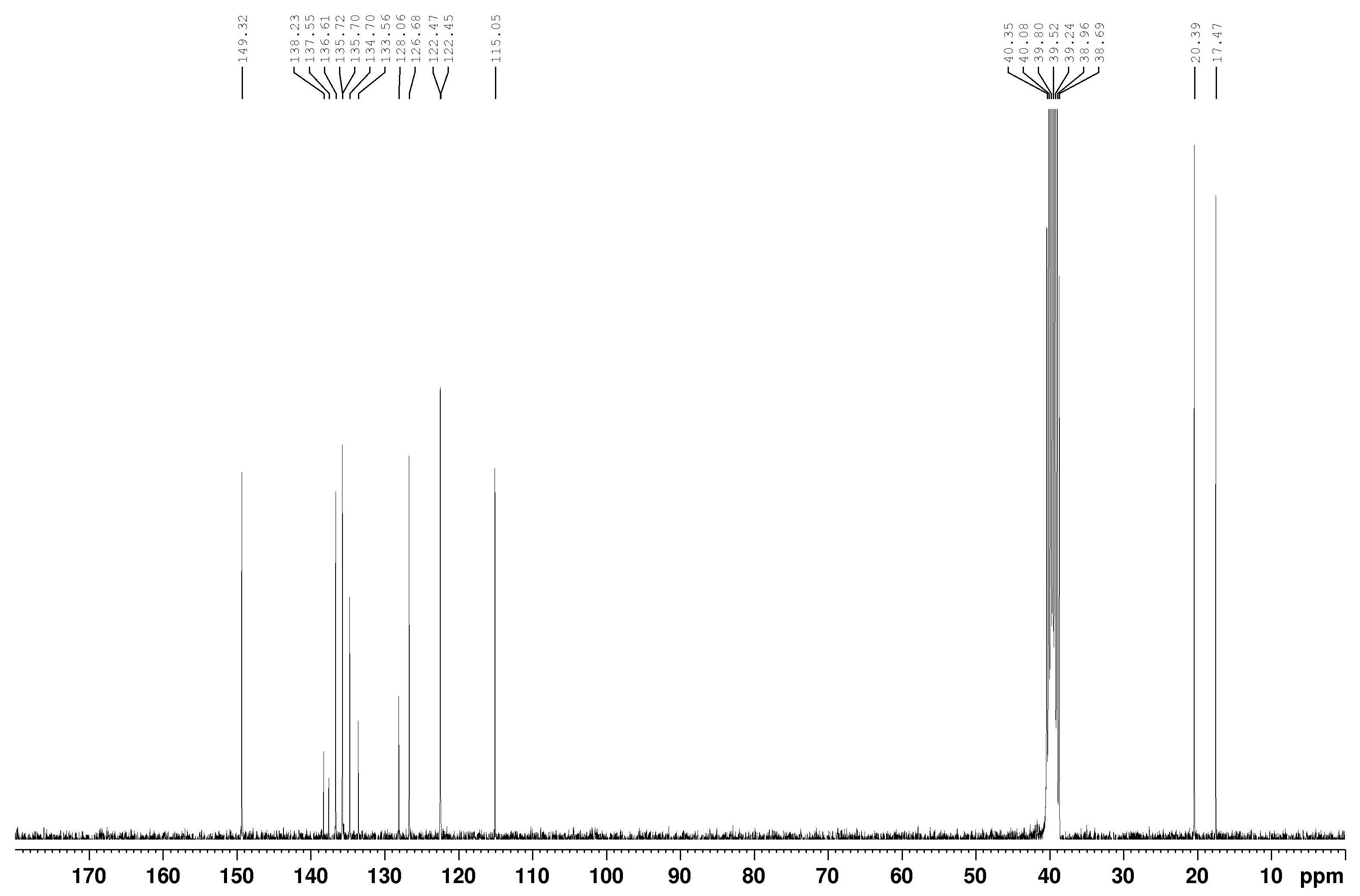


**Fig. S18**. ^13^C NMR spectrum (75 MHz, DMSO-d_6_) of compound **11**.


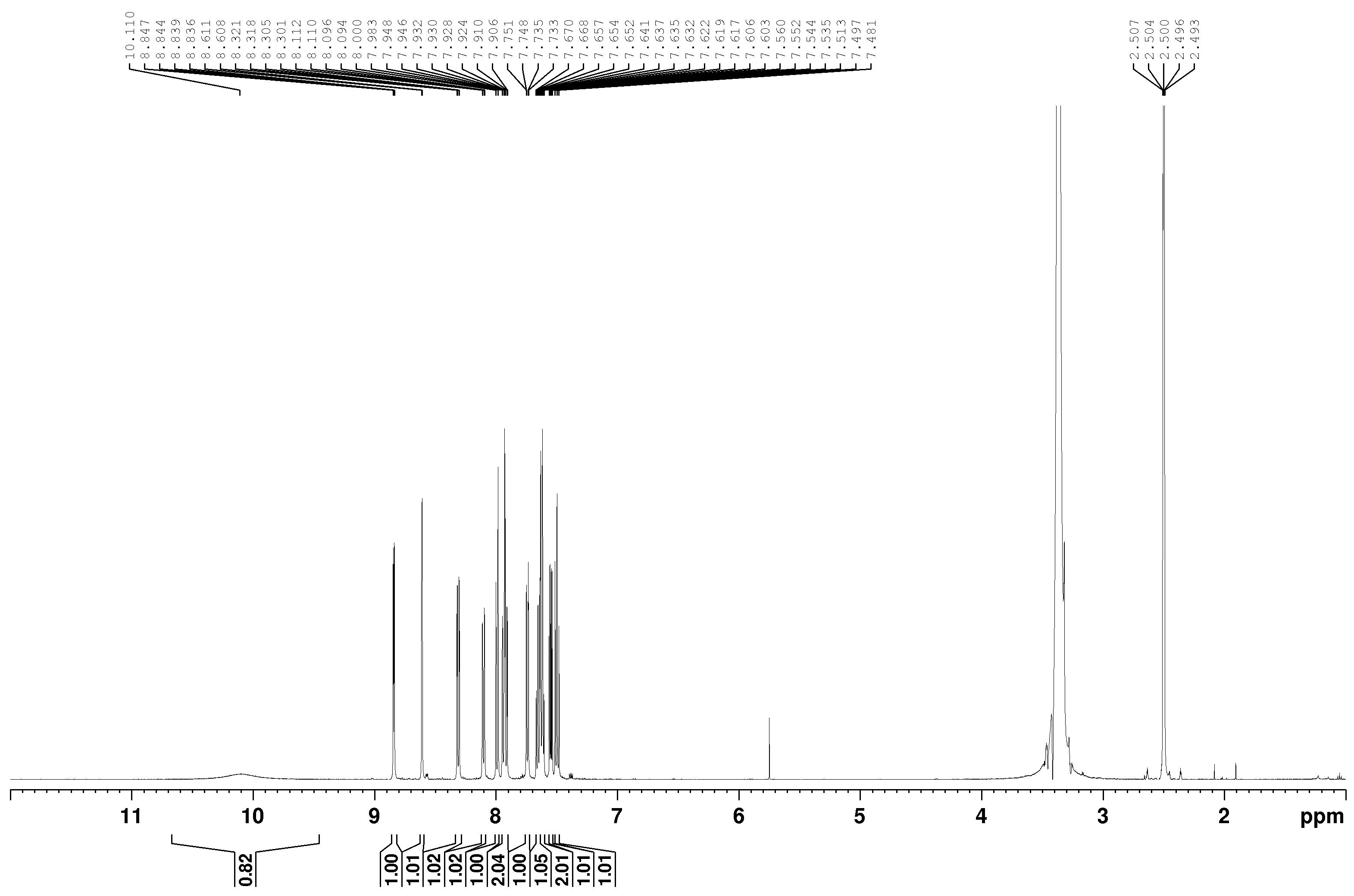


**Fig. S19**. ^1^H NMR spectrum (500 MHz, DMSO-d_6_) of compound **12**.


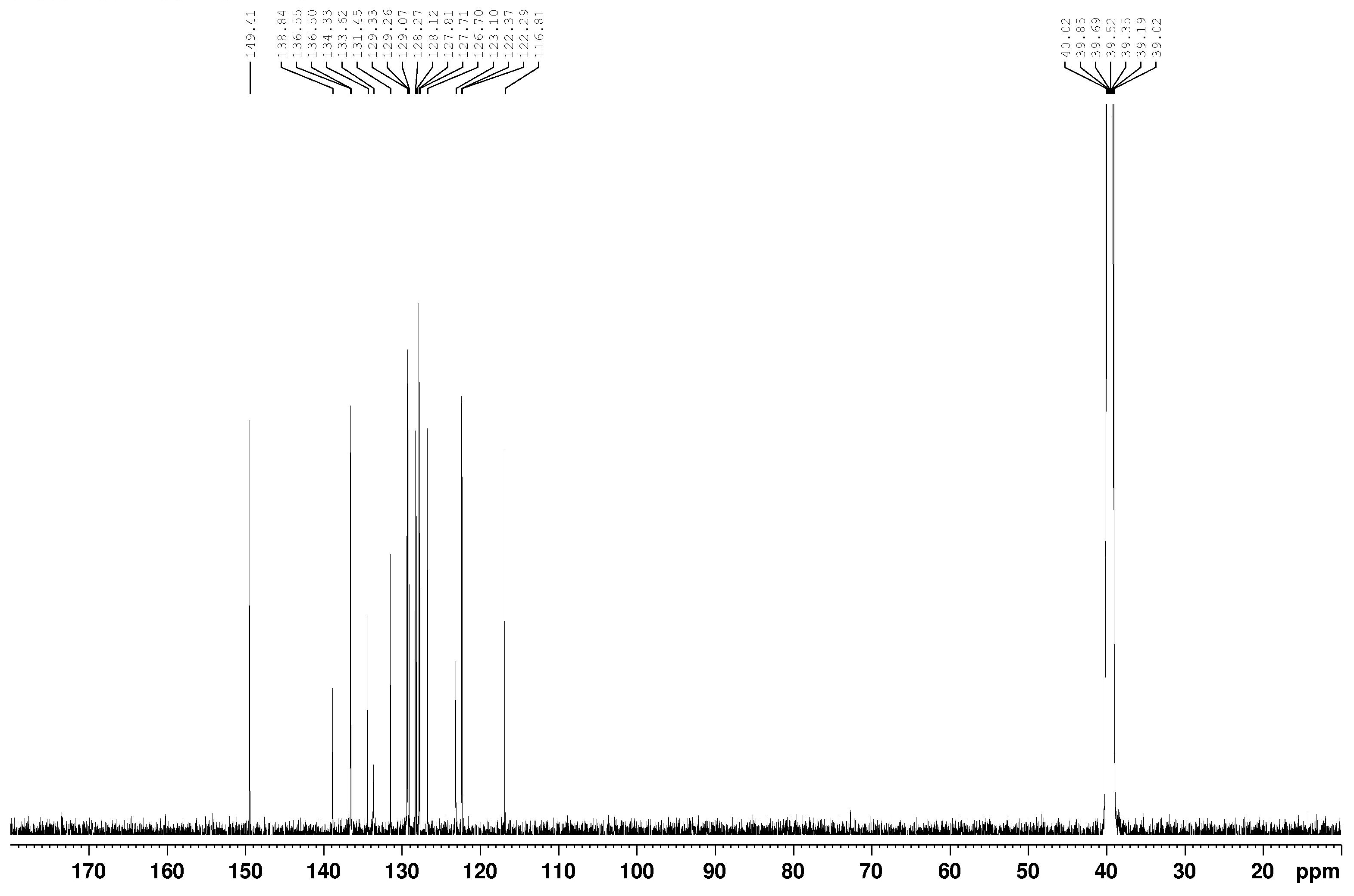


**Fig. S20**. ^13^C NMR spectrum (125 MHz, DMSO-d_6_) of compound **12**.


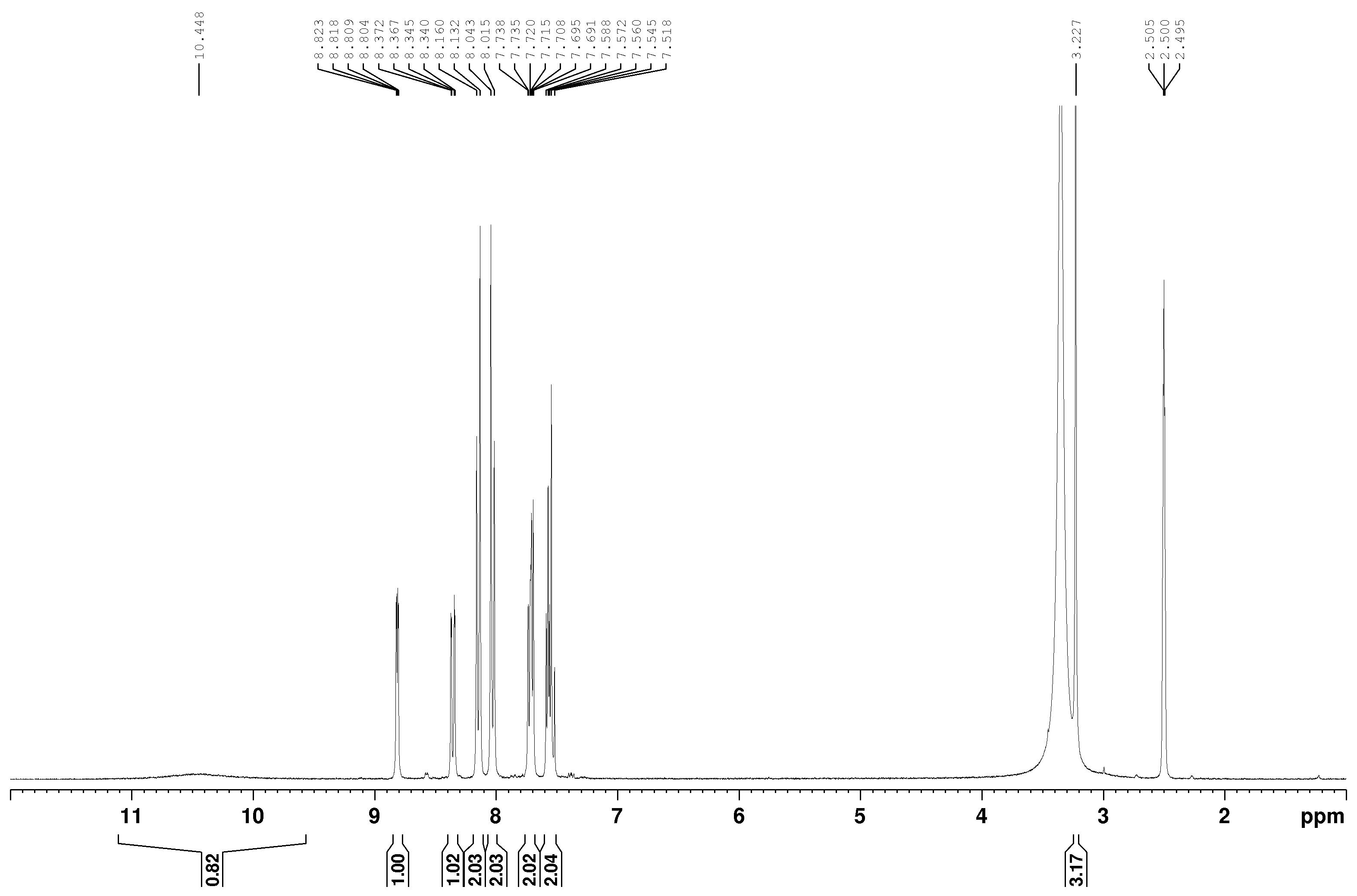


**Fig. S21**. ^1^H NMR spectrum (300 MHz, DMSO-d_6_) of compound **13**.


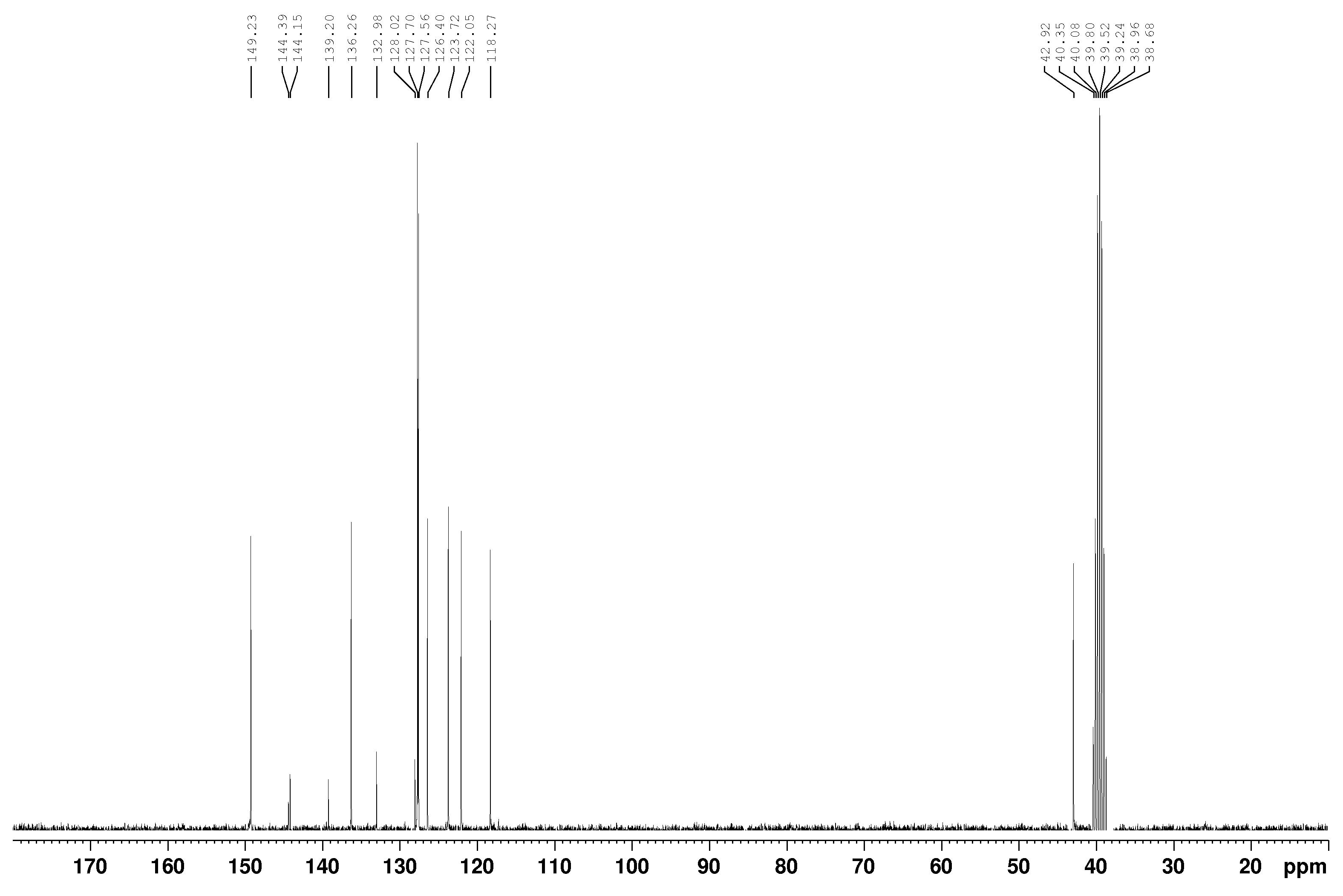


**Fig. S22**. ^13^C NMR spectrum (75 MHz, DMSO-d_6_) of compound **13**.

**Table S1.** Molecular descriptors, experimental and predicted (%DPPH) activity of original 8AQ-based sulfonamides (**3**-**13**).

| Compound | Descriptors | | | | Antioxidant activity (%DPPH) | | | | | |
| --- | --- | --- | --- | --- | --- | --- | --- | --- | --- | --- |
|  |  |  |  |  | LOO-CV | | | 5-fold-CV | | |
|  | ATS5s | GATS1e | Mor04p | Mor24u | Exp. | Pred. | Res. | Exp. | Pred. | Res. |
| **3** | 268.306 | 0.469 | -1.105 | 0.052 | 10.09 | 9.82 | 0.27 | 10.09 | 9.87 | 0.22 |
| **4** | 266.149 | 0.462 | -0.858 | 0.024 | 9.26 | 9.13 | 0.14 | 9.26 | 9.19 | 0.07 |
| **5** | 266.058 | 0.457 | -0.671 | 0.079 | 7.74 | 7.69 | 0.05 | 7.74 | 7.69 | 0.05 |
| **6** | 354.118 | 0.505 | -0.924 | 0.020 | 12.06 | 12.70 | -0.64 | 12.06 | 12.69 | -0.63 |
| **7** | 317.253 | 0.461 | -0.685 | 0.188 | 9.79 | 9.58 | 0.21 | 9.79 | 9.75 | 0.04 |
| **8** | 288.523 | 0.458 | -1.122 | 0.042 | 12.53 | 12.31 | 0.23 | 12.53 | 12.31 | 0.22 |
| **9** | 560.981 | 0.345 | -0.998 | 0.228 | 36.49 | 36.11 | 0.39 | 36.49 | 36.03 | 0.46 |
| **10** | 317.694 | 0.345 | -0.833 | 0.190 | 19.06 | 19.49 | -0.43 | 19.06 | 19.62 | -0.56 |
| **11** | 480.593 | 0.453 | -0.357 | -0.039 | 22.43 | 21.65 | 0.78 | 22.43 | 21.67 | 0.76 |
| **12** | 314.611 | 0.439 | -0.806 | 0.008 | 13.88 | 14.16 | -0.28 | 13.88 | 14.24 | -0.36 |
| **13** | 342.485 | 0.402 | -0.775 | 0.112 | 17.33 | 17.27 | 0.06 | 17.33 | 17.22 | 0.11 |

Exp.: experimental activity

Pred.: Predicted activity

Res.: difference between experimental and predicted values

**Table S2.** Molecular descriptors, experimental and predicted (SOD, pIC_50_) activity of original 8AQ-based sulfonamides (**3**-**13**).

| Compound | Descriptors | | | | Antioxidant activity (SOD, pIC_50_)^a^ | | | | | |
| --- | --- | --- | --- | --- | --- | --- | --- | --- | --- | --- |
|  |  |  |  |  | LOO-CV | | | 5-fold-CV | | |
|  | R1v | AATS8p | B08[C-O] | D211 | Exp. | Pred. | Res. | Exp. | Pred. | Res. |
| **3** | 1.113 | 1.419 | 0 | 0.076 | 3.814 | 3.827 | -0.013 | 3.814 | 3.826 | -0.012 |
| **4** | 1.137 | 1.551 | 0 | 0.076 | ND | ND | ND | ND | ND | ND |
| **5** | 1.154 | 1.622 | 0 | 0.076 | 3.672 | 3.699 | -0.027 | 3.672 | 3.706 | -0.034 |
| **6** | 1.085 | 1.400 | 0 | 0.074 | 3.607 | 3.591 | 0.016 | 3.607 | 3.590 | 0.017 |
| **7** | 1.096 | 1.353 | 1 | 0.074 | 3.601 | 3.621 | -0.020 | 3.601 | 3.621 | -0.020 |
| **8** | 1.164 | 1.499 | 0 | 0.075 | 4.017 | 4.015 | 0.002 | 4.017 | 4.013 | 0.004 |
| **9** | 1.102 | 1.412 | 1 | 0.077 | 3.594 | 3.589 | 0.005 | 3.594 | 3.603 | -0.009 |
| **10** | 1.083 | 1.416 | 1 | 0.074 | 3.348 | 3.373 | -0.025 | 3.348 | 3.357 | -0.009 |
| **11** | 1.074 | 1.480 | 0 | 0.072 | 3.275 | 3.300 | -0.025 | 3.275 | 3.297 | -0.022 |
| **12** | 1.188 | 1.549 | 0 | 0.074 | 4.079 | 4.049 | 0.030 | 4.079 | 4.051 | 0.028 |
| **13** | 1.060 | 1.412 | 1 | 0.074 | 3.221 | 3.179 | 0.042 | 3.221 | 3.184 | 0.037 |

^a^Compound **4** displayed SOD activity < 50%, therefore, its IC_50_ value was not determined and the compound was excluded from the dataset for model construction.

Exp.: experimental activity

Pred.: Predicted activity

ND: Not determined.

**Table S3.** Molecular descriptors and antimicrobial activity of original 8AQ-based sulfonamides (**3**-**13**).

| Compound | Descriptors | | Antimicrobial activity |
| --- | --- | --- | --- |
|  | X4sol | VR2_Dzi |  |
| **3** | 7.092 | 7.503 | Active |
| **4** | 7.398 | 7.555 | Active |
| **5** | 7.564 | 7.577 | Active |
| **6** | 7.231 | 11.729 | Active |
| **7** | 7.757 | 187.643 | Inactive |
| **8** | 7.542 | 9.827 | Active |
| **9** | 8.294 | 7.747 | Inactive |
| **10** | 7.757 | 22.931 | Active |
| **11** | 9.171 | 8.577 | Inactive |
| **12** | 8.771 | 32.716 | Active |
| **13** | 8.447 | 11.021 | Inactive |

The compounds displayed antimicrobial activity with MIC values as defined as active compounds whereas compounds without the activity was assigned as inactive compounds.

**Table S4.** Intercorrelation of key descriptors of the DPPH QSAR model.

|  | ATS5s | GATS1e | Mor04p | Mor24u |
| --- | --- | --- | --- | --- |
| ATS5s | 1.000 |  |  |  |
| GATS1e | -0.467 | 1.000 |  |  |
| Mor04p | 0.241 | 0.053 | 1.000 |  |
| Mor24u | 0.247 | -0.711 | -0.212 | 1.000 |

**Table S5.** Intercorrelation of key descriptors of the SOD QSAR model.

|  | R1v | AATS8p | B10[C-O] | D211 |
| --- | --- | --- | --- | --- |
| R1v | 1.000 |  |  |  |
| AATS8p | 0.720 | 1.000 |  |  |
| B08[C-O] | -0.539 | -0.614 | 1.000 |  |
| D211 | 0.337 | 0.114 | 0.090 | 1.000 |

**Table S6.** Intercorrelation of key descriptors of the decision tree QSPR model.

|  | X4sol | VR2_Dzi |
| --- | --- | --- |
| X4sol | 1.000 |  |
| VR2_Dzi | -0.025 | 1.000 |

**Fig. S23.** The distributions of experimental activities and residual values (differences between experimental and predicted activities) for the DPPH model of LOO‑CV (a) and 5‑fold‑CV (b) sets, and for the SOD model of LOO‑CV (c) and 5‑fold‑CV (d) sets. Training data are represented by black squares, while the LOO‑CV and 5‑fold‑CV sets are represented by white squares.


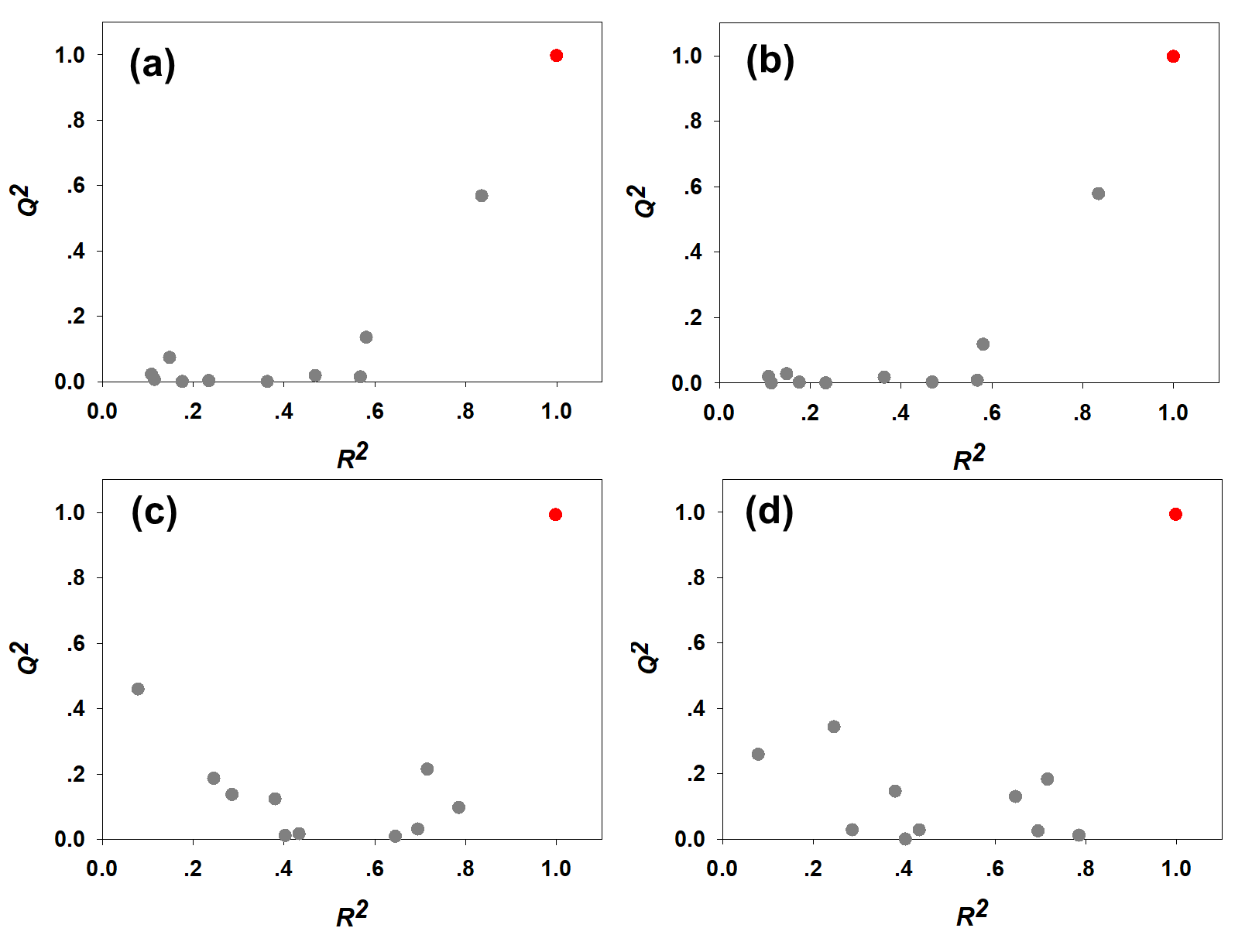


**Fig. S24.** Y-randomization test results for the QSAR models of DPPH activity for (a) LOO‑CV and (b) 5‑fold CV sets, and SOD activity for (c) LOO‑CV and (d) 5‑fold CV sets. The *R^2^* and *Q^2^* values of the original model (red points) are significantly higher than those of the 10 randomized models (grey points).


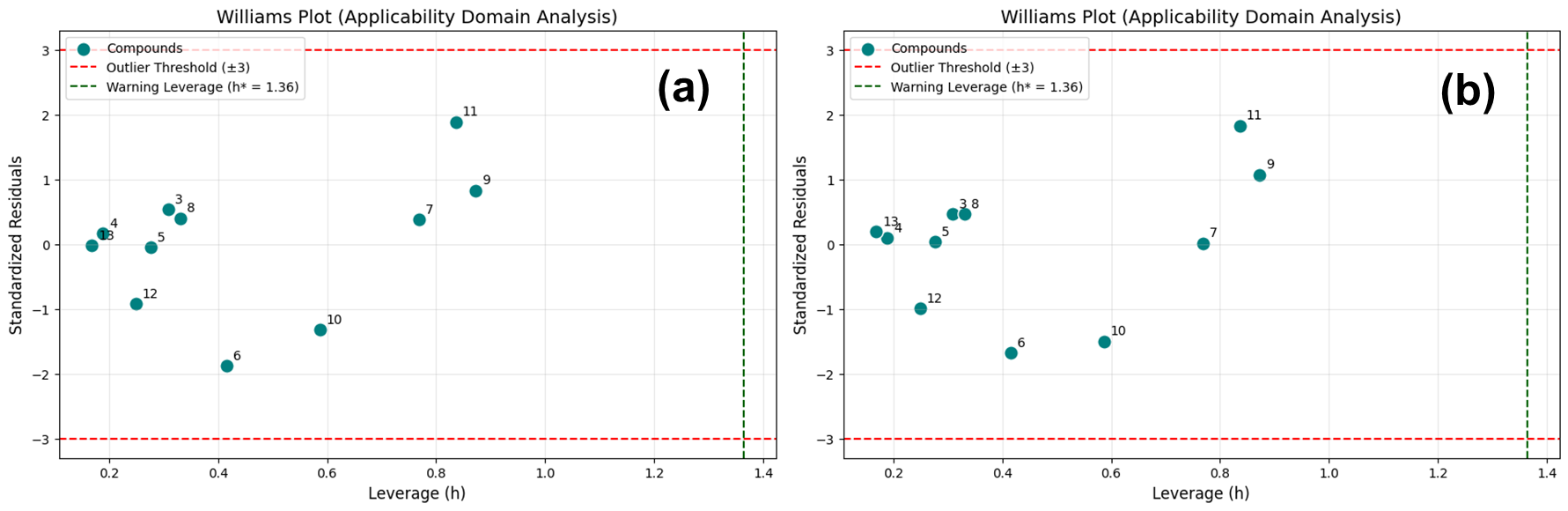


**Fig. S25.** Williams plot of standardized residuals versus leverage values for the QSAR model (DPPH activity: LOO-CV (a) and 5-fold CV (b) sets). The horizontal red dashed lines represent the limit of ± 3 units of standardized residuals, and the vertical green dashed line represents the warning leverage (h* = 1.36). Green cycle symbol represented the tested compounds. Williams plot was performed using Google Colab with Python code.


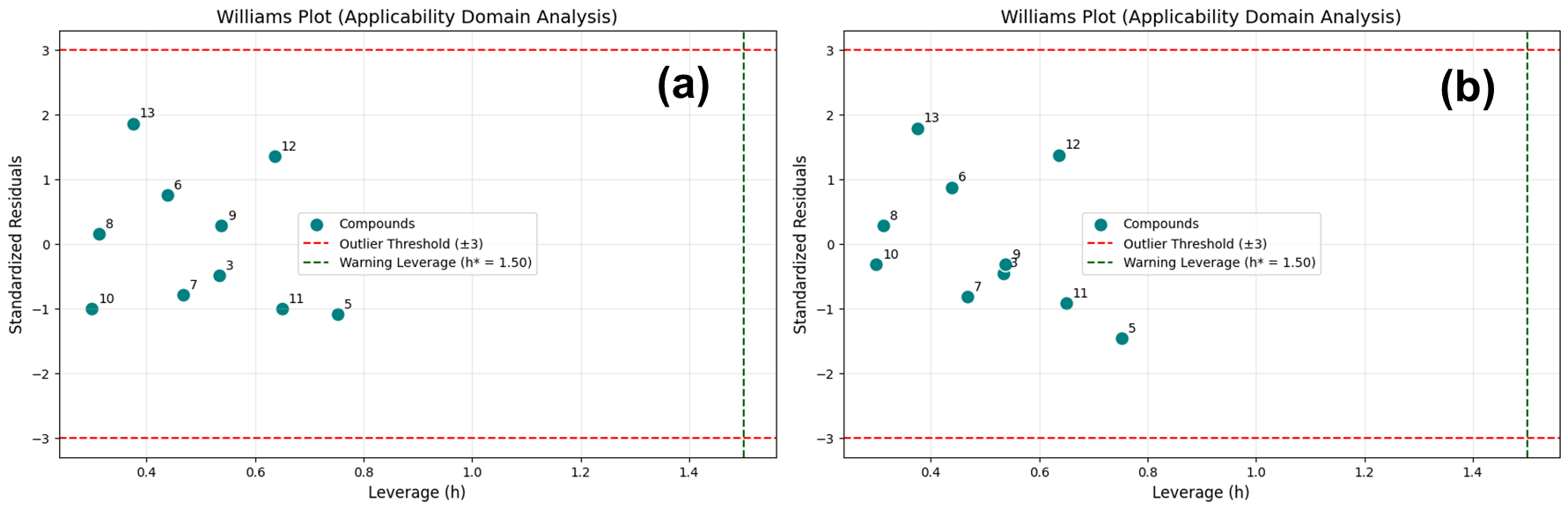


**Fig. S26.** Williams plot of standardized residuals versus leverage values for the QSAR model (SOD activity: LOO-CV (a) and 5-fold CV (b) sets). The horizontal red dashed lines represent the limit of ± 3 units of standardized residuals, and the vertical green dashed line represents the warning leverage (h* = 1.50). Green cycle symbol represented the tested compounds. Williams plot was performed using Google Colab with Python code.

**Table S7.** Statistical summary of antimicrobial model performance.

|  | Accuracy | Precision | Recall | F-measure |
| --- | --- | --- | --- | --- |
| Training set | 90.91 | 0.920 | 0.909 | 0.906 |
| LOO-CV set | 63.64 | 0.691 | 0.636 | 0.642 |
| 5-fold-CV set | 63.64 | 0.691 | 0.636 | 0.642 |

**Table S8.** Confusion matrix for the classification of active and inactive antimicrobial compounds using decision tree analysis.

|  | Training set | | LOO-CV set | | 5-fold-CV set | |
| --- | --- | --- | --- | --- | --- | --- |
|  | Active | Inactive | Active | Inactive | Active | Inactive |
| Active | 7 | 0 | 4 | 3 | 4 | 3 |
| Inactive | 1 | 3 | 1 | 3 | 1 | 3 |


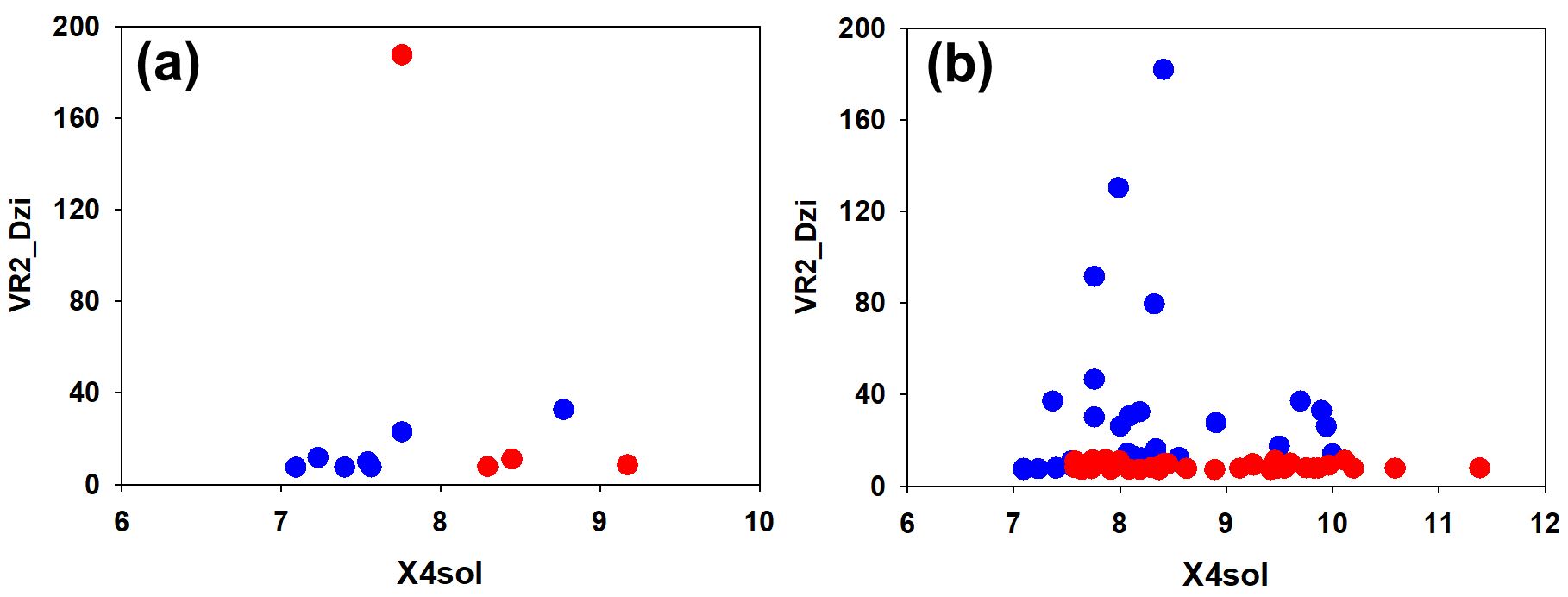


**Fig. S27.** Distribution of antimicrobial classes (i.e., active as blue cycles and inactive as red cycles) of original 8AQ-based sulfonamides (**3**-**13**) (a) and 84 newly designed compounds (b) using decision tree model based on two key molecular descriptors.

**Table S9.** Modification strategies, chemical structures and predicted antioxidant activities (DPPH and SOD) of 84 newly designed 8AQ-based sulfonamide derivatives.

|  | | | | | | | |
| --- | --- | --- | --- | --- | --- | --- | --- |
| Compound | R_1_ | R_2_ | R_3_ | R_4_ | R_5_ | Antioxidant activity | |
|  |  |  |  |  |  | DPPH (%) | SOD (pIC_50_ )^a^ |
| **3** | -H | -H | -F | -H | -H | 10.09 | 3.814 |
| **3a** | -H | -H | -H | -H | -F | 7.83 | 3.850 |
| **3b** | -H | -H | -H | -F | -H | 16.50 | 3.853 |
| **3c** | -H | -H | -F | -H | -F | 4.22 | 3.795 |
| **3d** | -H | -F | -H | -F | -H | 23.21 | 3.838 |
| **4** | -H | -H | -Cl | -H | -H | 9.26 | ND |
| **4a** | -H | -H | -H | -H | -Cl | 10.04 | ND |
| **4b** | -H | -H | -H | -Cl | -H | 9.53 | ND |
| **4c** | -H | -H | -Cl | -H | -Cl | 5.36 | ND |
| **4d** | -H | -Cl | -H | -Cl | -H | 6.10 | ND |
| **4e** | -H | -H | -F | -H | -Cl | 6.62 | ND |
| **4f** | -H | -F | -H | -Cl | -H | 14.29 | ND |
| **4g** | -H | -H | -Cl | -F | -H | 12.87 | ND |
| **4h** | -H | -H | -Cl | -H | -F | 3.82 | ND |
| **4i** | -H | -H | -F | -Cl | -H | 4.82 | ND |
| **5** | -H | -H | -Br | -H | -H | 7.74 | 3.672 |
| **5a** | -I | -H | -H | -H | -H | 14.81 | 2.477 |
| **5b** | -Br | -H | -H | -H | -H | 10.34 | 3.321 |
| **5c** | -H | -Br | -H | -H | -H | 8.55 | 3.608 |
| **5d** | -H | -I | -H | -H | -H | 10.15 | 3.057 |
| **5e** | -H | -H | -I | -H | -H | 4.17 | 3.141 |
| **5f** | -I | -H | -I | -H | -H | 11.92 | 1.893 |
| **5g** | -Br | -H | -Br | -H | -H | 6.42 | 3.110 |
| **5h** | -H | -Br | -H | -Br | -H | 4.18 | 3.176 |
| **5i** | -H | -I | -H | -I | -H | 5.91 | 1.805 |
| **6** | -H | -H | -CF_3_ | -H | -H | 12.06 | 3.607 |
| **6a** | -H | -H | -CH_3_ | -H | -H | 11.49 | 3.590 |
| **6b** | -H | -H | -C_2_H_5_ | -H | -H | 13.96 | 3.873 |
| **6c** | -H | -H | -CH(CH_3_)_2_ | -H | -H | 11.68 | 3.707 |
| **6d** | -H | -H | -OCH_3_ | -H | -H | NA | 3.567 |
| **6e** | -H | -H | -OCF_3_ | -H | -H | 4.89 | 3.637 |
| **6f** | -CF_3_ | -H | -H | -H | -H | 37.22 | 3.902 |
| **6g** | -H | -CF_3_ | -H | -H | -H | 8.20 | 3.659 |
| **6h** | -H | -OCH_3_ | -H | -H | -H | NA | 3.610 |
| **6i** | -OCH_3_ | -H | -H | -H | -H | NA | 3.455 |
| **6j** | -OCF_3_ | -H | -H | -H | -H | 6.61 | 3.398 |
| **6k** | -H | -OCF_3_ | -H | -H | -H | 9.44 | 3.741 |
| **7** | -H | -H | -COCH_3_ | -H | -H | 9.79 | 3.601 |
| **7a** | -H | -H | -COCF_3_ | -H | -H | 9.92 | 3.775 |
| **7b** | -H | -H | -CONH_2_ | -H | -H | 9.16 | 3.515 |
| **7c** | -H | -H | -CONHCH_3_ | -H | -H | 6.40 | 3.249 |
| **7d** | -H | -COCF_3_ | -H | -H | -H | 11.44 | 3.700 |
| **7e** | -H | -CONH_2_ | -H | -H | -H | 11.24 | 3.575 |
| **7f** | -H | -CONHCH_3_ | -H | -H | -H | 9.48 | 3.662 |
| **7g** | -H | -H | -H | -COCH_3_ | -H | 13.45 | 3.254 |
| **7h** | -H | -H | -H | -COCF_3_ | -H | 12.24 | 3.567 |
| **7i** | -H | -H | -H | -CONH_2_ | -H | 10.97 | 3.260 |
| **7j** | -H | -H | -H | -CONHCH_3_ | -H | 9.95 | 3.788 |
| **8** | -H | -H | -CN | -H | -H | 12.53 | 4.017 |
| **8a** | -H | -H | -H | -H | -CN | 17.15 | 4.142 |
| **8b** | -H | -H | -H | -CN | -H | 14.37 | 3.900 |
| **8c** | -H | -H | -CN | -H | -CN | 18.86 | 4.355 |
| **8d** | -H | -H | -COOH | -H | -H | 8.86 | 3.399 |
| **8e** | -H | -H | -H | -H | -COOH | 21.43 | 3.505 |
| **8f** | -H | -H | -H | -COOH | -H | 6.48 | 3.318 |
| **8g** | -H | -H | -COH | -H | -H | 5.94 | 3.559 |
| **8h** | -H | -H | -H | -COH | -H | 12.01 | 3.447 |
| **8i** | -H | -H | -H | -H | -COH | 14.99 | 4.164 |
| **9** | -H | -H | -H | -H | - NO_2_ | 36.49 | 3.594 |
| **9a** | -H | -H | -H | -NO_2_ | -H | 21.82 | 3.376 |
| **9b** | -H | -H | -NO_2_ | -H | -NO_2_ | 40.76 | 3.341 |
| **9c** | -H | -NO_2_ | -H | -NO_2_ | -H | 27.57 | 3.152 |
| **11** | -CH_3_ | -CH_3_ | -H | -CH_3_ | -CH_3_ | 22.43 | 3.275 |
| **11a** | -CH_3_ | -H | -CH(CH_3_)_2_ | -H | -CH_3_ | 23.19 | 3.647 |
| **11b** | -CH_3_ | -H | -C(CH_3_)_3_ | -H | -CH_3_ | 16.21 | 3.679 |
| **11c** | -H | -CH(CH_3_)_2_ | -H | -CH(CH_3_)_2_ | -H | 14.59 | 4.187 |
| **13** | -H | -H | -SO_2_CH_3_ | -H | -H | 17.33 | 3.221 |
| **13a** | -H | -H | -H | -SO_2_CH_3_ | -H | 15.69 | 3.417 |
| **13b** | -H | -H | -SCH_3_ | -H | -H | 8.63 | 3.675 |
| **13c** | -H | -H | -SC_6_H_5_ | -H | -H | 12.75 | 4.793 |
| **13d** | -H | -H | -SOCH_3_ | -H | -H | 13.24 | 3.320 |
| **13e** | -H | -H | -H | -SCH_3_ | -H | 11.18 | 3.668 |
| **13f** | -H | -H | -H | -SC_6_H_5_ | -H | 15.28 | 4.554 |
| **13g** | -H | -H | -H | -SOCH_3_ | -H | 17.25 | 3.296 |

|  | | | | | |
| --- | --- | --- | --- | --- | --- |
| Compound | R_1_ | R_2_ | R_3_ | Antioxidant activity | |
|  |  |  |  | DPPH (%) | SOD (pIC_50_ ) |
| **7** | -H | -H | -H | 9.79 | 3.601 |
| **7k** | -OH | -H | -H | 6.52 | 3.418 |
| **7l** | -OCH_3_ | -H | -H | NA | 3.118 |
| **7m** | -H | -OH | -H | 11.28 | 3.395 |
| **7n** | -H | -OCH_3_ | -H | 0.73 | 3.088 |
| **7o** | -H | -H | -OH | 11.04 | 4.075 |
| **7p** | -H | -H | -OCH_3_ | 2.83 | 3.433 |
| **11** | -H | -H | -H | 22.43 | 3.275 |
| **11d** | -OH | -H | -H | 22.69 | 2.897 |
| **11e** | -OCH_3_ | -H | -H | 10.55 | 2.665 |
| **11f** | -H | -OH | -H | 22.00 | 2.882 |
| **11g** | -H | -OCH_3_ | -H | 8.23 | 2.586 |
| **11h** | -H | -H | -OH | 25.29 | 2.960 |
| **11i** | -H | -H | -OCH_3_ | 12.24 | 2.873 |
| **12** (-S-2- naphthalene) | -H | -H | -H | 13.88 | 4.079 |
| **12a** | -H | -H | -H | 21.22 | 4.128 |
| **12b** | -OCH_3_ | -H | -H | 6.58 | 3.881 |
| **12c** | -H | -H | -OCH_3_ | 1.92 | 4.152 |
| **12d** | -H | -OCH_3_ | -H | 7.52 | 3.934 |
| **12e** | -OCH_3_ | -H | -H | NA | 4.198 |
| **12f** | -H | -H | -OCH_3_ | 5.75 | 3.838 |
| **12g** | -H | -OCH_3_ | -H | 1.03 | 4.003 |

^a^Compound **4** displayed SOD activity < 50%, therefore, its IC_50_ value was not determined and the compound was excluded from the dataset for model construction.

**Fig. S28.** Chemical structures of modified derivatives of prototype **3**.

**Fig. S29.** Chemical structures of modified derivatives of prototype **4**.

**Fig. S30.** Chemical structures of modified derivatives of prototype **5**.

**Fig. S31.** Chemical structures of modified derivatives of prototype **6**.

**Fig. S32.** Chemical structures of modified derivatives of prototype **7**.

**Fig. S33.** Chemical structures of modified derivatives of prototype **8**.

**Fig. S34.** Chemical structures of modified derivatives of prototype **9**.

**Fig. S35.** Chemical structures of modified derivatives of prototype **11**.

**Fig. S36.** Chemical structures of modified derivatives of prototype **12**.

**Fig. S37.** Chemical structures of modified derivatives of prototype **13**.

**Table S10.** Numerical values of molecular descriptors of 84 newly designed compounds for predicting DPPH (%) activity.

| Compound | ATS5s | GATS1e | Mor04p | Mor24u |
| --- | --- | --- | --- | --- |
| **3a** | 295.9814815 | 0.496 | -1.127 | 0.227 |
| **3b** | 393.1481481 | 0.496 | -1.1 | 0.047 |
| **3c** | 292.75 | 0.539 | -1.126 | 0.242 |
| **3d** | 514.7592593 | 0.539 | -1.044 | -0.112 |
| **4a** | 271.5823045 | 0.48 | -1.255 | 0.068 |
| **4b** | 276.6975309 | 0.48 | -0.892 | -0.041 |
| **4c** | 266.1943301 | 0.523 | -1.039 | 0.052 |
| **4d** | 281.8580247 | 0.523 | -0.787 | -0.046 |
| **4e** | 268.350823 | 0.531 | -1.265 | 0.023 |
| **4f** | 398.308642 | 0.531 | -0.868 | -0.072 |
| **4g** | 387.7601738 | 0.531 | -0.948 | 0.025 |
| **4h** | 290.5935071 | 0.531 | -0.909 | 0.204 |
| **4i** | 273.4660494 | 0.531 | -0.953 | 0.046 |
| **5a** | 270.4060741 | 0.431 | -1.522 | 0.102 |
| **5b** | 270.5524691 | 0.464 | -0.993 | 0.006 |
| **5c** | 271.7824074 | 0.464 | -0.721 | 0.026 |
| **5d** | 271.0837037 | 0.431 | -0.852 | 0.158 |
| **5e** | 266.0451029 | 0.431 | -0.208 | 0.304 |
| **5f** | 264.9141399 | 0.431 | -1.164 | 0.132 |
| **5g** | 265.0734739 | 0.496 | -0.812 | 0.012 |
| **5h** | 272.0277778 | 0.496 | -0.77 | 0.216 |
| **5i** | 270.6303704 | 0.431 | -0.523 | 0.343 |
| **6a** | 277.3611111 | 0.429 | -0.884 | 0.111 |
| **6b** | 298.8657407 | 0.427 | -0.996 | 0.097 |
| **6c** | 320.4783951 | 0.425 | -0.654 | 0.25 |
| **6d** | 292.1805556 | 0.644 | -0.875 | 0.186 |
| **6e** | 415.4305556 | 0.669 | -0.906 | -0.03 |
| **6f** | 763.2314815 | 0.57 | -0.994 | -0.079 |
| **6g** | 354.8009259 | 0.57 | -0.995 | 0.008 |
| **6h** | 338.0462963 | 0.644 | -0.77 | 0.099 |
| **6i** | 336.4537037 | 0.644 | -0.764 | 0.225 |
| **6j** | 432.8842593 | 0.669 | -1.186 | 0.066 |
| **6k** | 454.5532407 | 0.669 | -1.231 | -0.023 |
| **7a** | 440.5030864 | 0.593 | -0.767 | 0.096 |
| **7b** | 318.5864198 | 0.509 | -0.847 | 0.026 |
| **7c** | 331.0864198 | 0.541 | -0.979 | 0.193 |
| **7d** | 445.7384259 | 0.593 | -0.757 | -0.005 |
| **7e** | 330.212963 | 0.509 | -0.9 | -0.054 |
| **7f** | 342.8935185 | 0.541 | -1.01 | 0.021 |
| **7g** | 329.2314815 | 0.48 | -1.287 | 0.129 |
| **7h** | 445.7384259 | 0.593 | -0.815 | -0.043 |
| **7i** | 330.212963 | 0.509 | -1.26 | 0.147 |
| **7j** | 342.8935185 | 0.541 | -0.789 | -0.128 |
| **7k** | 352.6450617 | 0.522 | -0.571 | 0.22 |
| **7l** | 367.5617284 | 0.646 | -0.606 | 0.175 |
| **7m** | 352.6450617 | 0.522 | -0.728 | -0.096 |
| **7n** | 367.5617284 | 0.646 | -0.381 | -0.076 |
| **7o** | 419.9104938 | 0.522 | -0.676 | 0.279 |
| **7p** | 399.2345679 | 0.646 | -0.679 | 0.078 |
| **8a** | 398.8148148 | 0.458 | -1.1 | 0.26 |
| **8b** | 316.8425926 | 0.458 | -1.267 | 0.108 |
| **8c** | 433.8009259 | 0.483 | -1.212 | 0.218 |
| **8d** | 319.9197531 | 0.527 | -0.971 | 0.009 |
| **8e** | 544.5833333 | 0.527 | -1.166 | 0.339 |
| **8f** | 331.1944444 | 0.527 | -1.207 | 0.387 |
| **8g** | 295.6944444 | 0.483 | -0.876 | 0.337 |
| **8h** | 314.7407407 | 0.483 | -1.399 | 0.203 |
| **8i** | 437.9814815 | 0.483 | -0.746 | 0.33 |
| **9a** | 334.6851852 | 0.363 | -1.255 | 0.173 |
| **9b** | 635.1388889 | 0.356 | -0.873 | 0.162 |
| **9c** | 425.8333333 | 0.356 | -1.111 | 0.185 |
| **11a** | 462.367284 | 0.422 | -0.675 | 0.131 |
| **11b** | 483.5069444 | 0.421 | 0.271 | 0.364 |
| **11c** | 382 | 0.421 | -0.716 | 0.414 |
| **11d** | 515.9845679 | 0.474 | -0.286 | -0.038 |
| **11e** | 530.9012346 | 0.628 | -0.208 | 0.062 |
| **11f** | 515.9845679 | 0.474 | -0.253 | 0.002 |
| **11g** | 530.9012346 | 0.628 | 0.074 | 0.114 |
| **11h** | 583.4722222 | 0.474 | -0.215 | 0.094 |
| **11i** | 562.7962963 | 0.628 | -0.159 | 0.079 |
| **12a** | 372.0555556 | 0.408 | -0.856 | -0.042 |
| **12b** | 412.3657407 | 0.607 | -0.561 | 0.024 |
| **12c** | 401.6990741 | 0.607 | -0.284 | 0.211 |
| **12d** | 412.3657407 | 0.607 | -0.728 | 0.029 |
| **12e** | 352.2222222 | 0.607 | -0.515 | 0.353 |
| **12f** | 352.2222222 | 0.607 | -0.907 | -0.076 |
| **12g** | 354.9212963 | 0.607 | -0.755 | 0.254 |
| **13a** | 329.0046296 | 0.435 | -0.968 | 0.061 |
| **13b** | 291.2563443 | 0.453 | -0.516 | 0.095 |
| **13c** | 329.3489369 | 0.428 | -0.806 | 0.269 |
| **13d** | 316.852995 | 0.437 | -0.64 | 0.019 |
| **13e** | 288.1388889 | 0.453 | -0.469 | -0.157 |
| **13f** | 326.0123457 | 0.428 | -1.351 | 0.312 |
| **13g** | 307.6265432 | 0.437 | -0.951 | -0.21 |

**Table S11.** Numerical values of molecular descriptors of 84 newly designed compounds for predicting SOD (pIC_50_) activity.

| Compound | R1v | AATS8p | B08[C-O] | D211 |
| --- | --- | --- | --- | --- |
| **3a** | 1.115 | 1.412576 | 0 | 0.076 |
| **3b** | 1.113 | 1.414103 | 0 | 0.077 |
| **3c** | 1.114 | 1.403632 | 0 | 0.073 |
| **3d** | 1.11 | 1.400427 | 0 | 0.076 |
| **4a** | 1.137 | 1.637294 | 0 | 0.076 |
| **4b** | 1.135 | 1.616257 | 0 | 0.077 |
| **4c** | 1.161 | 1.760565 | 0 | 0.073 |
| **4d** | 1.169 | 1.804736 | 0 | 0.076 |
| **4e** | 1.137 | 1.62835 | 0 | 0.073 |
| **4f** | 1.133 | 1.602582 | 0 | 0.076 |
| **4g** | 1.139 | 1.537374 | 0 | 0.075 |
| **4h** | 1.139 | 1.535847 | 0 | 0.073 |
| **4i** | 1.134 | 1.607313 | 0 | 0.075 |
| **5a** | 1.133 | 2.076207 | 0 | 0.076 |
| **5b** | 1.148 | 1.757753 | 0 | 0.076 |
| **5c** | 1.172 | 1.724621 | 0 | 0.077 |
| **5d** | 1.185 | 2.011099 | 0 | 0.077 |
| **5e** | 1.14 | 1.809288 | 0 | 0.076 |
| **5f** | 1.178 | 2.457717 | 0 | 0.073 |
| **5g** | 1.186 | 1.951897 | 0 | 0.073 |
| **5h** | 1.206 | 2.021463 | 0 | 0.076 |
| **5i** | 1.198 | 2.594421 | 0 | 0.076 |
| **6a** | 1.083 | 1.416257 | 0 | 0.076 |
| **6b** | 1.096 | 1.327096 | 0 | 0.075 |
| **6c** | 1.059 | 1.262626 | 0 | 0.074 |
| **6d** | 1.076 | 1.313499 | 1 | 0.075 |
| **6e** | 1.077 | 1.296372 | 1 | 0.076 |
| **6f** | 1.113 | 1.372969 | 0 | 0.075 |
| **6g** | 1.114 | 1.47326 | 0 | 0.074 |
| **6h** | 1.106 | 1.417993 | 1 | 0.077 |
| **6i** | 1.096 | 1.461099 | 1 | 0.078 |
| **6j** | 1.091 | 1.439528 | 1 | 0.075 |
| **6k** | 1.121 | 1.402285 | 1 | 0.076 |
| **7a** | 1.112 | 1.336751 | 1 | 0.074 |
| **7b** | 1.089 | 1.371377 | 1 | 0.074 |
| **7c** | 1.076 | 1.452852 | 1 | 0.075 |
| **7d** | 1.146 | 1.486732 | 1 | 0.074 |
| **7e** | 1.126 | 1.492471 | 1 | 0.076 |
| **7f** | 1.098 | 1.34784 | 1 | 0.075 |
| **7g** | 1.088 | 1.501824 | 1 | 0.076 |
| **7h** | 1.129 | 1.486732 | 1 | 0.074 |
| **7i** | 1.086 | 1.492471 | 1 | 0.076 |
| **7j** | 1.114 | 1.34784 | 1 | 0.075 |
| **7k** | 1.067 | 1.328243 | 1 | 0.073 |
| **7l** | 1.028 | 1.325199 | 1 | 0.073 |
| **7m** | 1.064 | 1.328243 | 1 | 0.073 |
| **7n** | 1.027 | 1.325199 | 1 | 0.072 |
| **7o** | 1.144 | 1.3058 | 1 | 0.073 |
| **7p** | 1.062 | 1.284991 | 1 | 0.071 |
| **8a** | 1.187 | 1.552253 | 0 | 0.078 |
| **8b** | 1.163 | 1.565955 | 0 | 0.077 |
| **8c** | 1.24 | 1.612182 | 0 | 0.075 |
| **8d** | 1.084 | 1.405018 | 1 | 0.074 |
| **8e** | 1.106 | 1.46377 | 1 | 0.077 |
| **8f** | 1.096 | 1.501651 | 1 | 0.076 |
| **8g** | 1.115 | 1.451597 | 1 | 0.075 |
| **8h** | 1.116 | 1.52373 | 1 | 0.077 |
| **8i** | 1.187 | 1.463974 | 1 | 0.078 |
| **9a** | 1.094 | 1.469079 | 1 | 0.076 |
| **9b** | 1.079 | 1.403482 | 1 | 0.073 |
| **9c** | 1.084 | 1.503637 | 1 | 0.073 |
| **11a** | 1.062 | 1.279683 | 0 | 0.072 |
| **11b** | 1.055 | 1.241276 | 0 | 0.072 |
| **11c** | 1.091 | 1.152695 | 0 | 0.073 |
| **11d** | 1.044 | 1.457544 | 1 | 0.071 |
| **11e** | 1.01 | 1.442211 | 1 | 0.071 |
| **11f** | 1.042 | 1.457544 | 1 | 0.071 |
| **11g** | 1 | 1.442211 | 1 | 0.071 |
| **11h** | 1.054 | 1.464463 | 1 | 0.071 |
| **11i** | 1.04 | 1.444803 | 1 | 0.07 |
| **12a** | 1.219 | 1.629486 | 0 | 0.074 |
| **12b** | 1.171 | 1.464474 | 1 | 0.071 |
| **12c** | 1.186 | 1.407252 | 1 | 0.072 |
| **12d** | 1.175 | 1.464474 | 1 | 0.072 |
| **12e** | 1.188 | 1.482131 | 0 | 0.073 |
| **12f** | 1.165 | 1.482131 | 1 | 0.073 |
| **12g** | 1.163 | 1.393182 | 1 | 0.072 |
| **13a** | 1.147 | 1.614209 | 1 | 0.074 |
| **13b** | 1.106 | 1.448256 | 0 | 0.075 |
| **13c** | 1.305 | 1.585195 | 1 | 0.077 |
| **13d** | 1.081 | 1.429322 | 1 | 0.074 |
| **13e** | 1.154 | 1.636647 | 0 | 0.077 |
| **13f** | 1.262 | 1.551648 | 1 | 0.078 |
| **13g** | 1.129 | 1.624988 | 1 | 0.076 |

**Table S12.** Numerical values of molecular descriptors and predicted antimicrobial activity of 84 newly designed 8AQ-based sulfonamide derivatives.

| Compounds | X4sol | VR2_Dzi | Antimicrobial activity |
| --- | --- | --- | --- |
| **3a** | 7.092 | 7.190 | Active |
| **3b** | 7.092 | 7.283 | Active |
| **3c** | 7.092 | 7.691 | Active |
| **3d** | 7.092 | 7.626 | Active |
| **4a** | 8.367 | 7.185 | Inactive |
| **4b** | 7.640 | 7.301 | Inactive |
| **4c** | 9.126 | 7.796 | Inactive |
| **4d** | 8.625 | 7.722 | Inactive |
| **4e** | 8.367 | 7.726 | Inactive |
| **4f** | 7.640 | 7.672 | Inactive |
| **4g** | 7.398 | 8.158 | Active |
| **4h** | 7.398 | 7.757 | Active |
| **4i** | 7.640 | 8.137 | Inactive |
| **5a** | 9.414 | 7.184 | Inactive |
| **5b** | 8.890 | 7.184 | Inactive |
| **5c** | 7.913 | 7.309 | Inactive |
| **5d** | 8.187 | 7.323 | Inactive |
| **5e** | 7.731 | 7.613 | Inactive |
| **5f** | 11.382 | 7.916 | Inactive |
| **5g** | 10.195 | 7.842 | Inactive |
| **5h** | 9.548 | 7.764 | Inactive |
| **5i** | 10.588 | 7.832 | Inactive |
| **6a** | 7.231 | 7.590 | Active |
| **6b** | 7.542 | 10.959 | Active |
| **6c** | 7.757 | 30.195 | Active |
| **6d** | 7.542 | 10.033 | Active |
| **6e** | 7.542 | 8.808 | Active |
| **6f** | 7.843 | 9.405 | Inactive |
| **6g** | 7.366 | 37.111 | Active |
| **6h** | 7.576 | 8.164 | Inactive |
| **6i** | 8.084 | 7.394 | Inactive |
| **6j** | 8.084 | 30.555 | Active |
| **6k** | 7.576 | 10.903 | Inactive |
| **7a** | 7.757 | 8.802 | Inactive |
| **7b** | 7.757 | 46.683 | Active |
| **7c** | 7.993 | 10.989 | Inactive |
| **7d** | 7.740 | 9.116 | Inactive |
| **7e** | 7.740 | 10.384 | Inactive |
| **7f** | 7.985 | 130.392 | Active |
| **7g** | 7.740 | 11.398 | Inactive |
| **7h** | 7.740 | 9.116 | Inactive |
| **7i** | 7.740 | 10.384 | Inactive |
| **7j** | 7.985 | 130.392 | Active |
| **7k** | 8.125 | 13.041 | Active |
| **7l** | 8.450 | 9.758 | Inactive |
| **7m** | 8.070 | 14.343 | Active |
| **7n** | 8.407 | 9.815 | Inactive |
| **7o** | 8.003 | 26.139 | Active |
| **7p** | 8.337 | 16.289 | Active |
| **8a** | 8.084 | 7.332 | Inactive |
| **8b** | 7.576 | 8.069 | Inactive |
| **8c** | 8.554 | 12.487 | Active |
| **8d** | 7.757 | 91.567 | Active |
| **8e** | 8.294 | 8.057 | Inactive |
| **8f** | 7.740 | 11.063 | Inactive |
| **8g** | 7.542 | 10.019 | Active |
| **8h** | 7.576 | 8.129 | Inactive |
| **8i** | 8.084 | 7.352 | Inactive |
| **9a** | 7.740 | 9.710 | Inactive |
| **9b** | 8.902 | 27.672 | Active |
| **9c** | 8.321 | 79.614 | Active |
| **11a** | 9.498 | 17.464 | Active |
| **11b** | 9.603 | 9.995 | Inactive |
| **11c** | 8.321 | 13.232 | Active |
| **11d** | 9.539 | 7.873 | Inactive |
| **11e** | 9.865 | 7.885 | Inactive |
| **11f** | 9.485 | 7.893 | Inactive |
| **11g** | 9.822 | 7.812 | Inactive |
| **11h** | 9.417 | 8.133 | Inactive |
| **11i** | 9.751 | 7.935 | Inactive |
| **12a** | 9.247 | 9.776 | Inactive |
| **12b** | 9.895 | 33.020 | Active |
| **12c** | 9.696 | 37.093 | Active |
| **12d** | 9.940 | 26.059 | Active |
| **12e** | 9.255 | 9.121 | Inactive |
| **12f** | 9.962 | 9.121 | Inactive |
| **12g** | 9.457 | 11.178 | Inactive |
| **13a** | 8.409 | 182.039 | Active |
| **13b** | 7.864 | 11.599 | Inactive |
| **13c** | 9.997 | 14.001 | Active |
| **13d** | 8.186 | 32.486 | Active |
| **13e** | 7.955 | 8.610 | Inactive |
| **13f** | 10.112 | 11.237 | Inactive |
| **13g** | 8.201 | 12.357 | Active |

**Table S13.** Summarized number of newly designed compounds with improved/decreased predicted activities compared to their prototypes.

| Parent compound | Number of modified compound | DPPH activity (%) | | SOD activity (pIC_50_) ^a^ | | Antimicrobial activity | |
| --- | --- | --- | --- | --- | --- | --- | --- |
|  |  | Increased | Decreased | Increased | Decreased | Active | Inactive |
| **** | 4 | 2 | 2 | 3 | 1 | 4 | 0 |
| **** | 9 | 4 | 5 | ND | ND | 2 | 7 |
| **** | 9 | 5 | 4 | 0 | 9 | 0 | 9 |
| **** | 11 | 2 | 9 | 7 | 4 | 7 | 4 |
| **** | 16 | 9 | 7 | 5 | 11 | 7 | 9 |
| **** | 9 | 5 | 4 | 3 | 6 | 3 | 6 |
| **** | 3 | 1 | 2 | 0 | 3 | 2 | 1 |
| **** | 9 | 3 | 6 | 3 | 6 | 2 | 7 |
| **** | 7 | 1 | 6 | 3 | 4 | 3 | 4 |
| **** | 7 | 0 | 7 | 7 | 0 | 4 | 3 |
| Total | 84 | 32 | 52 | 31 | 44 | 34 | 50 |

^a^Compound **4** displayed SOD activity < 50%, therefore, its IC_50_ value was not determined and the compound was excluded from the dataset for SOD model construction.

ND: Not determined.

**Fig. S38**. Summary of 34 newly designed compounds exhibiting antimicrobial activity.

**Fig. S38.** Summary of 34 newly designed compounds exhibiting antimicrobial activity (continued).
